# Air pollution is associated with a distinct pathogen spectrum in community-acquired pneumonia: a prospective multicentre cohort study

**DOI:** 10.1186/s41479-026-00212-3

**Published:** 2026-11-05

**Authors:** Max Kutzinski, Thomas Brenner, Saeed A. Khan, Hendrik Pott, Roman Martin, Barbara C. Weckler, Ann-Christine Link, Christoph Reudenbach, Jörg Bendix, Jan Rupp, Martin Witzenrath, Gernot Rohde, Mathias W. Pletz, Jessica Rademacher, Wilhelm Bertrams, Claus F. Vogelmeier, CAPNETZ study group, André Fuchs, Maximilian Engelmann, Gregor Paul, Mousa Ayoub, Katharina Groehl, Katrin Riedl, Daiana Stolz, Wolfgang Bauer, Eva Corinna Diehl-Wiesenecker, Iris von Wunsch-Rolshoven Teruel, Noah Galtung, Norbert Suttorp, Martin Witzenrath, Christian Wildberg, Caitlin Pley, Enrico Zessin, Sibylle Schmager, Bernhard Schaaf, Julius Kremling, Daniela Nickoleit-Bitzenberger, Harun Azzaui, Martin Hower, Frederik Hempel, Katharina Prebeg, Kalina Popkirova, Martin Kolditz, Bernhard Schulte-Hubbert, Simona Langner, Gernot Rohde, Carla Bellinghausen, Achim Grünewaldt, Adrian Endres, Carlo Frigerio, Benno Fiedler, Marcus Panning, Tobias Welte, Isabell Pink, Nora Drick, Thomas Fühner, Mariet van ’t Klooster, Tabea Steinberg, Grit Barten-Neiner, Waldemar Kröner, Olesya Unruh, Nina Adaskina, Frank Eberhardt, Christina Julius, Thomas Illig, Norman Klopp, Mathias Pletz, Benjamin T. Schleenvoigt, Christina Bahrs, Anne Moeser, Juliane Ankert, Urte Sommerwerck, Tobias Wintermantel, Daniel Drömann, Patrick Parschke, Klaas Franzen, Jan Rupp, Frederike Waldeck, Nadja Käding, Christoph Spinner, Johanna Erber, Florian Voit, Jochen Schneider, Marco Falcone, Giusy Tiseo, David Heigener, Iris Hering, Werner Albrich, Frank Rassouli, Benjamin Wirth, Claus Neurohr, Andreas Essig, Steffen Stenger, Michael Wallner, Heinz Burgmann, Ludwig Traby, Lorenz Schubert, Bernd Schmeck

**Affiliations:** 1 Institute for Lung Research, Universities of Giessen and Marburg Lung Center (UGMLC), Member of the German Center for Lung Research (DZL), University of Marburg, Marburg, Germany; 2 Department of Respiratory, Intensive Care and Sleep Medicine, Clinic for Airway Infections, University Medical Centre Marburg, University of Marburg, Member of the German Center for Lung Research (DZL), Baldingerstraße, 35033 Marburg, Germany; 3 Department of Geography, University of Marburg, Marburg, Germany; 4 Institute of Medical Informatics, University of Münster, Münster, Germany; 5 CAPNETZ STIFTUNG, Hannover, Germany; 6 Infectious Diseases Clinic and Institute of Medical Microbiology, University Hospital Schleswig-Holstein, Lübeck, Germany; 7 Department of Infectious Diseases, Respiratory Medicine and Critical Care, Charité, Member of the German Center for Lung Research (DZL), Universitätsmedizin Berlin, Corporate Member of Freie Universität Berlin and Humboldt-Universität zu Berlin, Berlin, Germany; 8 Biomedical Research in End-stage and Obstructive Lung Disease Hannover (BREATH), Member of the German Center for Lung Research (DZL), Hannover, Germany; 9 Department of Respiratory, Intensive Care and Sleep Medicine, Faculty of Medicine, Marburg-University, Member of the German Center for Lung Research (DZL), Baldingerstraße, 35033 Marburg, Germany; 10 Institute for Infectious Diseases and Infection Control, and Center for Sepsis Care and Control, Jena University Hospital and Friedrich-Schiller-University Jena, Jena, Germany; 11 Pulmonology Süderelbe, Hamburg, Germany; 12 Department of Respiratory Medicine and Infectious Diseases, Hannover Medical School, Hannover, Germany; 13 Institute for Lung Health (ILH), Gießen, Germany; 14 German Centre of Infectious Disease Research (DZIF), Marburg, Germany; 15 Centre for Synthetic Microbiology (Synmikro), University of Marburg, Marburg, Germany

**Keywords:** Community-acquired pneumonia, Air pollution, Environmental exposure, Sputum analysis, Microbiological testing, Pathogen spectrum, *Streptococcus pneumonia*, *Escherichia coli*

## Abstract

**Background:**

Community-acquired pneumonia (CAP) is the leading cause of death due to infection worldwide, with its incidence and disease course influenced by air pollution exposure. Little is known about how air pollution influences microbiological findings in patients with CAP.

**Methods:**

Patients were recruited from a prospective, multicentre study of CAP (CAPNETZ). Air pollution data were collected at the city level considering data up to one month before inclusion. Associations with sputum pathogen detection and cell counts were tested using generalised linear mixed-effects logistic regression models adjusting for clinical confounders, centre-level heterogeneity and meteorological data. Internal validation was performed using an Ensemble Feature Selection (EFS) approach.

**Results:**

Among 4,446 eligible patients with available sputum samples, a potential pathogen was detected in 1,581 patients, while complete sputum-cell-count data were available for 4,014 patients. Detection of *Streptococcus pneumoniae* was associated with previous higher exposure to sulphur dioxide (SO_2_) (OR 1.75, *p* = 0.008) and carbon monoxide (CO) (OR 1.57, *p* = 0.02). *Escherichia coli* was more frequently detected in patients who were exposed to elevated levels of nitrogen dioxide (NO_2_) (OR 2.75, *p* = 0.004) and higher temperature (OR 1.51, *p* = 0.03), whereas increased fine particulate matter (PM_2.5_) exposure (OR 0.37, *p* = 0.001) was linked to reduced detection. Furthermore, SO_2_ exposure influenced the likelihood of obtaining a diagnostically relevant sputum culture and was associated with higher odds of detecting a high leucocyte count (OR 1.18, *p* = 0.02). Results were largely corroborated by EFS, particularly for *S. pneumoniae* and *E. coli* detection.

**Conclusion:**

Exposure to air pollution was associated with a distinct pathogen spectrum in patients with CAP. These findings may have implications for the interpretation of microbiological data in an epidemiological and clinical context and highlight the importance of considering air pollution exposure as a confounding factor. Further validation in cohorts with high-quality environmental, microbiological and antimicrobial resistance data is needed.

**Study registration:**

Registry: German Clinical Trials Register (DRKS), DRKS-ID: DRKS00005274, Registration date: 2013-09-11.

**Supplementary Information:**

The online version contains supplementary material available at https://doi.org/10.1186/s41479-026-00212-3.

## Background

Lower respiratory infections, including community-acquired pneumonia (CAP), remain the leading infectious cause of global mortality, accounting for 2.5 million deaths in 2023 [1]. Air pollution exposure, both short and long-term, has been shown to drive CAP hospitalisation and mortality, particularly affecting populations with risk factors such as older age and multimorbidity [2–9]. With rapid industrialization, urbanisation and climate change further deteriorating air quality and an increasingly aging population, low- and middle-income countries (LMICs) are expected to face unprecedented CAP rates [10–13].

Despite this pressing public health issue, the potential impact of air pollution on microbiological findings in CAP remains poorly understood. This is problematic because guideline-concordant microbiological testing and establishment of microbiological aetiology can improve outcomes and therefore play an important role in the treatment and diagnosis of CAP [14, 15].

Experimental and translational studies suggest that air pollution may influence host-pathogen interactions through several pathways [16]: disruption of the respiratory tract barrier function [17–19], altered alveolar macrophage function [20, 21], changed immune-response [22–25] and dysbiosis of respiratory microbiome [26–28]. These mechanisms raise the possibility that air pollution may not only increase susceptibility to CAP but also shape the pathogen spectrum in respiratory samples. However, previous evidence remains largely confined to laboratory models, with limited translation to the clinical context. Existing clinical research has largely focused on specific pathogens or pollutants in isolation, failing to capture the complexity of environmental and host factors involved [29, 30]. More recently, Kim et al. reported pathogen-specific associations between air pollution and respiratory viral infections in adults with acute exacerbations of chronic obstructive pulmonary disease (COPD), further highlighting the clinical relevance of pollutant-pathogen interactions [31]. Consequently, there is a pressing need for comprehensive studies in well-defined clinical cohorts that utilise multi-pollutant modelling and adjust for clinical confounders.

We therefore investigated the influence of air pollution on microbiological findings using routine sputum samples in a large, multicentric and prospective German cohort of CAP (CAPNETZ).

## Methods

### Study sample

In brief, CAPNETZ is a prospective, observational and multicentre study of CAP enrolling both hospitalized and outpatient treated individuals with a radiographically and clinically confirmed diagnosis of CAP. Exclusion criteria were immunosuppression, nosocomial pneumonia and active tuberculosis. The data set included 10,158 patients who were recruited between 2003 and 2017 (CAPNETZ Project reference: 2024-07-11-CHV6). Written informed consent was obtained from all participants before enrolment. A comprehensive description of the methodology and the population studied has recently been published [32]. A list of the participating centres is provided in the supplement (see Table S1; supplement).

For this analysis, patients with an available sputum sample were eligible. Patients residing in nursing homes and those whose sputum samples were not obtained during the first three days after screening were excluded. The methods for processing sputum samples and laboratory procedures have been described in detail before [33]. Briefly, samples were collected at study inclusion and immediately processed in the local laboratories of the participating clinical centres.

### Air pollution and meteorological data description

Nine meteorological and air quality variables were considered, including six variables representing air pollution: carbon monoxide (CO), nitrogen dioxide (NO_2_), ozone (O_3_), sulphur dioxide (SO_2_), fine particulate matter (PM_2.5_) and black carbon (BC). Additionally, three variables representing meteorological conditions were included as confounders: air pressure, relative humidity and maximum daily temperature. A detailed description of these variables is provided in the supplement (see Table S2; supplement).

The air pollution data originate from the Copernicus Atmosphere Monitoring Service global reanalysis (EAC4) with a spatial resolution of 0.75° x 0.75° [34] due to its broad temporal and geographic availability. CO, NO_2_, O_3_ and SO_2_ are displayed as columnar values, representing the vertically integrated amount of a gas from the ground up to the top of the atmosphere and are therefore not equivalent to ground-level concentrations. PM_2.5_ is reported as a surface concentration variable, while black carbon is represented by dimensionless aerosol optical depth. When using columnar values, however, we assume that there is a relationship between near-surface values and columnar values, as both concentrations are strongly influenced by near-surface emissions and, due to the significant decrease in air density, a large proportion (~ 80%) of the atmospheric gases are located in the atmospheric boundary layer. It can therefore be assumed that high (low) column values correspond to high (low) surface concentrations, and that our models thus react to high and low pollutant levels in relative, but of course not absolute ground level terms. In an exploratory validation analysis, EAC4 total-column values were compared with available ground-level measurements from monitoring stations of the German Environment Agency (UBA) in a limited subset of patients [35].

The meteorological data (2003–2017) from the operational official station monitoring networks of the German Weather Service (DWD) encompass daily mean values of air pressure, relative humidity, and maximum temperature [36, 37]. Quality controls were implemented to verify data consistency and accuracy. Terrain data from the Shuttle Radar Topography Mission (SRTM) were utilized to capture climatological effects and were resampled to 500 × 500 m cells. Ordinary kriging with external drift was applied to interpolate daily climate data for each German municipality, using R software and incorporating SRTM terrain height.

Conditions were linked to each case based on the location of the recruiting centre and date. Data from municipalities within a 1-hour driving distance of the hospital were considered, and a weighted average was calculated based on population and proximity using a distance decay function [38].

Environmental exposure data were assembled for the screening day and the preceding month. The optimal temporal exposure window for associations between air pollution and pathogen-specific respiratory outcomes is not well established. Given that pollutant-related effects on host-pathogen interactions may develop over several days to weeks, a longer exposure window may be relevant for microbiological outcomes. Preliminary lag-specific analyses (see Tables S3 and S4; supplement) suggested that associations with several air pollutants were distributed across multiple days of the investigated exposure period, whereas associations with meteorological variables were more heterogeneous. Recently published evidence, appearing after completion of the present analyses, also supports the relevance of longer lag periods for air pollution exposure in pathogen-specific respiratory outcomes, with the strongest effects observed at a 4-week lag [31]. To ensure a consistent and parsimonious exposure definition within each exposure domain, 31-day median values were used for air pollution variables and 7-day median values for meteorological variables in the primary analyses. Alternative 7-, 15-, and 31-day exposure windows were evaluated in sensitivity analyses. A dummy variable representing the four meteorological seasons was created to account for seasonality.

### Data analysis

The primary outcome of this study was pathogen detection in sputum samples, defined as identification by routine microbiological testing and not implying definitive etiologic attribution. Up to four different pathogens were analysed per sputum sample. Pathogen detection was analysed using binary outcomes (e.g., *S. pneumoniae*, detection vs. detection of other pathogens) focusing on the most frequent pathogens (more than 100 instances) to maintain sufficient statistical power. Analyses of pathogen detection were only performed in patients with positive sputum cultures. If available, analysis of pneumococcal antigen test results in urine was performed. Additionally, the overall rate of positive sputum cultures and sputum cell counts was evaluated (high vs. medium/low epithelial count and high vs. medium/low leukocyte count). High counts were equal to more than 25 cells per field of view (FOV), medium counts equal to ≤ 25 and ≥ 10 cells per FOV and low counts equal to < 10 cells per FOV.

Clinical confounders were age at admission, treatment setting (inpatient vs. outpatient treatment), antibiotic therapy in the four weeks before inclusion, CURB-65 Score ≥ 2 or lower for pneumonia severity, body mass index (BMI), sex, active smoking (in the last 12 months), diagnosis of any respiratory disease or malignancy and pneumococcal and influenza vaccination status [39]. As earlier studies have suggested an association between the detection of specific pathogens and sputum quality, a sputum quality variable was included in the models, labelling sputa with high leucocyte counts and low epithelial cell counts as high-quality sputa [40]. Samples missing epithelial or leukocyte data were excluded from analysis of positive sputum culture rate and sputum cell counts, but for specific pathogen detection, missing data were pragmatically classified as low quality to preserve statistical power. Patients with missing values in one of the clinical variables were excluded. Due to a larger number of missing values for CURB-65 scores, missing values were pragmatically classified as below two in the primary analyses. To assess the robustness of these pragmatic assumptions, we performed a sensitivity analysis using multiple imputation by chained equations (MICE) [41]. Missing CURB-65 scores, sputum leucocyte counts and epithelial-cell counts were imputed across 20 datasets. Rather than imputing the derived binary variables directly, CURB-65 ≥ 2 and high-quality sputum were reconstructed separately within each imputed dataset. The mixed-effects models were fitted in each dataset, and the regression estimates were pooled according to Rubin’s rules. Initially, associations of independent and outcome variables were analysed univariably using either Wilcoxon-Ranked-Sum for continuous and Fisher’s Exact Test for binary variables.

We fitted generalized linear mixed-effects logistic regression models. The base model included a random intercept for the recruiting centre to account for centre-level variability. Centres with fewer than 50 cases were summarized due to low case numbers. Model building followed a forward selection strategy based on Akaike’s Information Criterion (AIC), whereby candidate covariates were added sequentially and retained if they improved model fit by more than 2 AIC units. A dummy variable representing seasonality was first evaluated, followed by clinical covariates, and finally air pollution and confounding meteorological variables. To account for multiple testing, p-values were adjusted with the Benjamin-Hochberg procedure [42]. Models were checked for multicollinearity using the variance inflation factor, accepting values up to five [43]. To evaluate model performance, receiver operating characteristic (ROC) curves and the corresponding area under the curve (AUC) were calculated. In a final step, an Ensemble Feature Selection (EFS) method, consisting of seven different feature selection methods, was applied to assess the stability of findings [44]. When random forests were used in the feature selection process, 100 repetitions were performed. Due to insufficient handling of mixed data types, Gini importance was omitted. When included in generalized linear mixed models and EFS, all continuous variables were standardized using z-score scaling to allow for better interpretation and comparability of effect sizes and usage in feature selection methods.

Statistical analyses were performed in R 4.4.1 (R Core Team, 2021) using the following packages: *lme4* (v1.1-36) [45], *pROC* (v1.18.5) [46], *forestploter* (v1.1.3) [47], *mice* (v3.19.0) [48] and *pheatmap* (v1.0.13) [49]. To increase transparency and reproducibility, a completed STROBE reporting checklist indicating the pages containing the relevant information is provided in the supplement (see Table S5; supplement) [50].

## Results

### General characteristics of the study population and model-building process

**Fig. 1:**
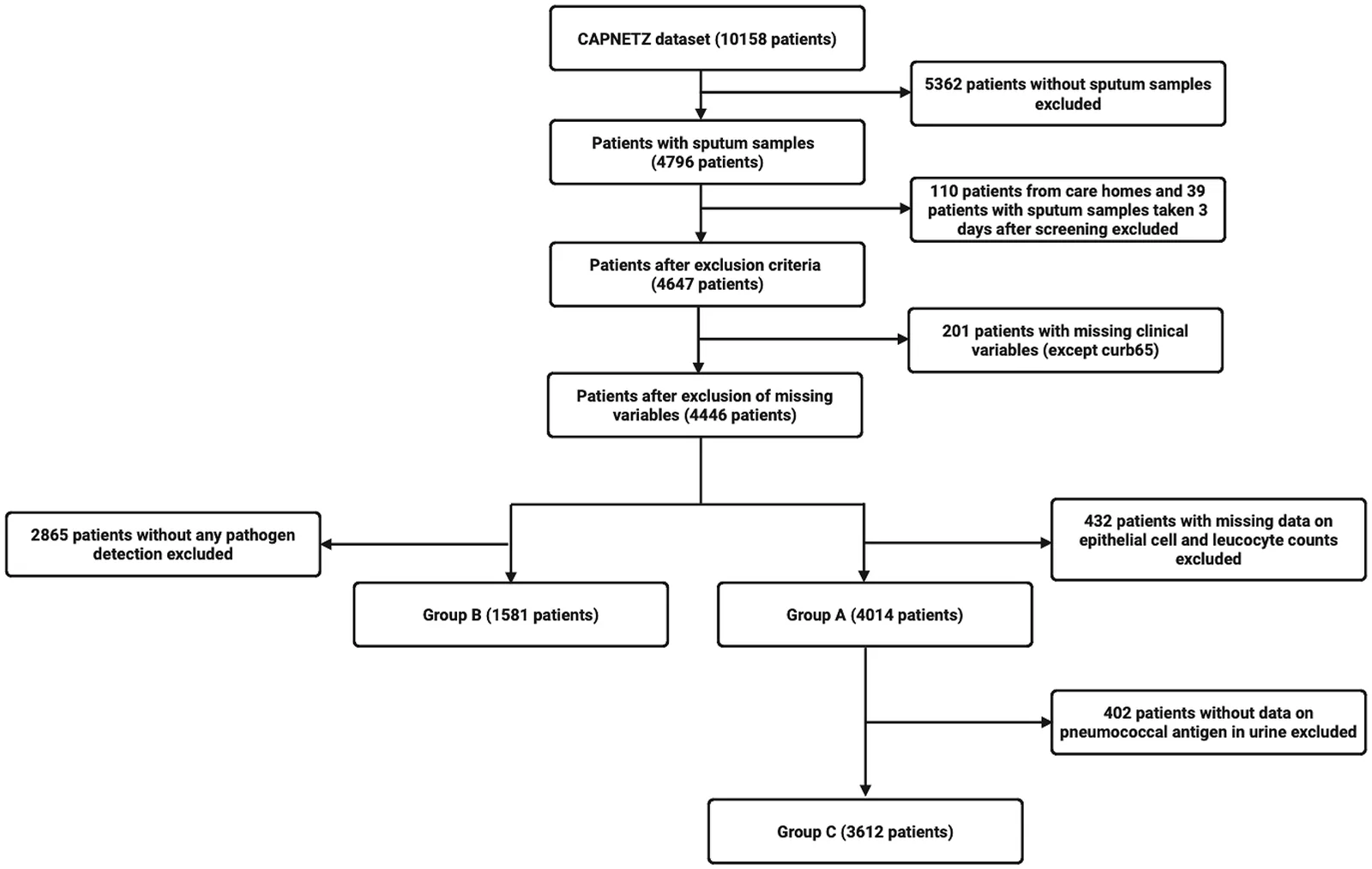
Patient flow chart

Among the 4,446 patients remaining after application of clinical exclusion criteria, 4,014 patients (group A) were eligible for analysis of overall sputum culture positivity and sputum cell counts, 1,581 patients were eligible for the analysis of pathogen detection in sputa (group B) and in 3,612 patients (group C), a pneumococcal antigen test in urine was conducted. The patient flow chart of excluded and included patients is displayed in Fig. 1.

**Fig. 2:**
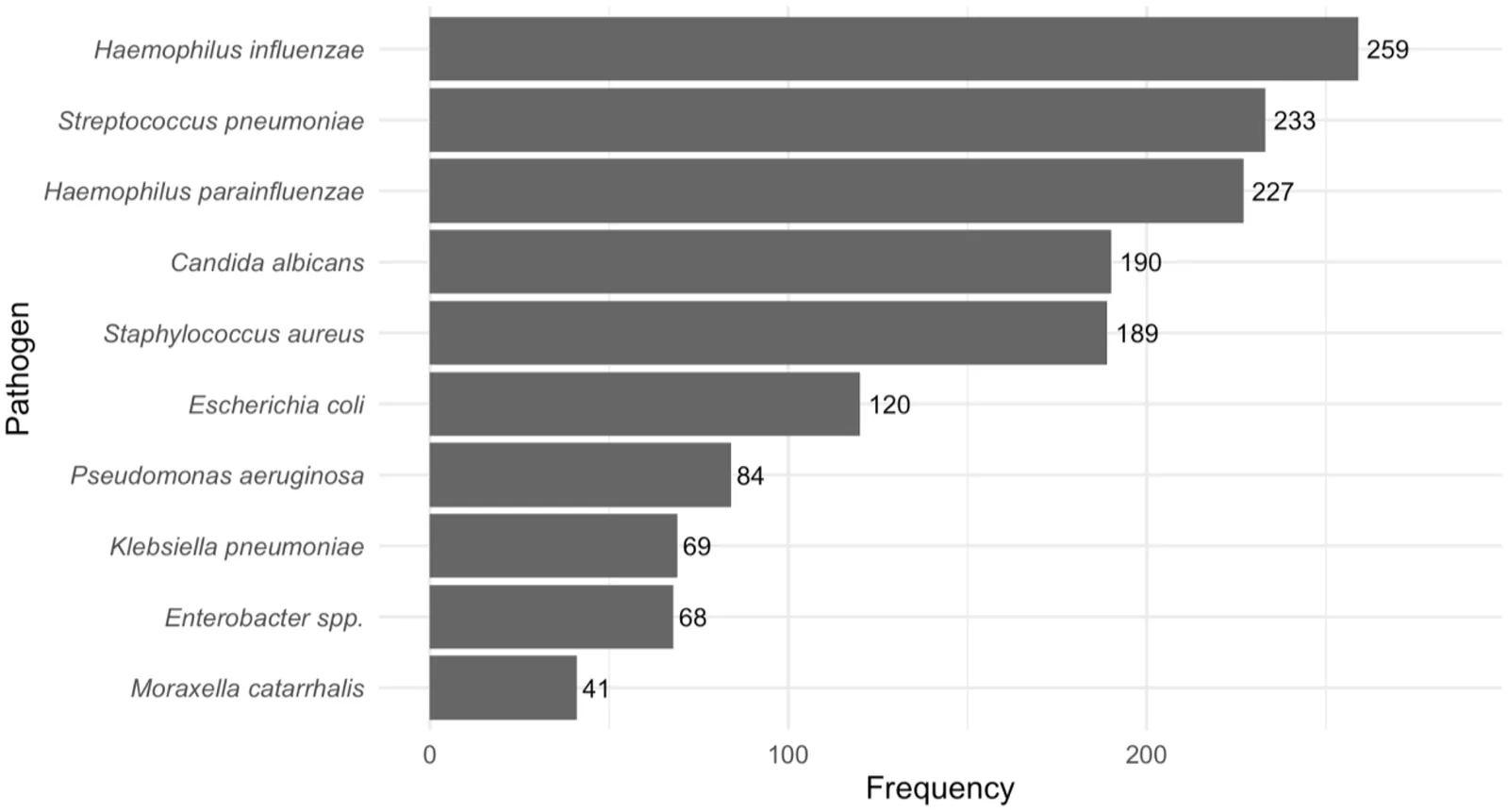
Detection frequency of the most frequent microbial species. Up to four microbial species were assessed per sputum sample. 4251 other pathogens were detected

The clinical characteristics of groups A and B and excluded patients are displayed in Table 1. Patients had a median age of approximately 60 years, were predominantly male and had a borderline normal BMI. Approximately one-third were active smokers and less than 10% had a chronic respiratory disease or diagnosis of malignancy. More than two-thirds of sputum samples were rated as low quality. The majority of patients were admitted to the hospital, less than a third of patients received prior antibiotic therapy and 30-day mortality was low. The six most frequently detected sputum microbial species with sufficient event rates were *Streptococcus pneumoniae*,* Haemophilus influenzae*,* Haemophilus parainfluenzae*,* Candida albicans*,* Staphylococcus aureus*, and *Escherichia coli* (see Fig. 2). As *C. albicans* is not considered a causative pathogen of CAP, analyses involving *C. albicans* were performed as exploratory analyses.

**Table 1:** Clinical characteristics and air pollution and meteorological data

	Group A, *n* = 4,014^a^	Group B, *n* = 1,581^a^	Excluded before formation of analytical populations, *n* = 5,712
Age at admission (years)	61 (44, 72)	64 (46, 73)	67 (49, 78)
Sex (female)	1,709 (43%)	626 (40%)	2551 (45%)
BMI (kg/m^2^)	25 (22, 28.7)	24.9 (22, 28.4)	25 (22.1, 28.7)
Active smoking Missing	1,306 (33%) 0	532 (34%) 0	1,524 (28%) 215
Chronic respiratory disease Missing	248 (6.2%) 0	149 (9.4%) 0	314 (5.5%) 43
Malignancy Missing	355 (8.8%) 0	157 (9.9%) 0	527 (9.3%) 56
Influenza vaccination Missing	1378 (34%) 0	578 (37%) 0	1,770 (35%) 632
Pneumococcal vaccination Missing	562 (14%) 0	262 (17%) 0	575 (11%) 692
Inpatient treatment	2,361 (59%)	961 (61%)	4,668 (82%)
Prior antibiotic therapy Missing	1185 (30%) 0	384 (24%) 0	1,313 (23%) 53
CURB-65			
0 1 2 3 or higher Missing	1,331 (40.7%) 1,055 (32.2%) 653 (20%) 234 (7.1%) 741	463 (35.6%) 434 (33.3%) 291 (22.4%) 114 (8.7%) 279	1,411 (30%) 1,334 (28.3%) 1,137 (24.2%) 826 (17.5%) 1,004
30-day mortality Missing	55 (1.4%) 84	28 (1.8%) 36	310 (5.9%) 440
Positive sputum culture Missing	1,504 (37%) 0	1,581 (100%) 0	133 (40%) 5,379
Epithelial cell count			
Low Medium High Missing	1,881 (47%) 1,097 (27%) 1,036 (26%) 0	804 (52.8%) 405 (26.6%) 313 (20.6%) 59	140 (45.8%) 92 (30%) 74 (24.2%) 5,406
Leucocyte count			
High Medium Low Missing	1,380 (34%) 791 (20%) 1,843 (46%) 0	413 (27.1%) 298 (19.5%) 814 (53.4%) 56	154 (49.7%) 58 (18.7%) 98 (31.6%) 5,402
Sputum quality (high) Missing/Imputed	948 (24%) 0	467 (31.1%) 77	79 (26.4%) 5,413
Pneumococcal antigen in urine Missing	233 (6.5%) 402	121 (8.6%) 179	276 (6.6%) 1,513
PM_2.5_ (µg/m^3^)	27.5 (24, 31.9)	26.9 (23.5, 31)	27.4 (23.5, 32.9)
SO_2_ (mg/kg)	2.34 (1.76, 3.02)	2.29 (1.69, 2.99)	2.47 (1.77, 3.13)
CO (mg/kg)	980 (860, 1080)	982 (859, 1078)	985 (871, 1086)
BC (dimensionless)	0.01 (0.006, 0.016)	0.01 (0.006, 0.016)	0.01 (0.006, 0.016)
NO_2_ (mg/kg)	8.63 (6.56, 10.75)	8.54 (6.51, 10.52)	9.08 (6.94, 11.15)
O_3_ (g/m^2^)	7.27 (6.64, 7.88)	7.37 (6.66, 7.9)	7.24 (6.58, 7.91)
Relative humidity (%)	80 (72, 86)	80 (72, 85)	80 (72, 86)
Air pressure (hPa)	1,001 (992, 1,007)	1,002 (994, 1,009)	1,001 (993, 1008)
Maximum temperature (°C)	13 (6, 20)	13 (6, 20)	13 (7, 20)

^a^ See Fig. 1 for details of patient selection and definition of the analytical populations; Groups A and B are not mutually exclusive; percentages were calculated among all patients with available data; values as median (Q1, Q3) or n (%); air pollutants are displayed as 31-day median values and meteorological variables as 7-day median values; CO, NO_2_, O_3_ and SO_2_ represent total-column quantities and are not equivalent to ground-level concentrations; PM_2.5_ is reported as a surface concentration variable, while black carbon is represented by dimensionless aerosol optical depth

Univariable analysis of outcomes revealed significant associations with both clinical and environmental variables (see S6a, b; supplement). A table showing the model building process with respective AIC values is provided in the supplement (see Table S7; supplement).

Air pollution and meteorological variables that had a significant impact across outcomes, included SO_2_, NO_2_, CO, PM_2.5_ and humidity. No significant associations were found for O_3_ and BC. Among clinical variables, prior antibiotic therapy, sputum quality, treatment setting, age, sex, BMI and diagnosis of a respiratory disease were consistently retained in the final models.

### Pathogen spectrum and sputum characteristics are associated with air pollution

**Fig. 3:**
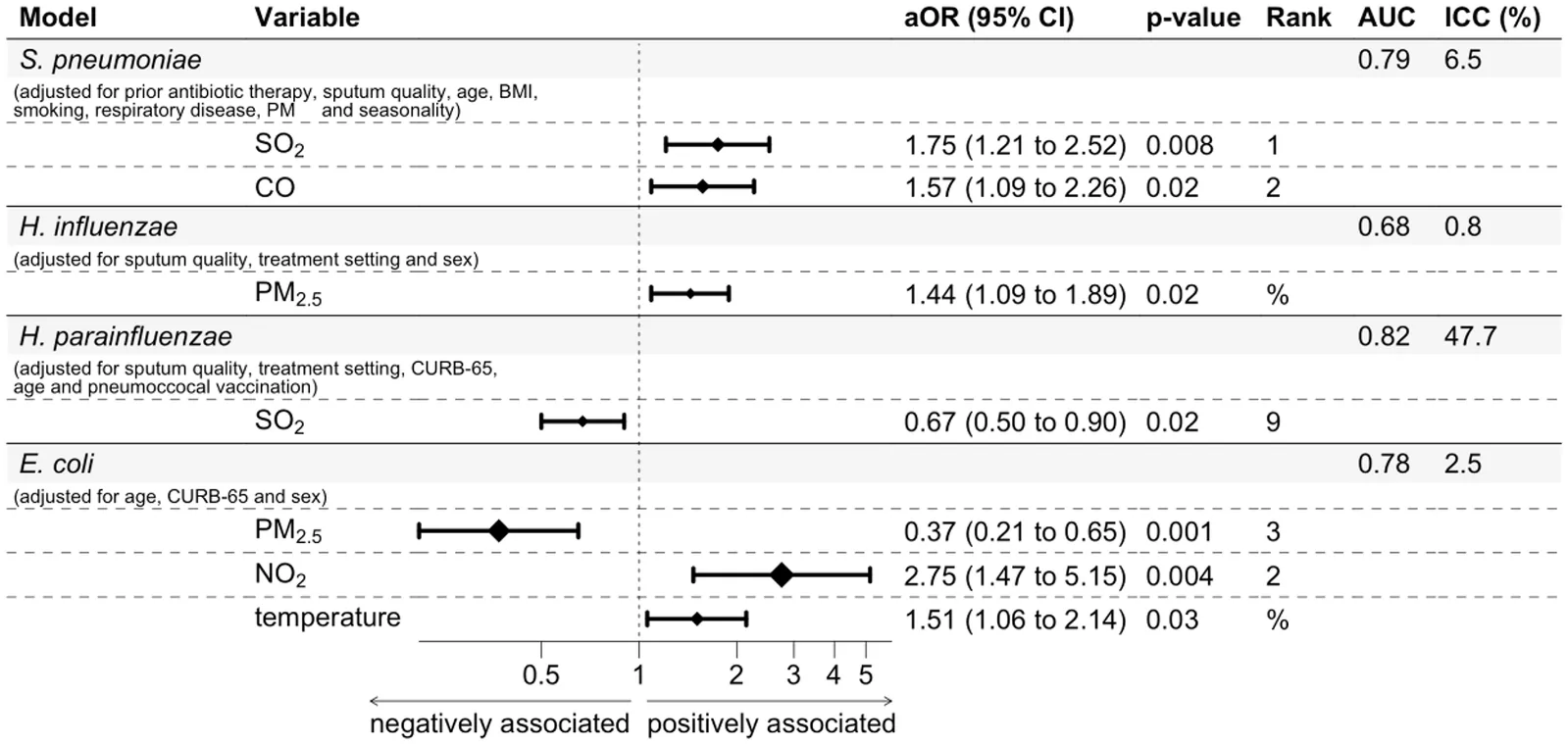
Associations of air pollution and meteorological variables with sputum pathogen spectrum. Forest plot displaying the effects of air pollution and confounding meteorological variables on outcomes. To improve clarity, only significant environmental variables are depicted. Other variables are listed below each model. *S. aureus* detection was not significantly influenced by environmental variables. aOR: Adjusted Odds-Ratio, equal to one standard deviation of change. Rank: Rank of importance as estimated by ensemble feature selection. AUC: Area under the curve. ICC: Intra-class correlation coefficient, representing variance in the model explained by centre-level heterogeneity. p-value: adjusted with Benjamin-Hochberg-procedure. %: variable was not chosen by feature selection. CO, NO_2_, and SO_2_ represent total-column quantities and are not equivalent to ground-level concentrations; PM_2.5_ is reported as a surface concentration variable

In the analysis of sputum pathogen spectrum (see Fig. 3), detection of *S. pneumoniae* increased with higher exposure to SO_2_ (OR (Odds ratio) 1.75, *p* = 0.008) and CO (OR 1.57, *p* = 0.02).

For *H. influenzae*, increased detection was associated with elevated exposure to PM_2.5_ (OR 1.44, *p* = 0.02) while *H. parainfluenzae* detection was inversely associated with elevated SO_2_ levels before inclusion (OR 0.67, *p* = 0.02).

Lastly, *E. coli* detection occurred more frequently in patients who were exposed to elevated NO_2_ (OR 2.75, *p* = 0.004) and higher temperature (OR 1.51, *p* = 0.03), whereas increased PM_2.5_ exposure (OR 0.37, *p* = 0.001) was linked to reduced detection. No meaningful relations between environmental variables and *S. aureus* detection were observed.

In the exploratory analysis of *C. albicans*, detection rose with increasing exposure to NO_2_ (OR 1.9, *p* = 0.04) and humidity (OR 1.68, *p* = 0.002), whereas exposure to higher levels of SO_2_ substantially reduced detection (OR 0.09, *p* < 0.001).

**Fig. 4:**
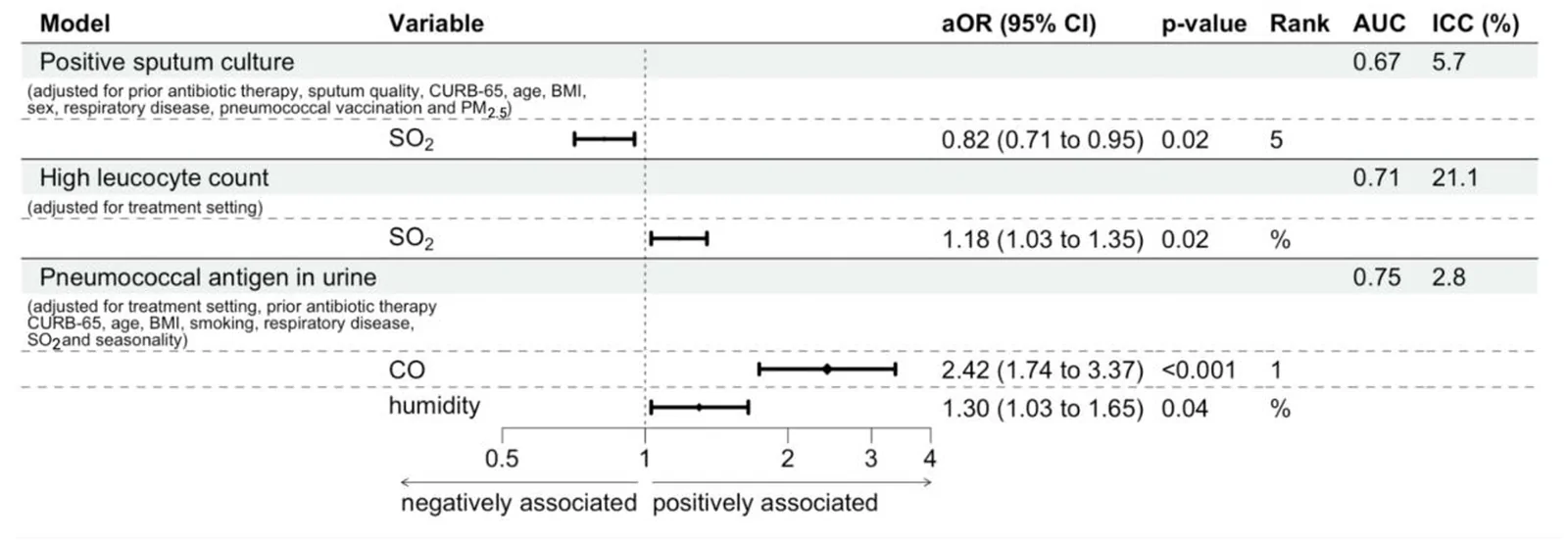
Associations of air pollution and meteorological variables with positive sputum culture and leucocyte cell counts and pneumococcal antigen in urine. Forest plot displaying the effects of air pollution and confounding meteorological variables on outcomes. To improve clarity, only significant environmental variables are depicted. Other variables are listed below each model. Epithelial cell count was not significantly influenced by environmental variables. aOR: adjusted Odds-Ratio, equal to one standard deviation of change. rank: rank of importance as estimated by ensemble feature selection. AUC: area under the curve. ICC: Intra-class correlation coefficient, representing variance in the model explained by centre-level heterogeneity. p-value: adjusted with Benjamin-Hochberg-procedure. CO and SO_2_ represent total-column quantities and are not equivalent to ground-level concentrations

Regarding sputum characteristics (Fig. 4), higher exposure to SO_2_ decreased the odds of obtaining a positive sputum culture (OR 0.82, *p* = 0.02) but increased the chances of detecting a high leucocyte count (OR 1.18, *p* = 0.02). No significant associations were found for epithelial cell counts.

Lastly, a positive pneumococcal antigen test in urine was associated with elevated CO exposure (OR 2.42, *p* < 0.001) and higher humidity (OR 1.3, *p* = 0.04).

Model performance showed moderate to high discriminative ability, with AUC values ranging from 0.67 in the sputum culture positivity model to 0.82 in the *H. parainfluenzae* model. Inter-centre heterogeneity varied considerably, with Intra-Class-Correlation (ICC) coefficients ranging from low (0.8%) for *H. influenzae* detection to high (47.7%) for *H. parainfluenzae* detection. Detailed model output is listed in the supplement (see Table S8a-j; supplement).

### Combined air pollution exposure reveals distinct pathogen detection patterns of *S. pneumoniae* and *E. coli*

**Fig. 5:**
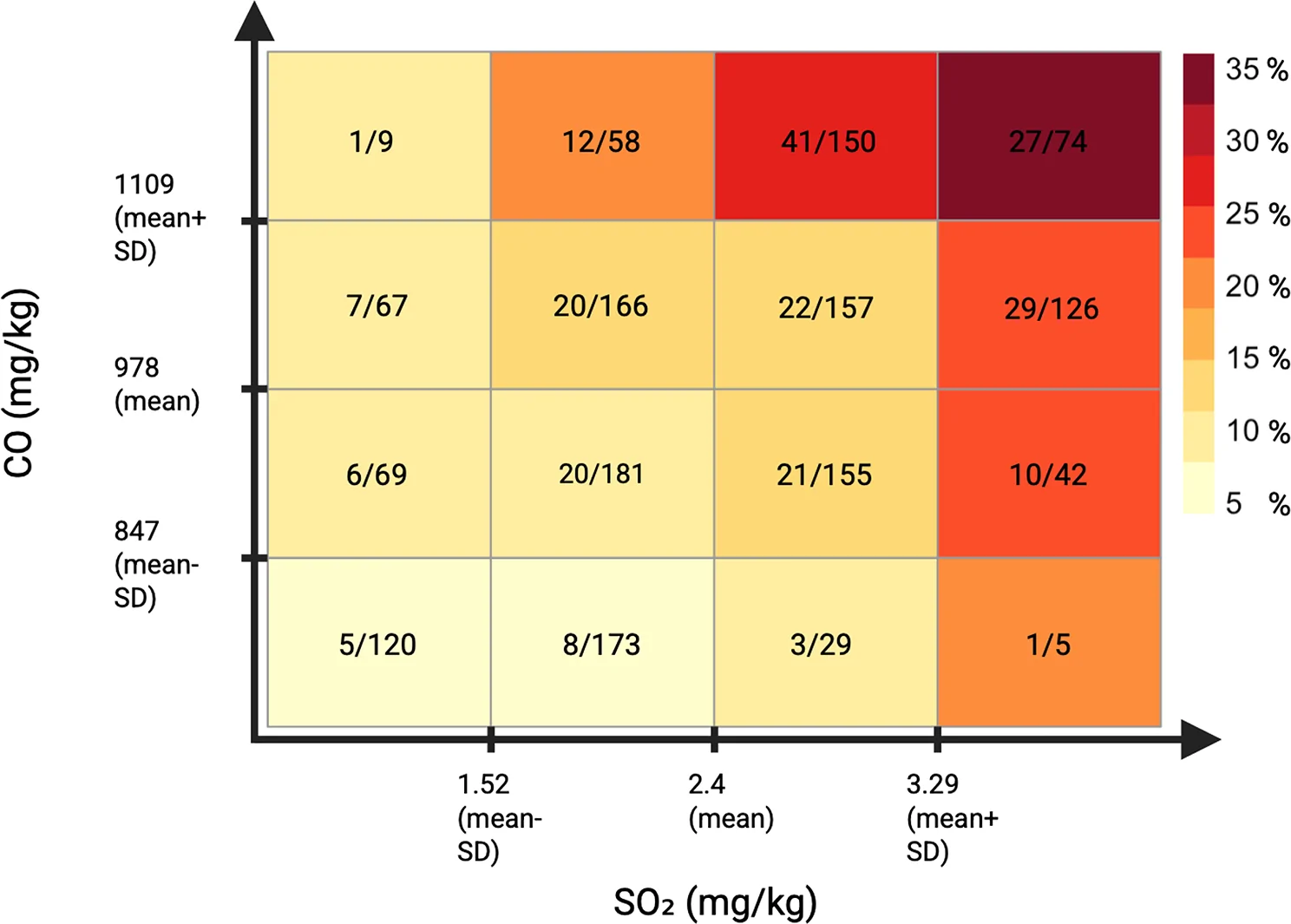
*S. pneumoniae* detection in relation to SO₂ and CO exposure. Heat map displaying *S. pneumoniae* detection in relation to CO and SO₂ exposure. Patients were divided into clusters depending on their CO and SO₂ exposure in regard to the mean, mean + 1 standard deviation and mean − 1 standard deviation of the cohort. The colour scale depicts *S. pneumoniae* detection proportion in percent per cluster with darker colours representing higher percentages. Absolute numbers are displayed in the middle of each cluster. CO and SO_2_ represent total-column quantities and are not equivalent to ground-level concentrations

**Fig. 6:**
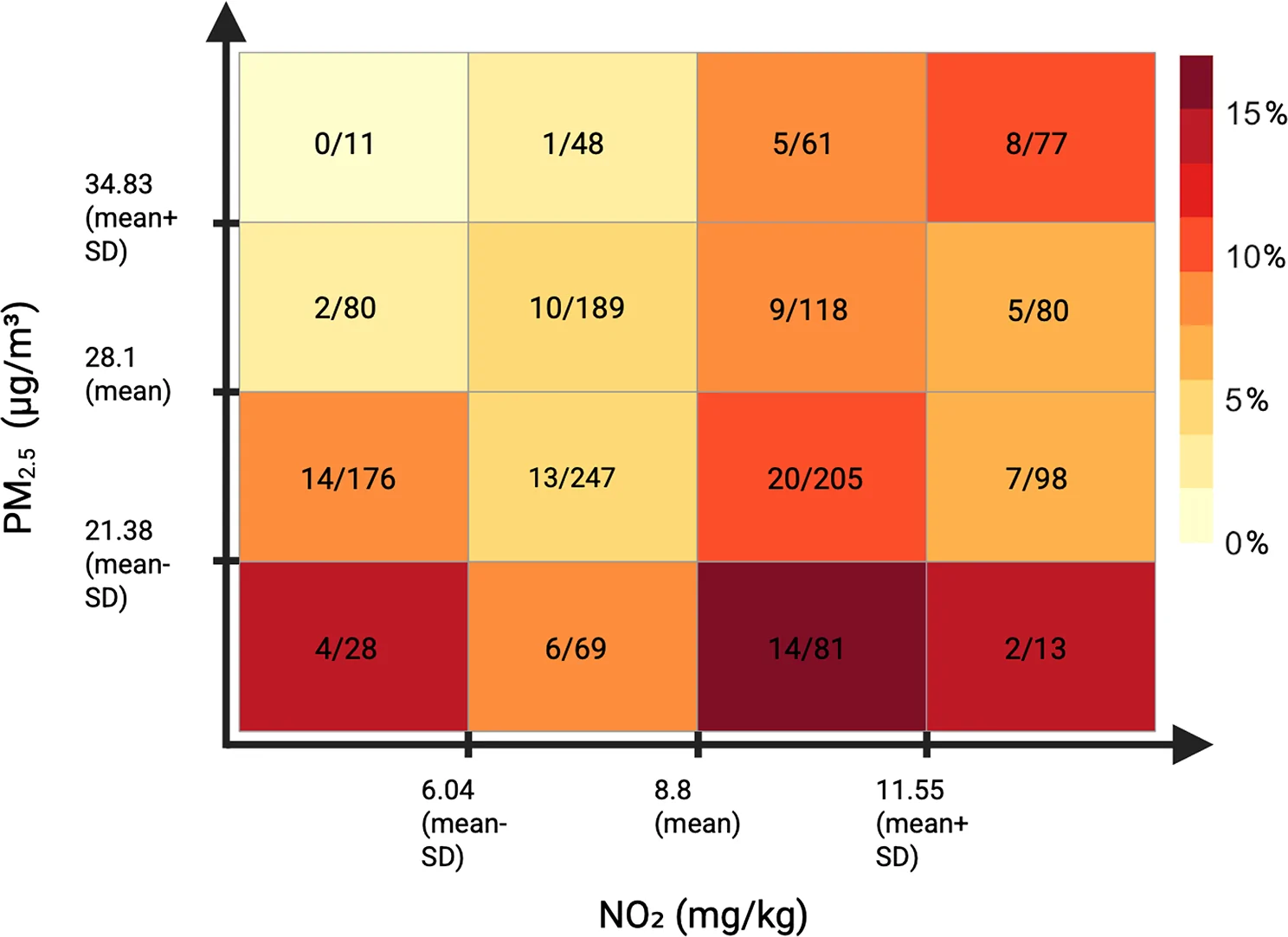
*E. coli* detection in relation to NO₂ and PM_2.5_ exposure. Heat map displaying *E. coli* detection in relation to PM_2.5_ and NO2 exposure. Patients were divided into clusters depending on their PM_2.5_ and NO₂ exposure in regard to the mean, mean + 1 standard deviation and mean − 1 standard deviation of the cohort. The colour scale depicts *E. coli* detection proportion in percent per cluster with darker colours representing higher percentages. Absolute numbers are displayed in the middle of each cluster. NO_2_ represents a total-column quantity and is not equivalent to a ground-level concentration. PM_2.5_ is reported as a surface concentration variable

Air pollution data can be applied to stratify patients into groups with differing pathogen detection proportions. Figures 5 and 6 display heat maps combining two air pollutants for each pathogen.

For *S. pneumoniae* (see Fig. 5), the highest detection proportion of 36% (compared with 15% in the total sample) was observed in the top-right cluster, representing patients whose exposure to both SO₂ and CO exceeded the mean plus one standard deviation (SD). When pollutants were considered individually, clusters with exposure above the mean plus one SD showed markedly lower detection proportions of 27% for SO₂ and 28% for CO.

Conversely, only 4.2% of patients in the bottom-left cluster, with exposure below mean minus one SD for both pollutants, tested positive for *S. pneumoniae*. Under single-pollutant stratification, the corresponding fringe clusters displayed detection proportions of 7.2% for SO₂ and 5.2% for CO exposure.

A similar pattern emerged for *E. coli* detection (see Fig. 6). The lowest detection proportions (0%, 2.1%, and 2.5%, compared with 7.6% overall) occurred in clusters characterized by high PM_2.5_ exposure combined with low NO₂ exposure, as well as in the adjacent clusters. When considered separately, patients with very low NO₂ exposure showed a detection proportion of 6.8%, while those with very high PM_2.5_ exposure showed a detection proportion of 7.1%.

The cluster with an opposite exposure profile in the bottom right corner and its left neighbour, showed detection proportions of 15% and 17%. Single-pollutant stratification again yielded less pronounced differences, with detection proportions of 14% for low PM_2.5_ alone and 8.2% for high NO_2_ exposure. As with *S. pneumoniae*, combining two exposure metrics reveals clearer gradients in *E. coli* detection proportion than relying on one pollutant at a time.

### Internal validation with ensemble feature selection

Feature selection and variable-importance analysis using the EFS approach largely supported the logistic regression findings. In the case of *S. pneumoniae* detection, SO_2_ and CO received ranks 1 and 2 and for *E. coli* detection NO_2_ and PM_2.5_ received ranks 2 and 3. Discrepancies were observed for models of *H. influenzae*, where PM_2.5_ was not chosen by EFS and *H. parainfluenzae*, where SO_2_ was chosen but ranked as low importance. The impact of SO_2_ on leucocyte cell counts was not confirmed by EFS. For epithelial cell counts, NO_2_ and air pressure were chosen as important variables in EFS but not included in the logistic regression models. A complete listing of the chosen variables is presented in the supplement (see Table S9a, b supplement). Bar plots displaying the composition of EFS scores are displayed in the supplement (see Figure S1a-j, supplement).

### Sensitivity and exploratory analysis

Sensitivity analyses using alternative exposure windows largely supported the direction and overall interpretation of the principal associations, although effect estimates and statistical precision varied across individual models and exposure windows (see Tables S10a-c, S11a-c, S12a-e, S13a-c, S14a-c, S15a-c, S16a-e, S17a-e; supplement).

The MICE analysis generally confirmed the direction and magnitude of the principal associations in the primary analyses. The pooled model estimates and the distribution of the imputed variables across the 20 imputed data sets and the corresponding values in the original dataset are presented in the supplement (see Tables S18a-b, S19a-b, S20a-b, S21a-b, S22a-b, S23a-b, S24a-b; supplement).

Ground-level air pollution measurements could be matched to 120 cases from the whole data set, including 90 cases from the analytical study population. Correlations between EAC4 total-column values and measured ground-level concentrations varied by pollutant and study site. Agreement was most consistent for NO_2_ and was also observed for CO at selected stations, whereas SO₂ and O₃ showed more heterogeneous and generally weaker correlations across study sites (see Table S25; supplement).

## Discussion

To our knowledge, this is the first study to investigate the association between air pollution and the pathogen spectrum of patients with CAP in a prospective, multicentre cohort. We report two main findings: First, strong associations with air pollution exposure were seen for *S. pneumoniae* and *E. coli* sputum detection. Based on these results, we demonstrated how combining two air pollution exposures may help stratify patients into high and low detection frequency clusters. These findings point out that air pollution exposure must not be regarded as a uniform actor but as a complex interplay of multiple factors. Secondly, air pollution was associated with sputum culture positivity and high leucocyte count, which may have methodological implications regarding the diagnostic process. Overall, these associations underscore the importance of considering air pollution exposure as a confounding factor when interpreting and analysing microbiological data within an epidemiological and clinical context.

It is important to note that this study examined associations between air pollution exposure and the pathogen spectrum among patients with an established diagnosis of CAP. As air pollution also affects the risk of developing CAP itself, and analysis was conditional on CAP diagnosis, our results do not allow inference on absolute pathogen prevalence but rather reflect relative differences in pathogen detection among patients with CAP.

The observed pathogen-specific associations may reflect a combination of impaired epithelial and innate immune defence, pollution-related changes in the respiratory microbiome, and direct effects of pollutants on microbial growth, biofilm formation, or virulence. These mechanisms are likely to differ between pollutants and organisms and cannot be distinguished in the present observational study.

Regarding *S. pneumoniae* sputum detection, a correlation was found for higher CO and SO_2_ exposure, while CO exposure was also associated with a higher proportion of positive pneumococcal antigen tests in urine. In the literature, associations of SO_2_ and CO and pneumococcal infection have been described, with one study also listing temperature and NO as important factors [29, 51]. However, there are notable differences, as these studies either did not account for clinical confounders, used singular pollutant models, or did not compare multiple pathogens. It is important to note that sputum pathogen detection of *S. pneumoniae* or other pathogens does not necessarily indicate active infection and may reflect colonisation or contamination from the upper airways. However, concordant associations were observed for both sputum pathogen detection and pneumococcal antigen positivity in urine. As pneumococcal antigen positivity is associated with disease severity and adverse clinical outcomes, this supports the clinical relevance of our findings and suggests that the observed associations for *S. pneumoniae* are not restricted to contamination or colonisation alone [52, 53]. Additionally, Saukkoriipi et al. recently found that all sputum samples regardless of quality contributed to the diagnosis of CAP [54]. No association of PM_2.5_ with *S. pneumoniae* detection compared to other pathogens in the context of CAP was found in our study, which is surprising as PM_2.5_ exposure seems to increase the overall risk of developing CAP in both clinical and experimental studies and is associated with increased pneumococcal carriage in the nasopharynx [6, 7, 55, 56].

Surprisingly, *E. coli* detection was negatively influenced by PM_2.5_ and positively influenced by NO_2_ exposure, illustrating the complex, multifaceted influence of pollutants on the pathogen spectrum. A potential mechanism behind the association of *E. coli* and NO₂ may be that, contrary to other bacteria, *E. coli* is well-adapted to nitrogen compounds, which develop when NO₂ reacts with the moisture in the respiratory tract, and can metabolize these substrates [57, 58]. Contrary, an experimental PM_2.5_ exposure study has demonstrated a reduction in *E. coli* growth rate and subsequent adaptation which is in line with our results that show an inverse relationship between PM_2.5_ exposure and *E. coli* detection [59]. In light of the poorer outcome of CAP caused by *E. coli* or other *Enterobacteriaceae*, which may produce extended-spectrum ß-lactamase (ESBL) complicating antibiotic therapy, these findings may be relevant for understanding environmental drivers of pathogen distribution and for informing epidemiological surveillance and antimicrobial stewardship strategies in regions with high air pollution exposure [60–62]. In addition, air pollution itself has emerged as a potential driver of antimicrobial resistance (AMR), suggesting that air pollution may add to existing resistance burdens and underscoring the need for further studies investigating the complex interplay between air pollution, pathogen spectrum and AMR in infectious diseases [63].

The modest relation between *H. influenzae* detection and PM_2.5_ exposure in our study aligns with previous reports identifying PM_2.5_ as an independent predictor of CAP caused by *H. influenzae* [30]. Moreover, an increased growth rate of *H. influenzae* has been demonstrated after exposure to atmospheric pollutants without specifying individual pollutants [64]. In our exploratory analysis, *C. albicans* detection decreased by more than 90% for an increase in SO_2_ equal to one SD, which is consistent with experimental evidence indicating that sulphur inhibits yeast growth through the induction of disulfide stress [65]. *C. albicans* is not considered a causative pathogen of CAP, and its detection in respiratory samples generally reflects airway colonisation rather than infection. Although experimental studies have suggested that *Candida* species may interact with bacterial pathogens and modulate local immune-responses, the clinical significance of these interactions in CAP remains uncertain [66].

In our study, elevated exposure to SO_2_ was associated with more frequent detection of high leucocyte counts and lower odds of a positive sputum culture. We are not aware of any studies that examined different degrees of inflammation related to air pollution in CAP. In patients with COPD, a study by Dragonieri et al. showed increased neutrophil counts in patients living in urban vs. rural areas [67]. We hypothesize that increased inflammation associated with SO_2_ exposure may alter the relationship between bacterial burden and clinical disease, resulting in lower sputum culture positivity despite clinically relevant infection. These findings have methodological implications: air pollution exposure could influence the interpretation of sputum diagnostics in both epidemiological research and routine care, independently of host characteristics.

We did not observe any significant associations between O_3_ and BC exposure and pathogen detection in our analysis. Although O_3_ exposure has been linked to an increased overall risk of hospitalization with CAP, experimental studies have demonstrated antimicrobial effects of O_3_ across a broad range of pathogens, which may complicate the detection of relative differences between individual pathogens in clinical studies [68–70]. Similarly, experimental work has shown that BC can affect *S. aureus* and *S. pneumoniae* biofilm formation and colonisation, although effects on other bacteria have not been evaluated [71].

The EFS approach largely confirmed variable selection and relevance. We observed some discrepancies which may have occurred due to non-linear trends and interaction effects that our modelling approach did not capture. Additionally, centre-specific differences were not captured in the EFS approach. Sensitivity analyses on different time frames and imputation generally supported the principal findings of the primary analysis.

The exploratory validation analysis supported the use of EAC4 columnar values as relative indicators of regional pollutant variability, but not as direct estimates of absolute ground-level exposure. In line with the pollutant- and site-dependent correlations observed in our validation analysis, associations involving SO₂ and O₃ should be interpreted with particular caution. These associations may reflect broader pollution mixtures or regional atmospheric conditions rather than isolated effects of absolute ground-level concentrations. The observed uncertainties arise from (i) variations in vertical concentrations along the air column in between time steps, (ii) the coarse spatial resolution and consequent averaging of the EAC4 data compared with the point measurements at the stations, which strongly averages out and thus smooths measured extreme values, and (iii) the different temporal integration of measurements and EAC4 data. These uncertainties are inherent to the EAC4 dataset and therefore cannot be altered.

Environmental exposures were assigned according to the recruiting centre and surrounding municipalities because residential addresses were unavailable. Although healthcare accessibility and travel times to healthcare services are generally favourable across Germany [72], individual patients may have resided outside the exposure area represented by the recruiting centre. Spatial exposure misclassification therefore cannot be excluded and may have attenuated the observed associations.

Residual confounding by area-level characteristics, including urbanicity, socioeconomic conditions, and green space, cannot be excluded. Future studies should therefore incorporate geocoded residential and neighbourhood-level data.

As this study was conducted in a high-income country, the findings may have limited transferability to LMICs, where environmental exposures, pathogen spectra, and healthcare conditions may differ and pollution-related health burdens may be greater.

This study has several strengths. First, we used a large and well-characterized prospective multicentre study of CAP, which we chose due to the substantial number of available sputa. Second, we simultaneously analysed associations between multiple pollutants and pathogens. Third, the robustness of results was supported by using both regression and machine learning techniques.

As explained above, a major limitation of this study is the combined use of spatial and vertical proxies, which may have resulted in exposure misclassification and attenuated the observed associations, although the direction and magnitude of any resulting bias remain unknown. While we believe that the approach of using median absolute values is appropriate for this study design, we acknowledge that many studies used more complex statistical methods, such as distributed lag non-linear models (DLNM) and that in the analysis of meteorological conditions, changes may matter more than absolute values [73, 74]. From a clinical perspective, high quality microbiological and AMR data were not available and absolute bacterial quantities were not available.

## Conclusions

We identified a distinct pathogen spectrum associated with air pollution exposure in patients with CAP. These findings suggest that air pollution may influence the interpretation of microbiological data in an epidemiological and clinical context and highlight the importance of considering air pollution exposure as a confounding factor. Validation in cohorts with high-quality environmental, microbiological and AMR data is needed, alongside further mechanistic studies to clarify the biological pathways underlying these associations.

## Supplementary Information

Below is the link to the electronic supplementary material.

Supplementary Material 1

## Data Availability

Data and biomaterials from the CAPNETZ study can be requested by a formal application and will be decided upon by the board of the CAPNETZ STIFTUNG (https://capnetz.de/en/project-application/).

## Abbreviations

AIC: Akaike’s Information Criterion
AMR: Antimicrobial resistance
AUC: Area under the curve
BC: Black carbon
BMI: Body mass index
CAP: Community-acquired pneumonia
CAPNETZ: Competence Network for Community-Acquired Pneumonia
CO: Carbon monoxide
COPD: Chronic obstructive pulmonary disease
CURB-65: Confusion, Urea, Respiratory rate, Blood pressure, age ≥ 65 years score
DLNM: Distributed lag non-linear model
DRKS: German Clinical Trials Register
DWD: German Weather Service (Deutscher Wetterdienst)
EAC4: Copernicus Atmosphere Monitoring Service global reanalysis
EFS: Ensemble Feature Selection
ESBL: Extended-spectrum β-lactamase
FOV: Field of view
ICC: Intra-class correlation coefficient
LMICs: Low- and middle-income countries
NO_2_: Nitrogen dioxide
O_3_: Ozone
OR: Odds ratio
PM_2.5_: Fine particulate matter with aerodynamic diameter ≤ 2.5 μm
ROC: Receiver operating characteristic
SD: Standard deviation
SO_2_: Sulphur dioxide
SRTM: Shuttle Radar Topography Mission
