# Supplementary material for: Air pollution is associated with a distinct pathogen spectrum in community-acquired pneumonia: a prospective multicentre cohort study
Source: Pneumonia (Nathan). 2026 Nov 5;18:25. doi: 10.1186/s41479-026-00212-3 (PMC13737847; doi:10.1186/s41479-026-00212-3)
Supplement: Supplementary file 1 [file 41479_2026_212_MOESM1_ESM.docx]

**Supplement**

**Note on air pollution variables:**

CO, NO_2_, O_3_ and SO_2_ represent total-column quantities and are not equivalent to ground-level concentrations; PM_2.5_ is reported as a surface concentration variable, while black carbon is represented by dimensionless aerosol optical depth.

**Table S1: Table of hospitals and outpatient clinics included in the study as well as considered period**

| Hospitals/ Outpatient Clinics | Considered Period | Cases^a^ | Coordinates | Altitude |
| --- | --- | --- | --- | --- |
| Aachen (Uniklinik RHTW) | 2008-2016 | 54 | 50° N, 6° E | 230 m |
| Bad Arolsen (Krankenhaus) | 2011-2016 | 0 | 51° N, 9° E | 290 m |
| Berlin-Charité (Universitätsmedizin Berlin) | 2005-2017 | 278 | 52° N, 13° E | 35 m |
| Berlin-Heckeshorn (Lungenklinik) | 2003-2017 | 912 | 52° N, 13° E | 49 m |
| Berlin-Neukölln (Vivantes Klinikum Neukölln) | 2009-2016 | 16 | 52° N, 13° E | 58 m |
| Bochum (Augusta Kliniken) | 2003-2017 | 394 | 51° N, 7° E | 103 m |
| Cottbus (Carl-Thiem Klinikum) | 2009-2017 | 253 | 51° N, 14° E | 91 m |
| Dresden (Universitätsklinikum Carl Gustav Carus)^b^ | 2013-2016 | 1 | 51° N, 13° E | 113 m |
| Hannover (Lungenpraxis Kock) | 2005-2016 | 221 | 52° N, 9° E | 79 m |
| Heidelberg (Thoraxklinik) | 2016-2017 | 0 | 49° N, 8° E | 127 m |
| Jena (Universitätsklinikum Jena) | 2011-2016 | 0 | 50° N, 11° E | 195 m |
| Köln (Universitätsklinikum) | 2003-2004 | 51 | 51° N, 7° E | 56 m |
| Leipzig (Robert Koch-Klinik)^b^ | 2006-2009 | 39 | 51° N, 12° E | 128 m |
| Lübeck (Infektiologie Universitätsklinikum) | 2003-2016 | 453 | 53° N, 10° E | 28 m |
| Lüdenscheid (Lüdenscheid Klinikum) | 2003-2008 | 61 | 51° N, 7° E | 452 m |
| Würzburg Mitte (Missioklinik)^b^ | 2003-2004 | 27 | 49° N, 10° E | 234 m |
| Paderborn (Brüderkrankenhaus St. Josef)^b^ | 2009-2016 | 39 | 51° N, 8° E | 174 m |
| Regensburg (Klinikum der Universität Regensburg)^b^ | 2010-2011 | 2 | 48° N, 12° E | 427 m |
| Rostock (Institut für Klinische Pharmakologie)^b^ | 2008-2010 | 8 | 54° N, 12° E | 21 m |
| Rotenburg (Agaplesion Diakonieklinikum) | 2003-2017 | 382 | 53° N, 9° E | 24 m |
| Ulm (Universitätsklinikum Ulm) | 2004-2015 | 725 | 48° N, 9° E | 615 m |
| Magdeburg (Universitätsklinikum Magdeburg) | 2003-2005 | 98 | 52° N, 11° E | 56 m |

^a^out of 4014 included patients

^b^summarized as one centre for analysis of random effects

| Table S2: Meteorological and air quality conditions included in the study | | | |
| --- | --- | --- | --- |
| Condition | **Unit** | **Description** | **Data source** |
| Meteorology | | |  |
| Air pressure | hPa | Average daily air pressure | DWD |
| Relative Humidity | % | Daily average relative humidity | DWD |
| Maximum temperature | °C | Daily average, minimum, and maximum temperature | DWD |
| Air quality | | |  |
| Maximum total column* CO | mg/kg | Daily maximum total column of CO | ECMWF |
| Total column* NO_2_ | mg/kg | Daily maximum total column of NO_2_ | ECMWF |
| Total column* O_3_ | g/m^2^ | Daily maximum total column of O_3_ | ECMWF |
| Total column* SO_2_ | mg/kg | Daily maximum total column of SO_2_ | ECMWF |
| PM 2.5 concentrations | μg/m^3^ | Daily maximum of particulate matter < 2.5 µm (aerosol) | ECMWF |
| Black carbon** | dimensionless | Daily maximum aerosol optical depth at 550 nm for black carbon | ECMWF |

* Total column refers to the total amount of the selected variable in a column of air extending from the surface of the Earth to the top of the atmosphere.

**The aerosol optical depth measures the amount of light lost due to the presence of aerosols on a vertical path through the atmosphere.

**Table S3: Exploratory univariable associations between meteorological and air pollution**

**variables and 7-day mortality in the overall CAPNETZ cohort with available mortality data,**

**adjusted for season**

| Characteristic | N | OR | 95% CI | p-value |
| --- | --- | --- | --- | --- |
| Temperature | | | | |
| Temperature, day 0 | 9,624 | 1.10 | 0.90, 1.35 | 0.36 |
| Temperature, day 1 | 9,624 | 1.21 | 0.99, 1.49 | 0.07 |
| Temperature, day 2 | 9,624 | **1.34** | **1.10, 1.65** | **0.004** |
| Temperature, day 3 | 9,624 | **1.34** | **1.10, 1.65** | **0.004** |
| Temperature, day 4 | 9,624 | **1.27** | **1.04, 1.56** | **0.02** |
| Temperature, day 5 | 9,624 | **1.31** | **1.07, 1.60** | **0.01** |
| Temperature, day 6 | 9,624 | 1.17 | 0.95, 1.44 | 0.13 |
| Temperature, day 7 | 9,624 | 1.13 | 0.93, 1.39 | 0.22 |
| Temperature, day 8 | 9,624 | 1.21 | 0.99, 1.48 | 0.06 |
| Temperature, day 9 | 9,624 | **1.25** | **1.03, 1.53** | **0.03** |
| Temperature, day 10 | 9,624 | 1.20 | 0.98, 1.46 | 0.07 |
| Temperature, day 11 | 9,624 | 1.17 | 0.96, 1.43 | 0.11 |
| Temperature, day 12 | 9,624 | 1.13 | 0.92, 1.37 | 0.24 |
| Temperature, day 13 | 9,624 | 1.13 | 0.93, 1.38 | 0.22 |
| Temperature, day 14 | 9,624 | 1.20 | 0.99, 1.46 | 0.07 |
| Temperature, day 15 | 9,624 | 1.20 | 0.99, 1.46 | 0.06 |
| Temperature, day 16 | 9,624 | 1.09 | 0.89, 1.32 | 0.41 |
| Temperature, day 17 | 9,624 | 1.07 | 0.88, 1.31 | 0.47 |
| Temperature, day 18 | 9,624 | 1.12 | 0.92, 1.37 | 0.24 |
| Temperature, day 19 | 9,624 | 1.09 | 0.90, 1.32 | 0.39 |
| Temperature, day 20 | 9,624 | 1.08 | 0.89, 1.31 | 0.42 |
| Temperature, day 21 | 9,624 | 1.10 | 0.91, 1.33 | 0.33 |
| Temperature, day 22 | 9,624 | 1.14 | 0.94, 1.38 | 0.19 |
| Temperature, day 23 | 9,624 | 1.16 | 0.95, 1.41 | 0.14 |
| Temperature, day 24 | 9,624 | 1.19 | 0.98, 1.45 | 0.07 |
| Temperature, day 25 | 9,624 | 1.19 | 0.98, 1.44 | 0.08 |
| Temperature, day 26 | 9,624 | **1.24** | **1.02, 1.50** | **0.03** |
| Temperature, day 27 | 9,624 | **1.33** | **1.10, 1.61** | **0.003** |
| Temperature, day 28 | 9,624 | **1.28** | **1.06, 1.55** | **0.01** |
| Temperature, day 29 | 9,624 | **1.25** | **1.03, 1.52** | **0.02** |
| Temperature, day 30 | 9,624 | **1.32** | **1.09, 1.60** | **0.005** |
| Temperature, day 31 | 9,624 | 1.19 | 0.98, 1.45 | 0.08 |
| Humidity | | | | |
| Humidity, day 0 | 9,624 | 0.88 | 0.77, 1.03 | 0.1 |
| Humidity, day 1 | 9,624 | 0.97 | 0.84, 1.13 | 0.67 |
| Humidity, day 2 | 9,624 | 0.90 | 0.78, 1.04 | 0.14 |
| Humidity, day 3 | 9,624 | **0.80** | **0.70, 0.92** | **0.001** |
| Humidity, day 4 | 9,624 | **0.82** | **0.72, 0.95** | **0.007** |
| Humidity, day 5 | 9,624 | **0.78** | **0.68, 0.90** | **<0.001** |
| Humidity, day 6 | 9,624 | **0.80** | **0.69, 0.92** | **0.002** |
| Humidity, day 7 | 9,624 | 0.94 | 0.81, 1.09 | 0.42 |
| Humidity, day 8 | 9,624 | 0.90 | 0.78, 1.04 | 0.16 |
| Humidity, day 9 | 9,624 | 0.87 | 0.76, 1.01 | 0.06 |
| Humidity, day 10 | 9,624 | **0.85** | **0.74, 0.97** | **0.02** |
| Humidity, day 11 | 9,624 | **0.85** | **0.74, 0.98** | **0.02** |
| Humidity, day 12 | 9,624 | **0.82** | **0.72, 0.94** | **0.005** |
| Humidity, day 13 | 9,624 | **0.79** | **0.69, 0.90** | **<0.001** |
| Humidity, day 14 | 9,624 | **0.79** | **0.69, 0.91** | **<0.001** |
| Humidity, day 15 | 9,624 | **0.81** | **0.71, 0.93** | **0.003** |
| Humidity, day 16 | 9,624 | **0.85** | **0.74, 0.98** | **0.02** |
| Humidity, day 17 | 9,624 | **0.87** | **0.75, 1.00** | **0.04** |
| Humidity, day 18 | 9,624 | **0.80** | **0.70, 0.92** | **0.002** |
| Humidity, day 19 | 9,624 | **0.80** | **0.70, 0.91** | **0.001** |
| Humidity, day 20 | 9,624 | **0.84** | **0.73, 0.97** | **0.01** |
| Humidity, day 21 | 9,624 | **0.80** | **0.70, 0.92** | **0.001** |
| Humidity, day 22 | 9,624 | **0.86** | **0.75, 1.00** | **0.04** |
| Humidity, day 23 | 9,624 | **0.81** | **0.71, 0.93** | **0.002** |
| Humidity, day 24 | 9,624 | **0.83** | **0.72, 0.95** | **0.006** |
| Humidity, day 25 | 9,624 | **0.81** | **0.71, 0.93** | **0.003** |
| Humidity, day 26 | 9,624 | **0.79** | **0.69, 0.90** | **<0.001** |
| Humidity, day 27 | 9,624 | **0.79** | **0.69, 0.91** | **<0.001** |
| Humidity, day 28 | 9,624 | **0.82** | **0.72, 0.94** | **0.004** |
| Humidity, day 29 | 9,624 | **0.84** | **0.73, 0.97** | **0.01** |
| Humidity, day 30 | 9,624 | **0.84** | **0.73, 0.96** | **0.01** |
| Humidity, day 31 | 9,624 | **0.87** | **0.76, 1.00** | **0.04** |
| Air pressure | | | | |
| Air pressure, day 0 | 9,624 | 0.96 | 0.84, 1.09 | 0.51 |
| Air pressure, day 1 | 9,624 | 0.94 | 0.83, 1.07 | 0.32 |
| Air pressure, day 2 | 9,624 | 0.92 | 0.81, 1.04 | 0.18 |
| Air pressure, day 3 | 9,624 | 0.95 | 0.83, 1.08 | 0.42 |
| Air pressure, day 4 | 9,624 | 0.96 | 0.85, 1.10 | 0.59 |
| Air pressure, day 5 | 9,624 | 0.96 | 0.85, 1.10 | 0.58 |
| Air pressure, day 6 | 9,624 | 0.97 | 0.85, 1.10 | 0.62 |
| Air pressure, day 7 | 9,624 | 0.94 | 0.83, 1.07 | 0.36 |
| Air pressure, day 8 | 9,624 | 0.93 | 0.82, 1.06 | 0.25 |
| Air pressure, day 9 | 9,624 | 0.95 | 0.83, 1.08 | 0.4 |
| Air pressure, day 10 | 9,624 | 0.98 | 0.86, 1.11 | 0.71 |
| Air pressure, day 11 | 9,624 | 0.97 | 0.85, 1.11 | 0.64 |
| Air pressure, day 12 | 9,624 | 0.95 | 0.84, 1.08 | 0.43 |
| Air pressure, day 13 | 9,624 | 0.95 | 0.84, 1.08 | 0.45 |
| Air pressure, day 14 | 9,624 | 0.97 | 0.85, 1.10 | 0.61 |
| Air pressure, day 15 | 9,624 | 0.97 | 0.85, 1.11 | 0.66 |
| Air pressure, day 16 | 9,624 | 0.96 | 0.84, 1.09 | 0.53 |
| Air pressure, day 17 | 9,624 | 0.95 | 0.84, 1.08 | 0.42 |
| Air pressure, day 18 | 9,624 | 0.90 | 0.79, 1.02 | 0.1 |
| Air pressure, day 19 | 9,624 | 0.92 | 0.81, 1.04 | 0.19 |
| Air pressure, day 20 | 9,624 | 0.92 | 0.82, 1.05 | 0.23 |
| Air pressure, day 21 | 9,624 | 0.94 | 0.83, 1.07 | 0.32 |
| Air pressure, day 22 | 9,624 | 0.92 | 0.81, 1.05 | 0.2 |
| Air pressure, day 23 | 9,624 | 0.92 | 0.82, 1.05 | 0.23 |
| Air pressure, day 24 | 9,624 | 0.93 | 0.82, 1.06 | 0.25 |
| Air pressure, day 25 | 9,624 | 0.96 | 0.84, 1.09 | 0.49 |
| Air pressure, day 26 | 9,624 | 0.95 | 0.84, 1.09 | 0.47 |
| Air pressure, day 27 | 9,624 | 0.97 | 0.85, 1.10 | 0.6 |
| Air pressure, day 28 | 9,624 | 0.96 | 0.84, 1.09 | 0.52 |
| Air pressure, day 29 | 9,624 | 0.93 | 0.82, 1.05 | 0.24 |
| Air pressure, day 30 | 9,624 | 0.93 | 0.82, 1.06 | 0.25 |
| Air pressure, day 31 | 9,624 | 0.96 | 0.85, 1.10 | 0.56 |
| PM_2.5_ | | | | |
| PM_2.5_, day 0 | 9,624 | **1.37** | **1.24, 1.52** | **<0.001** |
| PM_2.5_, day 1 | 9,624 | **1.35** | **1.21, 1.49** | **<0.001** |
| PM_2.5_, day 2 | 9,624 | **1.38** | **1.25, 1.53** | **<0.001** |
| PM_2.5_, day 3 | 9,624 | **1.37** | **1.23, 1.51** | **<0.001** |
| PM_2.5_, day 4 | 9,624 | **1.32** | **1.18, 1.47** | **<0.001** |
| PM_2.5_, day 5 | 9,624 | **1.32** | **1.19, 1.47** | **<0.001** |
| PM_2.5_, day 6 | 9,624 | **1.32** | **1.18, 1.46** | **<0.001** |
| PM_2.5_, day 7 | 9,624 | **1.33** | **1.20, 1.48** | **<0.001** |
| PM_2.5_, day 8 | 9,624 | **1.36** | **1.22, 1.50** | **<0.001** |
| PM_2.5_, day 9 | 9,624 | **1.36** | **1.23, 1.51** | **<0.001** |
| PM_2.5_, day 10 | 9,624 | **1.39** | **1.25, 1.54** | **<0.001** |
| PM_2.5_, day 11 | 9,624 | **1.39** | **1.25, 1.54** | **<0.001** |
| PM_2.5_, day 12 | 9,624 | **1.39** | **1.25, 1.54** | **<0.001** |
| PM_2.5_, day 13 | 9,624 | **1.31** | **1.17, 1.46** | **<0.001** |
| PM_2.5_, day 14 | 9,624 | **1.35** | **1.21, 1.50** | **<0.001** |
| PM_2.5_, day 15 | 9,624 | **1.46** | **1.32, 1.61** | **<0.001** |
| PM_2.5_, day 16 | 9,624 | **1.39** | **1.25, 1.53** | **<0.001** |
| PM_2.5_, day 17 | 9,624 | **1.34** | **1.21, 1.49** | **<0.001** |
| PM_2.5_, day 18 | 9,624 | **1.37** | **1.23, 1.52** | **<0.001** |
| PM_2.5_, day 19 | 9,624 | **1.26** | **1.12, 1.40** | **<0.001** |
| PM_2.5_, day 20 | 9,624 | **1.24** | **1.11, 1.39** | **<0.001** |
| PM_2.5_, day 21 | 9,624 | **1.25** | **1.11, 1.39** | **<0.001** |
| PM_2.5_, day 22 | 9,624 | **1.28** | **1.15, 1.42** | **<0.001** |
| PM_2.5_, day 23 | 9,624 | **1.29** | **1.15, 1.43** | **<0.001** |
| PM_2.5_, day 24 | 9,624 | **1.33** | **1.19, 1.47** | **<0.001** |
| PM_2.5_, day 25 | 9,624 | **1.35** | **1.21, 1.49** | **<0.001** |
| PM_2.5_, day 26 | 9,624 | **1.36** | **1.22, 1.50** | **<0.001** |
| PM_2.5_, day 27 | 9,624 | **1.39** | **1.26, 1.54** | **<0.001** |
| PM_2.5_, day 28 | 9,624 | **1.35** | **1.21, 1.49** | **<0.001** |
| PM_2.5_, day 29 | 9,624 | **1.39** | **1.25, 1.53** | **<0.001** |
| PM_2.5_, day 30 | 9,624 | **1.33** | **1.20, 1.47** | **<0.001** |
| PM_2.5_, day 31 | 9,624 | **1.29** | **1.16, 1.42** | **<0.001** |
| SO₂ | | | | |
| SO₂, day 0 | 9,624 | **1.32** | **1.17, 1.48** | **<0.001** |
| SO₂, day 1 | 9,624 | **1.32** | **1.17, 1.48** | **<0.001** |
| SO₂, day 2 | 9,624 | **1.26** | **1.12, 1.41** | **<0.001** |
| SO₂, day 3 | 9,624 | **1.26** | **1.12, 1.41** | **<0.001** |
| SO₂, day 4 | 9,624 | **1.23** | **1.09, 1.39** | **<0.001** |
| SO₂, day 5 | 9,624 | **1.24** | **1.10, 1.39** | **<0.001** |
| SO₂, day 6 | 9,624 | **1.20** | **1.06, 1.35** | **0.003** |
| SO₂, day 7 | 9,624 | **1.32** | **1.17, 1.48** | **<0.001** |
| SO₂, day 8 | 9,624 | **1.35** | **1.21, 1.50** | **<0.001** |
| SO₂, day 9 | 9,624 | **1.35** | **1.21, 1.51** | **<0.001** |
| SO₂, day 10 | 9,624 | **1.26** | **1.12, 1.41** | **<0.001** |
| SO₂, day 11 | 9,624 | **1.24** | **1.10, 1.39** | **<0.001** |
| SO₂, day 12 | 9,624 | **1.26** | **1.11, 1.41** | **<0.001** |
| SO₂, day 13 | 9,624 | **1.24** | **1.10, 1.39** | **<0.001** |
| SO₂, day 14 | 9,624 | **1.30** | **1.15, 1.45** | **<0.001** |
| SO₂, day 15 | 9,624 | **1.29** | **1.15, 1.45** | **<0.001** |
| SO₂, day 16 | 9,624 | **1.29** | **1.15, 1.45** | **<0.001** |
| SO₂, day 17 | 9,624 | **1.29** | **1.15, 1.44** | **<0.001** |
| SO₂, day 18 | 9,624 | **1.28** | **1.14, 1.43** | **<0.001** |
| SO₂, day 19 | 9,624 | **1.28** | **1.14, 1.43** | **<0.001** |
| SO₂, day 20 | 9,624 | **1.32** | **1.18, 1.47** | **<0.001** |
| SO₂, day 21 | 9,624 | **1.32** | **1.18, 1.48** | **<0.001** |
| SO₂, day 22 | 9,624 | **1.35** | **1.21, 1.51** | **<0.001** |
| SO₂, day 23 | 9,624 | **1.34** | **1.20, 1.50** | **<0.001** |
| SO₂, day 24 | 9,624 | **1.41** | **1.25, 1.57** | **<0.001** |
| SO₂, day 25 | 9,624 | **1.33** | **1.18, 1.49** | **<0.001** |
| SO₂, day 26 | 9,624 | **1.33** | **1.18, 1.48** | **<0.001** |
| SO₂, day 27 | 9,624 | **1.38** | **1.23, 1.54** | **<0.001** |
| SO₂, day 28 | 9,624 | **1.35** | **1.20, 1.51** | **<0.001** |
| SO₂, day 29 | 9,624 | **1.31** | **1.17, 1.46** | **<0.001** |
| SO₂, day 30 | 9,624 | **1.27** | **1.12, 1.42** | **<0.001** |
| SO₂, day 31 | 9,624 | **1.31** | **1.16, 1.46** | **<0.001** |
| CO | | | | |
| CO, day 0 | 9,624 | **1.44** | **1.20, 1.73** | **<0.001** |
| CO, day 1 | 9,624 | **1.39** | **1.16, 1.67** | **<0.001** |
| CO, day 2 | 9,624 | **1.35** | **1.12, 1.63** | **0.002** |
| CO, day 3 | 9,624 | **1.37** | **1.13, 1.65** | **0.001** |
| CO, day 4 | 9,624 | **1.40** | **1.16, 1.69** | **<0.001** |
| CO, day 5 | 9,624 | **1.40** | **1.15, 1.68** | **<0.001** |
| CO, day 6 | 9,624 | **1.41** | **1.17, 1.70** | **<0.001** |
| CO, day 7 | 9,624 | **1.46** | **1.21, 1.77** | **<0.001** |
| CO, day 8 | 9,624 | **1.60** | **1.33, 1.93** | **<0.001** |
| CO, day 9 | 9,624 | **1.45** | **1.21, 1.75** | **<0.001** |
| CO, day 10 | 9,624 | **1.33** | **1.10, 1.61** | **0.003** |
| CO, day 11 | 9,624 | **1.38** | **1.14, 1.67** | **<0.001** |
| CO, day 12 | 9,624 | **1.35** | **1.12, 1.64** | **0.002** |
| CO, day 13 | 9,624 | **1.38** | **1.14, 1.67** | **<0.001** |
| CO, day 14 | 9,624 | **1.31** | **1.08, 1.58** | **0.006** |
| CO, day 15 | 9,624 | **1.39** | **1.15, 1.68** | **<0.001** |
| CO, day 16 | 9,624 | **1.30** | **1.07, 1.57** | **0.008** |
| CO, day 17 | 9,624 | **1.36** | **1.12, 1.64** | **0.002** |
| CO, day 18 | 9,624 | **1.40** | **1.16, 1.69** | **<0.001** |
| CO, day 19 | 9,624 | **1.35** | **1.11, 1.63** | **0.002** |
| CO, day 20 | 9,624 | **1.33** | **1.09, 1.60** | **0.004** |
| CO, day 21 | 9,624 | **1.43** | **1.18, 1.73** | **<0.001** |
| CO, day 22 | 9,624 | **1.57** | **1.30, 1.89** | **<0.001** |
| CO, day 23 | 9,624 | **1.39** | **1.15, 1.68** | **<0.001** |
| CO, day 24 | 9,624 | **1.42** | **1.18, 1.72** | **<0.001** |
| CO, day 25 | 9,624 | **1.42** | **1.18, 1.71** | **<0.001** |
| CO, day 26 | 9,624 | **1.34** | **1.11, 1.61** | **0.003** |
| CO, day 27 | 9,624 | **1.45** | **1.20, 1.75** | **<0.001** |
| CO, day 28 | 9,624 | **1.24** | **1.02, 1.49** | **0.03** |
| CO, day 29 | 9,624 | **1.28** | **1.06, 1.54** | **0.01** |
| CO, day 30 | 9,624 | 1.19 | 0.99, 1.44 | 0.07 |
| CO, day 31 | 9,624 | **1.32** | **1.09, 1.58** | **0.004** |
| NO₂ | | | | |
| NO₂, day 0 | 9,624 | **1.26** | **1.12, 1.40** | **<0.001** |
| NO₂, day 1 | 9,624 | **1.24** | **1.10, 1.38** | **<0.001** |
| NO₂, day 2 | 9,624 | **1.31** | **1.16, 1.48** | **<0.001** |
| NO₂, day 3 | 9,624 | **1.29** | **1.14, 1.44** | **<0.001** |
| NO₂, day 4 | 9,624 | **1.21** | **1.07, 1.36** | **0.001** |
| NO₂, day 5 | 9,624 | **1.28** | **1.13, 1.44** | **<0.001** |
| NO₂, day 6 | 9,624 | **1.20** | **1.06, 1.36** | **0.003** |
| NO₂, day 7 | 9,624 | **1.28** | **1.13, 1.44** | **<0.001** |
| NO₂, day 8 | 9,624 | **1.31** | **1.16, 1.47** | **<0.001** |
| NO₂, day 9 | 9,624 | **1.35** | **1.20, 1.51** | **<0.001** |
| NO₂, day 10 | 9,624 | **1.28** | **1.13, 1.44** | **<0.001** |
| NO₂, day 11 | 9,624 | **1.30** | **1.16, 1.46** | **<0.001** |
| NO₂, day 12 | 9,624 | **1.31** | **1.16, 1.47** | **<0.001** |
| NO₂, day 13 | 9,624 | **1.22** | **1.07, 1.37** | **0.001** |
| NO₂, day 14 | 9,624 | **1.24** | **1.09, 1.40** | **<0.001** |
| NO₂, day 15 | 9,624 | **1.20** | **1.05, 1.36** | **0.005** |
| NO₂, day 16 | 9,624 | **1.25** | **1.10, 1.42** | **<0.001** |
| NO₂, day 17 | 9,624 | **1.23** | **1.08, 1.39** | **<0.001** |
| NO₂, day 18 | 9,624 | **1.27** | **1.13, 1.42** | **<0.001** |
| NO₂, day 19 | 9,624 | **1.24** | **1.09, 1.39** | **<0.001** |
| NO₂, day 20 | 9,624 | **1.24** | **1.09, 1.40** | **<0.001** |
| NO₂, day 21 | 9,624 | **1.26** | **1.11, 1.42** | **<0.001** |
| NO₂, day 22 | 9,624 | **1.35** | **1.20, 1.50** | **<0.001** |
| NO₂, day 23 | 9,624 | **1.25** | **1.10, 1.41** | **<0.001** |
| NO₂, day 24 | 9,624 | **1.20** | **1.05, 1.36** | **0.004** |
| NO₂, day 25 | 9,624 | **1.20** | **1.06, 1.35** | **0.004** |
| NO₂, day 26 | 9,624 | **1.19** | **1.04, 1.34** | **0.009** |
| NO₂, day 27 | 9,624 | **1.23** | **1.08, 1.38** | **0.001** |
| NO₂, day 28 | 9,624 | 1.14 | 1.00, 1.30 | 0.05 |
| NO₂, day 29 | 9,624 | **1.19** | **1.04, 1.35** | **0.008** |
| NO₂, day 30 | 9,624 | **1.22** | **1.07, 1.38** | **0.002** |
| NO₂, day 31 | 9,624 | **1.25** | **1.10, 1.41** | **<0.001** |
| O₃ | | | | |
| O₃, day 0 | 9,624 | 0.95 | 0.79, 1.14 | 0.58 |
| O₃, day 1 | 9,624 | 0.95 | 0.79, 1.14 | 0.56 |
| O₃, day 2 | 9,624 | 0.88 | 0.73, 1.05 | 0.16 |
| O₃, day 3 | 9,624 | 0.85 | 0.71, 1.03 | 0.1 |
| O₃, day 4 | 9,624 | 0.91 | 0.76, 1.10 | 0.33 |
| O₃, day 5 | 9,624 | 0.89 | 0.73, 1.07 | 0.21 |
| O₃, day 6 | 9,624 | 0.93 | 0.77, 1.11 | 0.43 |
| O₃, day 7 | 9,624 | 0.94 | 0.78, 1.13 | 0.52 |
| O₃, day 8 | 9,624 | 0.96 | 0.80, 1.15 | 0.67 |
| O₃, day 9 | 9,624 | 0.98 | 0.81, 1.17 | 0.79 |
| O₃, day 10 | 9,624 | 0.99 | 0.83, 1.19 | 0.95 |
| O₃, day 11 | 9,624 | 0.98 | 0.82, 1.18 | 0.84 |
| O₃, day 12 | 9,624 | 0.97 | 0.81, 1.16 | 0.74 |
| O₃, day 13 | 9,624 | 0.98 | 0.82, 1.17 | 0.82 |
| O₃, day 14 | 9,624 | 1.00 | 0.83, 1.20 | >0.99 |
| O₃, day 15 | 9,624 | 1.06 | 0.88, 1.27 | 0.52 |
| O₃, day 16 | 9,624 | 1.14 | 0.95, 1.35 | 0.16 |
| O₃, day 17 | 9,624 | 1.19 | 0.99, 1.41 | 0.06 |
| O₃, day 18 | 9,624 | **1.27** | **1.06, 1.51** | **0.007** |
| O₃, day 19 | 9,624 | **1.26** | **1.06, 1.50** | **0.01** |
| O₃, day 20 | 9,624 | **1.26** | **1.06, 1.50** | **0.009** |
| O₃, day 21 | 9,624 | **1.20** | **1.01, 1.43** | **0.04** |
| O₃, day 22 | 9,624 | 1.07 | 0.90, 1.28 | 0.43 |
| O₃, day 23 | 9,624 | 1.06 | 0.89, 1.27 | 0.51 |
| O₃, day 24 | 9,624 | 1.17 | 0.98, 1.39 | 0.09 |
| O₃, day 25 | 9,624 | 1.12 | 0.94, 1.33 | 0.21 |
| O₃, day 26 | 9,624 | 1.04 | 0.87, 1.24 | 0.65 |
| O₃, day 27 | 9,624 | 1.04 | 0.87, 1.23 | 0.68 |
| O₃, day 28 | 9,624 | 0.99 | 0.83, 1.18 | 0.91 |
| O₃, day 29 | 9,624 | 1.01 | 0.84, 1.20 | 0.94 |
| O₃, day 30 | 9,624 | 0.97 | 0.81, 1.15 | 0.7 |
| O₃, day 31 | 9,624 | 0.98 | 0.82, 1.17 | 0.84 |
| BC | | | | |
| BC, day 0 | 9,624 | **1.20** | **1.04, 1.35** | **0.006** |
| BC, day 1 | 9,624 | **1.20** | **1.05, 1.35** | **0.004** |
| BC, day 2 | 9,624 | **1.16** | **1.02, 1.31** | **0.01** |
| BC, day 3 | 9,624 | **1.15** | **1.01, 1.29** | **0.03** |
| BC, day 4 | 9,624 | 1.12 | 0.98, 1.26 | 0.08 |
| BC, day 5 | 9,624 | **1.17** | **1.01, 1.32** | **0.02** |
| BC, day 6 | 9,624 | **1.18** | **1.03, 1.34** | **0.01** |
| BC, day 7 | 9,624 | **1.20** | **1.04, 1.37** | **0.007** |
| BC, day 8 | 9,624 | **1.19** | **1.04, 1.35** | **0.009** |
| BC, day 9 | 9,624 | **1.22** | **1.08, 1.35** | **<0.001** |
| BC, day 10 | 9,624 | **1.20** | **1.05, 1.34** | **0.003** |
| BC, day 11 | 9,624 | **1.20** | **1.05, 1.36** | **0.005** |
| BC, day 12 | 9,624 | **1.18** | **1.04, 1.32** | **0.008** |
| BC, day 13 | 9,624 | **1.13** | **0.99, 1.27** | **0.04** |
| BC, day 14 | 9,624 | **1.16** | **1.01, 1.31** | **0.03** |
| BC, day 15 | 9,624 | **1.20** | **1.06, 1.35** | **0.003** |
| BC, day 16 | 9,624 | **1.14** | **1.00, 1.28** | **0.03** |
| BC, day 17 | 9,624 | **1.18** | **1.03, 1.35** | **0.01** |
| BC, day 18 | 9,624 | **1.17** | **1.02, 1.32** | **0.01** |
| BC, day 19 | 9,624 | 1.04 | 0.89, 1.20 | 0.62 |
| BC, day 20 | 9,624 | 1.03 | 0.89, 1.19 | 0.66 |
| BC, day 21 | 9,624 | 1.09 | 0.95, 1.24 | 0.21 |
| BC, day 22 | 9,624 | **1.21** | **1.07, 1.36** | **0.002** |
| BC, day 23 | 9,624 | **1.15** | **1.02, 1.28** | **0.02** |
| BC, day 24 | 9,624 | **1.19** | **1.06, 1.32** | **0.002** |
| BC, day 25 | 9,624 | **1.14** | **1.00, 1.30** | **0.04** |
| BC, day 26 | 9,624 | **1.21** | **1.05, 1.37** | **0.006** |
| BC, day 27 | 9,624 | **1.24** | **1.10, 1.40** | **<0.001** |
| BC, day 28 | 9,624 | 1.07 | 0.93, 1.23 | 0.32 |
| BC, day 29 | 9,624 | **1.16** | **1.02, 1.32** | **0.02** |
| BC, day 30 | 9,624 | **1.24** | **1.09, 1.39** | **<0.001** |
| BC, day 31 | 9,624 | **1.17** | **1.02, 1.33** | **0.02** |

**Table S4: Exploratory univariable associations between meteorological and air pollution variables and overall pathogen detection, adjusted for season**

| Variable | N | OR | 95% CI | p-value |
| --- | --- | --- | --- | --- |
| Temperature | | | | |
| Temperature, day 0 | 4,446 | **0.90** | **0.82, 1.0** | **0.04** |
| Temperature, day 1 | 4,446 | 0.98 | 0.89, 1.08 | 0.69 |
| Temperature, day 2 | 4,446 | 0.98 | 0.89, 1.07 | 0.62 |
| Temperature, day 3 | 4,446 | 1.00 | 0.91, 1.10 | >0.99 |
| Temperature, day 4 | 4,446 | 0.97 | 0.88, 1.07 | 0.51 |
| Temperature, day 5 | 4,446 | 0.93 | 0.84, 1.02 | 0.12 |
| Temperature, day 6 | 4,446 | 0.94 | 0.85, 1.03 | 0.21 |
| Temperature, day 7 | 4,446 | 0.94 | 0.86, 1.04 | 0.23 |
| Temperature, day 8 | 4,446 | 0.92 | 0.84, 1.01 | 0.08 |
| Temperature, day 9 | 4,446 | 0.92 | 0.84, 1.01 | 0.09 |
| Temperature, day 10 | 4,446 | 0.91 | 0.83, 1.00 | 0.05 |
| Temperature, day 11 | 4,446 | 0.94 | 0.86, 1.03 | 0.2 |
| Temperature, day 12 | 4,446 | 0.99 | 0.90, 1.09 | 0.89 |
| Temperature, day 13 | 4,446 | 1.01 | 0.92, 1.11 | 0.88 |
| Temperature, day 14 | 4,446 | 0.98 | 0.89, 1.08 | 0.72 |
| Temperature, day 15 | 4,446 | 0.98 | 0.90, 1.08 | 0.73 |
| Temperature, day 16 | 4,446 | 0.98 | 0.89, 1.08 | 0.67 |
| Temperature, day 17 | 4,446 | 0.99 | 0.90, 1.08 | 0.79 |
| Temperature, day 18 | 4,446 | 0.95 | 0.86, 1.04 | 0.24 |
| Temperature, day 19 | 4,446 | 0.94 | 0.86, 1.03 | 0.21 |
| Temperature, day 20 | 4,446 | 0.93 | 0.84, 1.02 | 0.11 |
| Temperature, day 21 | 4,446 | **0.90** | **0.82, 0.99** | **0.03** |
| Temperature, day 22 | 4,446 | 0.96 | 0.87, 1.05 | 0.37 |
| Temperature, day 23 | 4,446 | 0.96 | 0.87, 1.05 | 0.35 |
| Temperature, day 24 | 4,446 | 0.93 | 0.85, 1.02 | 0.14 |
| Temperature, day 25 | 4,446 | 0.93 | 0.84, 1.02 | 0.11 |
| Temperature, day 26 | 4,446 | 0.93 | 0.85, 1.02 | 0.13 |
| Temperature, day 27 | 4,446 | 0.95 | 0.86, 1.04 | 0.24 |
| Temperature, day 28 | 4,446 | 0.93 | 0.85, 1.03 | 0.15 |
| Temperature, day 29 | 4,446 | 0.95 | 0.87, 1.05 | 0.33 |
| Temperature, day 30 | 4,446 | 0.99 | 0.90, 1.09 | 0.83 |
| Temperature, day 31 | 4,446 | 0.98 | 0.89, 1.07 | 0.65 |
| Humidity | | | | |
| Humidity, day 0 | 4,446 | 1.03 | 0.96, 1.11 | 0.41 |
| Humidity, day 1 | 4,446 | 1.02 | 0.95, 1.10 | 0.56 |
| Humidity, day 2 | 4,446 | 1.05 | 0.98, 1.13 | 0.17 |
| Humidity, day 3 | 4,446 | 1.06 | 0.99, 1.14 | 0.11 |
| Humidity, day 4 | 4,446 | 1.04 | 0.97, 1.12 | 0.25 |
| Humidity, day 5 | 4,446 | 1.04 | 0.96, 1.12 | 0.33 |
| Humidity, day 6 | 4,446 | 1.03 | 0.96, 1.11 | 0.46 |
| Humidity, day 7 | 4,446 | 1.02 | 0.95, 1.10 | 0.54 |
| Humidity, day 8 | 4,446 | 1.03 | 0.96, 1.11 | 0.38 |
| Humidity, day 9 | 4,446 | 1.06 | 0.99, 1.14 | 0.09 |
| Humidity, day 10 | 4,446 | 1.06 | 0.98, 1.14 | 0.13 |
| Humidity, day 11 | 4,446 | 1.03 | 0.96, 1.11 | 0.38 |
| Humidity, day 12 | 4,446 | 0.99 | 0.92, 1.07 | 0.82 |
| Humidity, day 13 | 4,446 | 0.97 | 0.90, 1.05 | 0.46 |
| Humidity, day 14 | 4,446 | 1.02 | 0.94, 1.09 | 0.69 |
| Humidity, day 15 | 4,446 | 1.00 | 0.93, 1.08 | >0.99 |
| Humidity, day 16 | 4,446 | 1.05 | 0.98, 1.13 | 0.19 |
| Humidity, day 17 | 4,446 | 1.08 | 1.00, 1.16 | 0.05 |
| Humidity, day 18 | 4,446 | 1.03 | 0.96, 1.10 | 0.46 |
| Humidity, day 19 | 4,446 | 1.01 | 0.94, 1.09 | 0.78 |
| Humidity, day 20 | 4,446 | 1.01 | 0.94, 1.08 | 0.89 |
| Humidity, day 21 | 4,446 | 0.99 | 0.93, 1.07 | 0.88 |
| Humidity, day 22 | 4,446 | 0.95 | 0.89, 1.02 | 0.2 |
| Humidity, day 23 | 4,446 | 0.99 | 0.92, 1.06 | 0.79 |
| Humidity, day 24 | 4,446 | 0.99 | 0.92, 1.06 | 0.72 |
| Humidity, day 25 | 4,446 | 0.97 | 0.90, 1.04 | 0.41 |
| Humidity, day 26 | 4,446 | 1.02 | 0.95, 1.10 | 0.59 |
| Humidity, day 27 | 4,446 | 1.00 | 0.93, 1.08 | 0.95 |
| Humidity, day 28 | 4,446 | 1.04 | 0.97, 1.11 | 0.32 |
| Humidity, day 29 | 4,446 | 1.02 | 0.95, 1.09 | 0.63 |
| Humidity, day 30 | 4,446 | 0.97 | 0.90, 1.04 | 0.4 |
| Humidity, day 31 | 4,446 | 0.99 | 0.92, 1.06 | 0.67 |
| Air pressure | | | | |
| Air pressure, day 0 | 4,446 | **1.06** | **1.00, 1.13** | **0.04** |
| Air pressure, day 1 | 4,446 | 1.04 | 0.99, 1.11 | 0.13 |
| Air pressure, day 2 | 4,446 | 1.05 | 0.99, 1.11 | 0.1 |
| Air pressure, day 3 | 4,446 | 1.05 | 1.00, 1.12 | 0.07 |
| Air pressure, day 4 | 4,446 | **1.08** | **1.02, 1.14** | **0.008** |
| Air pressure, day 5 | 4,446 | **1.10** | **1.03, 1.16** | **0.002** |
| Air pressure, day 6 | 4,446 | **1.09** | **1.03, 1.15** | **0.004** |
| Air pressure, day 7 | 4,446 | **1.08** | **1.02, 1.15** | **0.005** |
| Air pressure, day 8 | 4,446 | **1.08** | **1.02, 1.15** | **0.007** |
| Air pressure, day 9 | 4,446 | **1.08** | **1.02, 1.14** | **0.01** |
| Air pressure, day 10 | 4,446 | **1.07** | **1.01, 1.13** | **0.03** |
| Air pressure, day 11 | 4,446 | **1.07** | **1.01, 1.13** | **0.03** |
| Air pressure, day 12 | 4,446 | **1.06** | **1.00, 1.12** | **0.04** |
| Air pressure, day 13 | 4,446 | 1.05 | 1.00, 1.12 | 0.07 |
| Air pressure, day 14 | 4,446 | 1.04 | 0.99, 1.10 | 0.15 |
| Air pressure, day 15 | 4,446 | 1.05 | 0.99, 1.11 | 0.12 |
| Air pressure, day 16 | 4,446 | 1.05 | 0.99, 1.11 | 0.1 |
| Air pressure, day 17 | 4,446 | 1.04 | 0.98, 1.10 | 0.18 |
| Air pressure, day 18 | 4,446 | 1.04 | 0.98, 1.10 | 0.18 |
| Air pressure, day 19 | 4,446 | 1.04 | 0.98, 1.10 | 0.2 |
| Air pressure, day 20 | 4,446 | 1.04 | 0.98, 1.10 | 0.22 |
| Air pressure, day 21 | 4,446 | 1.03 | 0.98, 1.10 | 0.24 |
| Air pressure, day 22 | 4,446 | 1.03 | 0.97, 1.09 | 0.3 |
| Air pressure, day 23 | 4,446 | 1.04 | 0.98, 1.10 | 0.21 |
| Air pressure, day 24 | 4,446 | 1.06 | 1.00, 1.12 | 0.05 |
| Air pressure, day 25 | 4,446 | **1.06** | **1.00, 1.12** | **0.04** |
| Air pressure, day 26 | 4,446 | 1.06 | 1.00, 1.12 | 0.05 |
| Air pressure, day 27 | 4,446 | 1.06 | 1.00, 1.12 | 0.06 |
| Air pressure, day 28 | 4,446 | 1.05 | 0.99, 1.11 | 0.09 |
| Air pressure, day 29 | 4,446 | 1.05 | 0.99, 1.11 | 0.12 |
| Air pressure, day 30 | 4,446 | 1.05 | 0.99, 1.11 | 0.09 |
| Air pressure, day 31 | 4,446 | 1.05 | 0.99, 1.11 | 0.09 |
| PM_2.5_ | | | | |
| PM_2.5_, day 0 | 4,446 | **0.88** | **0.82, 0.94** | **<0.001** |
| PM_2.5_, day 1 | 4,446 | 0.95 | 0.88, 1.01 | 0.11 |
| PM_2.5_, day 2 | 4,446 | **0.92** | **0.86, 0.99** | **0.02** |
| PM_2.5_, day 3 | 4,446 | 0.94 | 0.88, 1.00 | 0.07 |
| PM_2.5_, day 4 | 4,446 | 0.99 | 0.92, 1.06 | 0.72 |
| PM_2.5_, day 5 | 4,446 | 0.95 | 0.89, 1.02 | 0.14 |
| PM_2.5_, day 6 | 4,446 | **0.90** | **0.84, 0.96** | **0.003** |
| PM_2.5_, day 7 | 4,446 | **0.89** | **0.83, 0.95** | **<0.001** |
| PM_2.5_, day 8 | 4,446 | **0.86** | **0.80, 0.93** | **<0.001** |
| PM_2.5_, day 9 | 4,446 | **0.90** | **0.84, 0.96** | **0.003** |
| PM_2.5_, day 10 | 4,446 | **0.89** | **0.83, 0.95** | **<0.001** |
| PM_2.5_, day 11 | 4,446 | **0.88** | **0.82, 0.95** | **<0.001** |
| PM_2.5_, day 12 | 4,446 | **0.87** | **0.81, 0.93** | **<0.001** |
| PM_2.5_, day 13 | 4,446 | **0.88** | **0.82, 0.94** | **<0.001** |
| PM_2.5_, day 14 | 4,446 | **0.90** | **0.84, 0.96** | **0.002** |
| PM_2.5_, day 15 | 4,446 | **0.89** | **0.83, 0.96** | **0.001** |
| PM_2.5_, day 16 | 4,446 | **0.90** | **0.84, 0.96** | **0.003** |
| PM_2.5_, day 17 | 4,446 | 0.93 | 0.87, 1.00 | 0.05 |
| PM_2.5_, day 18 | 4,446 | **0.89** | **0.83, 0.95** | **0.001** |
| PM_2.5_, day 19 | 4,446 | **0.91** | **0.85, 0.97** | **0.007** |
| PM_2.5_, day 20 | 4,446 | **0.92** | **0.86, 0.98** | **0.01** |
| PM_2.5_, day 21 | 4,446 | **0.93** | **0.86, 0.99** | **0.02** |
| PM_2.5_, day 22 | 4,446 | **0.92** | **0.86, 0.99** | **0.02** |
| PM_2.5_, day 23 | 4,446 | **0.92** | **0.86, 0.99** | **0.02** |
| PM_2.5_, day 24 | 4,446 | **0.91** | **0.85, 0.97** | **0.007** |
| PM_2.5_, day 25 | 4,446 | **0.89** | **0.83, 0.95** | **<0.001** |
| PM_2.5_, day 26 | 4,446 | **0.90** | **0.84, 0.96** | **0.003** |
| PM_2.5_, day 27 | 4,446 | **0.88** | **0.82, 0.95** | **<0.001** |
| PM_2.5_, day 28 | 4,446 | **0.92** | **0.86, 0.99** | **0.02** |
| PM_2.5_, day 29 | 4,446 | 0.94 | 0.88, 1.01 | 0.07 |
| PM_2.5_, day 30 | 4,446 | **0.91** | **0.85, 0.98** | **0.008** |
| PM_2.5_, day 31 | 4,446 | **0.90** | **0.84, 0.96** | **0.003** |
| SO₂ | | | | |
| SO₂, day 0 | 4,446 | 0.97 | 0.92, 1.04 | 0.4 |
| SO₂, day 1 | 4,446 | 0.98 | 0.92, 1.05 | 0.59 |
| SO₂, day 2 | 4,446 | 0.94 | 0.88, 1.01 | 0.07 |
| SO₂, day 3 | 4,446 | 0.94 | 0.88, 1.00 | 0.06 |
| SO₂, day 4 | 4,446 | **0.93** | **0.87, 0.99** | **0.02** |
| SO₂, day 5 | 4,446 | **0.88** | **0.83, 0.94** | **<0.001** |
| SO₂, day 6 | 4,446 | **0.89** | **0.83, 0.95** | **<0.001** |
| SO₂, day 7 | 4,446 | **0.90** | **0.84, 0.96** | **<0.001** |
| SO₂, day 8 | 4,446 | **0.90** | **0.84, 0.95** | **<0.001** |
| SO₂, day 9 | 4,446 | **0.90** | **0.84, 0.96** | **0.001** |
| SO₂, day 10 | 4,446 | **0.89** | **0.83, 0.95** | **<0.001** |
| SO₂, day 11 | 4,446 | **0.94** | **0.88, 0.99** | **0.03** |
| SO₂, day 12 | 4,446 | 0.94 | 0.89, 1.00 | 0.07 |
| SO₂, day 13 | 4,446 | 0.94 | 0.88, 1.00 | 0.05 |
| SO₂, day 14 | 4,446 | **0.93** | **0.88, 0.99** | **0.03** |
| SO₂, day 15 | 4,446 | **0.93** | **0.87, 0.99** | **0.03** |
| SO₂, day 16 | 4,446 | **0.88** | **0.83, 0.94** | **<0.001** |
| SO₂, day 17 | 4,446 | **0.93** | **0.87, 0.99** | **0.03** |
| SO₂, day 18 | 4,446 | **0.93** | **0.87, 0.99** | **0.03** |
| SO₂, day 19 | 4,446 | 0.96 | 0.90, 1.02 | 0.19 |
| SO₂, day 20 | 4,446 | 0.98 | 0.92, 1.04 | 0.56 |
| SO₂, day 21 | 4,446 | 1.00 | 0.94, 1.06 | 0.91 |
| SO₂, day 22 | 4,446 | 0.97 | 0.91, 1.03 | 0.35 |
| SO₂, day 23 | 4,446 | 0.98 | 0.92, 1.04 | 0.49 |
| SO₂, day 24 | 4,446 | 0.94 | 0.88, 1.00 | 0.06 |
| SO₂, day 25 | 4,446 | 0.96 | 0.90, 1.02 | 0.23 |
| SO₂, day 26 | 4,446 | 0.97 | 0.91, 1.03 | 0.33 |
| SO₂, day 27 | 4,446 | **0.92** | **0.86, 0.98** | **0.007** |
| SO₂, day 28 | 4,446 | **0.93** | **0.87, 0.99** | **0.02** |
| SO₂, day 29 | 4,446 | **0.93** | **0.87, 0.99** | **0.02** |
| SO₂, day 30 | 4,446 | **0.92** | **0.86, 0.97** | **0.006** |
| SO₂, day 31 | 4,446 | 0.95 | 0.90, 1.02 | 0.15 |
| CO | | | | |
| CO, day 0 | 4,446 | 1.00 | 0.92, 1.09 | 0.98 |
| CO, day 1 | 4,446 | 0.98 | 0.90, 1.07 | 0.67 |
| CO, day 2 | 4,446 | 1.02 | 0.93, 1.12 | 0.65 |
| CO, day 3 | 4,446 | 0.99 | 0.90, 1.08 | 0.77 |
| CO, day 4 | 4,446 | 0.98 | 0.89, 1.07 | 0.65 |
| CO, day 5 | 4,446 | 1.00 | 0.91, 1.09 | 0.94 |
| CO, day 6 | 4,446 | 1.00 | 0.92, 1.10 | 0.96 |
| CO, day 7 | 4,446 | 0.99 | 0.90, 1.08 | 0.75 |
| CO, day 8 | 4,446 | 0.96 | 0.88, 1.05 | 0.39 |
| CO, day 9 | 4,446 | 0.95 | 0.86, 1.04 | 0.24 |
| CO, day 10 | 4,446 | 0.93 | 0.85, 1.02 | 0.11 |
| CO, day 11 | 4,446 | 0.96 | 0.87, 1.05 | 0.34 |
| CO, day 12 | 4,446 | 0.97 | 0.89, 1.06 | 0.5 |
| CO, day 13 | 4,446 | 0.94 | 0.85, 1.03 | 0.15 |
| CO, day 14 | 4,446 | 0.94 | 0.86, 1.03 | 0.2 |
| CO, day 15 | 4,446 | 0.92 | 0.84, 1.01 | 0.09 |
| CO, day 16 | 4,446 | 0.94 | 0.86, 1.03 | 0.2 |
| CO, day 17 | 4,446 | 0.96 | 0.87, 1.05 | 0.36 |
| CO, day 18 | 4,446 | 0.97 | 0.88, 1.06 | 0.46 |
| CO, day 19 | 4,446 | 0.98 | 0.90, 1.07 | 0.68 |
| CO, day 20 | 4,446 | 0.98 | 0.90, 1.07 | 0.67 |
| CO, day 21 | 4,446 | 0.97 | 0.89, 1.07 | 0.56 |
| CO, day 22 | 4,446 | 0.95 | 0.87, 1.04 | 0.28 |
| CO, day 23 | 4,446 | 0.94 | 0.86, 1.03 | 0.21 |
| CO, day 24 | 4,446 | 0.97 | 0.89, 1.06 | 0.52 |
| CO, day 25 | 4,446 | 0.98 | 0.90, 1.07 | 0.69 |
| CO, day 26 | 4,446 | 0.95 | 0.87, 1.04 | 0.25 |
| CO, day 27 | 4,446 | 0.92 | 0.84, 1.01 | 0.09 |
| CO, day 28 | 4,446 | 0.95 | 0.87, 1.04 | 0.27 |
| CO, day 29 | 4,446 | 0.95 | 0.87, 1.04 | 0.23 |
| CO, day 30 | 4,446 | 0.99 | 0.91, 1.09 | 0.89 |
| CO, day 31 | 4,446 | 0.98 | 0.90, 1.08 | 0.72 |
| NO₂ | | | | |
| NO₂, day 0 | 4,446 | 0.97 | 0.91, 1.04 | 0.42 |
| NO₂, day 1 | 4,446 | 0.99 | 0.92, 1.06 | 0.75 |
| NO₂, day 2 | 4,446 | 1.01 | 0.95, 1.09 | 0.69 |
| NO₂, day 3 | 4,446 | 0.96 | 0.90, 1.03 | 0.26 |
| NO₂, day 4 | 4,446 | 0.96 | 0.89, 1.03 | 0.24 |
| NO₂, day 5 | 4,446 | 0.98 | 0.91, 1.05 | 0.55 |
| NO₂, day 6 | 4,446 | 0.97 | 0.90, 1.03 | 0.33 |
| NO₂, day 7 | 4,446 | 1.02 | 0.96, 1.09 | 0.51 |
| NO₂, day 8 | 4,446 | 0.99 | 0.93, 1.06 | 0.83 |
| NO₂, day 9 | 4,446 | 0.96 | 0.89, 1.03 | 0.22 |
| NO₂, day 10 | 4,446 | 0.97 | 0.91, 1.04 | 0.41 |
| NO₂, day 11 | 4,446 | 0.96 | 0.90, 1.02 | 0.21 |
| NO₂, day 12 | 4,446 | 0.94 | 0.88, 1.01 | 0.1 |
| NO₂, day 13 | 4,446 | 0.94 | 0.88, 1.01 | 0.09 |
| NO₂, day 14 | 4,446 | 0.98 | 0.92, 1.06 | 0.66 |
| NO₂, day 15 | 4,446 | 0.99 | 0.93, 1.06 | 0.81 |
| NO₂, day 16 | 4,446 | 1.00 | 0.93, 1.07 | 0.96 |
| NO₂, day 17 | 4,446 | 1.02 | 0.95, 1.10 | 0.51 |
| NO₂, day 18 | 4,446 | 1.03 | 0.96, 1.10 | 0.38 |
| NO₂, day 19 | 4,446 | 1.05 | 0.98, 1.12 | 0.15 |
| NO₂, day 20 | 4,446 | 1.02 | 0.95, 1.09 | 0.61 |
| NO₂, day 21 | 4,446 | 1.01 | 0.94, 1.08 | 0.75 |
| NO₂, day 22 | 4,446 | 0.98 | 0.91, 1.05 | 0.59 |
| NO₂, day 23 | 4,446 | 0.99 | 0.92, 1.06 | 0.68 |
| NO₂, day 24 | 4,446 | 1.02 | 0.95, 1.10 | 0.51 |
| NO₂, day 25 | 4,446 | 1.00 | 0.93, 1.07 | 0.9 |
| NO₂, day 26 | 4,446 | 1.00 | 0.94, 1.07 | 0.93 |
| NO₂, day 27 | 4,446 | 0.95 | 0.88, 1.01 | 0.13 |
| NO₂, day 28 | 4,446 | 0.95 | 0.88, 1.02 | 0.13 |
| NO₂, day 29 | 4,446 | 0.94 | 0.88, 1.01 | 0.09 |
| NO₂, day 30 | 4,446 | 0.95 | 0.88, 1.01 | 0.12 |
| NO₂, day 31 | 4,446 | 0.99 | 0.92, 1.06 | 0.73 |
| O₃ | | | | |
| O₃, day 0 | 4,446 | 1.06 | 0.98, 1.15 | 0.14 |
| O₃, day 1 | 4,446 | 1.02 | 0.94, 1.10 | 0.62 |
| O₃, day 2 | 4,446 | 1.00 | 0.92, 1.08 | >0.99 |
| O₃, day 3 | 4,446 | 0.98 | 0.91, 1.07 | 0.69 |
| O₃, day 4 | 4,446 | 0.98 | 0.90, 1.06 | 0.62 |
| O₃, day 5 | 4,446 | 0.99 | 0.91, 1.07 | 0.71 |
| O₃, day 6 | 4,446 | 1.05 | 0.97, 1.14 | 0.19 |
| O₃, day 7 | 4,446 | **1.10** | **1.01, 1.19** | **0.02** |
| O₃, day 8 | 4,446 | 1.08 | 0.99, 1.17 | 0.07 |
| O₃, day 9 | 4,446 | **1.10** | **1.01, 1.19** | **0.02** |
| O₃, day 10 | 4,446 | 1.06 | 0.98, 1.14 | 0.17 |
| O₃, day 11 | 4,446 | **1.10** | **1.01, 1.19** | **0.02** |
| O₃, day 12 | 4,446 | **1.11** | **1.02, 1.20** | **0.01** |
| O₃, day 13 | 4,446 | 1.08 | 1.00, 1.17 | 0.05 |
| O₃, day 14 | 4,446 | 1.07 | 0.99, 1.16 | 0.07 |
| O₃, day 15 | 4,446 | 1.05 | 0.97, 1.13 | 0.25 |
| O₃, day 16 | 4,446 | 1.03 | 0.95, 1.12 | 0.41 |
| O₃, day 17 | 4,446 | 1.04 | 0.96, 1.13 | 0.32 |
| O₃, day 18 | 4,446 | 1.03 | 0.95, 1.12 | 0.42 |
| O₃, day 19 | 4,446 | 1.01 | 0.93, 1.10 | 0.77 |
| O₃, day 20 | 4,446 | 1.03 | 0.95, 1.11 | 0.54 |
| O₃, day 21 | 4,446 | 1.07 | 0.98, 1.15 | 0.12 |
| O₃, day 22 | 4,446 | 1.04 | 0.96, 1.12 | 0.39 |
| O₃, day 23 | 4,446 | 1.01 | 0.93, 1.09 | 0.85 |
| O₃, day 24 | 4,446 | 1.02 | 0.94, 1.10 | 0.65 |
| O₃, day 25 | 4,446 | 1.04 | 0.96, 1.13 | 0.3 |
| O₃, day 26 | 4,446 | 1.05 | 0.97, 1.14 | 0.2 |
| O₃, day 27 | 4,446 | 1.06 | 0.98, 1.15 | 0.12 |
| O₃, day 28 | 4,446 | **1.10** | **1.02, 1.19** | **0.02** |
| O₃, day 29 | 4,446 | 1.07 | 0.99, 1.16 | 0.1 |
| O₃, day 30 | 4,446 | 1.04 | 0.96, 1.13 | 0.29 |
| O₃, day 31 | 4,446 | 1.06 | 0.98, 1.15 | 0.12 |
| BC | | | | |
| BC, day 0 | 4,446 | 0.95 | 0.87, 1.03 | 0.2 |
| BC, day 1 | 4,446 | 1.03 | 0.94, 1.12 | 0.54 |
| BC, day 2 | 4,446 | 0.99 | 0.91, 1.08 | 0.83 |
| BC, day 3 | 4,446 | 1.01 | 0.93, 1.10 | 0.8 |
| BC, day 4 | 4,446 | 1.00 | 0.92, 1.08 | >0.99 |
| BC, day 5 | 4,446 | 0.95 | 0.87, 1.03 | 0.23 |
| BC, day 6 | 4,446 | 0.96 | 0.88, 1.04 | 0.35 |
| BC, day 7 | 4,446 | 0.97 | 0.89, 1.06 | 0.53 |
| BC, day 8 | 4,446 | 0.93 | 0.86, 1.01 | 0.08 |
| BC, day 9 | 4,446 | **0.92** | **0.85, 0.99** | **0.03** |
| BC, day 10 | 4,446 | **0.90** | **0.83, 0.98** | **0.01** |
| BC, day 11 | 4,446 | **0.91** | **0.84, 0.99** | **0.03** |
| BC, day 12 | 4,446 | **0.89** | **0.82, 0.97** | **0.007** |
| BC, day 13 | 4,446 | **0.87** | **0.80, 0.95** | **0.002** |
| BC, day 14 | 4,446 | 0.92 | 0.85, 1.00 | 0.06 |
| BC, day 15 | 4,446 | 0.94 | 0.86, 1.02 | 0.14 |
| BC, day 16 | 4,446 | **0.91** | **0.84, 0.99** | **0.03** |
| BC, day 17 | 4,446 | 0.92 | 0.85, 1.00 | 0.06 |
| BC, day 18 | 4,446 | 0.94 | 0.87, 1.02 | 0.15 |
| BC, day 19 | 4,446 | 0.97 | 0.89, 1.05 | 0.47 |
| BC, day 20 | 4,446 | 0.94 | 0.87, 1.02 | 0.14 |
| BC, day 21 | 4,446 | 0.98 | 0.90, 1.06 | 0.6 |
| BC, day 22 | 4,446 | 1.00 | 0.92, 1.08 | >0.99 |
| BC, day 23 | 4,446 | 0.97 | 0.90, 1.05 | 0.47 |
| BC, day 24 | 4,446 | 0.98 | 0.90, 1.05 | 0.52 |
| BC, day 25 | 4,446 | 0.96 | 0.89, 1.04 | 0.35 |
| BC, day 26 | 4,446 | 0.97 | 0.89, 1.06 | 0.5 |
| BC, day 27 | 4,446 | **0.92** | **0.84, 1.00** | **0.04** |
| BC, day 28 | 4,446 | 0.95 | 0.88, 1.03 | 0.25 |
| BC, day 29 | 4,446 | 0.97 | 0.89, 1.05 | 0.44 |
| BC, day 30 | 4,446 | 0.99 | 0.91, 1.07 | 0.77 |
| BC, day 31 | 4,446 | 0.98 | 0.90, 1.06 | 0.56 |

**Table S5: STROBE Statement—Checklist of items that should be included in reports of *cohort studies***

|  | Item No | Recommendation | Page No |
| --- | --- | --- | --- |
| **Title and abstract** | 1 | (*a*) Indicate the study’s design with a commonly used term in the title or the abstract | 1 |
|  |  | (*b*) Provide in the abstract an informative and balanced summary of what was done and what was found | 2-3 |
| Introduction | | | |
| Background/rationale | 2 | Explain the scientific background and rationale for the investigation being reported | 4 |
| Objectives | 3 | State specific objectives, including any prespecified hypotheses | 4, 7 |
| Methods | | | |
| Study design | 4 | Present key elements of study design early in the paper | 4-5 |
| Setting | 5 | Describe the setting, locations, and relevant dates, including periods of recruitment, exposure, follow-up, and data collection | 4-5 |
| Participants | 6 | (*a*) Give the eligibility criteria, and the sources and methods of selection of participants. Describe methods of follow-up | 4-5 |
|  |  | (*b*) For matched studies, give matching criteria and number of exposed and unexposed | / |
| Variables | 7 | Clearly define all outcomes, exposures, predictors, potential confounders, and effect modifiers. Give diagnostic criteria, if applicable | 7-8 |
| Data sources/ measurement | 8* | For each variable of interest, give sources of data and details of methods of assessment (measurement). Describe comparability of assessment methods if there is more than one group | 4-6 |
| Bias | 9 | Describe any efforts to address potential sources of bias | 7-8 |
| Study size | 10 | Explain how the study size was arrived at | / |
| Quantitative variables | 11 | Explain how quantitative variables were handled in the analyses. If applicable, describe which groupings were chosen and why | 7-8 |
| Statistical methods | 12 | (*a*) Describe all statistical methods, including those used to control for confounding | 7-8 |
|  |  | (*b*) Describe any methods used to examine subgroups and interactions | / |
|  |  | (*c*) Explain how missing data were addressed | 7-8 |
|  |  | (*d*) If applicable, explain how loss to follow-up was addressed | / |
|  |  | (*e*) Describe any sensitivity analyses | 6-8 |
| Results | | |  |
| Participants | 13* | (a) Report numbers of individuals at each stage of study—eg numbers potentially eligible, examined for eligibility, confirmed eligible, included in the study, completing follow-up, and analysed | 9 |
|  |  | (b) Give reasons for non-participation at each stage | 9 |
|  |  | (c) Consider use of a flow diagram | 9 |
| Descriptive data | 14* | (a) Give characteristics of study participants (eg demographic, clinical, social) and information on exposures and potential confounders | 9-10 |
|  |  | (b) Indicate number of participants with missing data for each variable of interest | 11-12 |
|  |  | (c) Summarise follow-up time (eg, average and total amount) | / |
| Outcome data | 15* | Report numbers of outcome events or summary measures over time | 10 |

| Main results | 16 | (*a*) Give unadjusted estimates and, if applicable, confounder-adjusted estimates and their precision (eg, 95% confidence interval). Make clear which confounders were adjusted for and why they were included | 13-15 |
| --- | --- | --- | --- |
|  |  | (*b*) Report category boundaries when continuous variables were categorized | / |
|  |  | (*c*) If relevant, consider translating estimates of relative risk into absolute risk for a meaningful time period | / |
| Other analyses | 17 | Report other analyses done—eg analyses of subgroups and interactions, and sensitivity analyses | 15-18 |
| Discussion | | | |
| Key results | 18 | Summarise key results with reference to study objectives | 19 |
| Limitations | 19 | Discuss limitations of the study, taking into account sources of potential bias or imprecision. Discuss both direction and magnitude of any potential bias | 19, 22-23 |
| Interpretation | 20 | Give a cautious overall interpretation of results considering objectives, limitations, multiplicity of analyses, results from similar studies, and other relevant evidence | 19-23 |
| Generalisability | 21 | Discuss the generalisability (external validity) of the study results | 23 |
| Other information | | | |
| Funding | 22 | Give the source of funding and the role of the funders for the present study and, if applicable, for the original study on which the present article is based | 25-26 |

**Note:** An Explanation and Elaboration article discusses each checklist item and gives methodological background and published examples of transparent reporting. The STROBE checklist is best used in conjunction with this article (freely available on the Web sites of PLoS Medicine at http://www.plosmedicine.org/, Annals of Internal Medicine at http://www.annals.org/, and Epidemiology at http://www.epidem.com/). Information on the STROBE Initiative is available at http://www.strobe-statement.org.

**Table S6a: Univariable associations of clinical variables**

|  | Sputum  quality^a^ | Treatment  setting^a^ | Prior antibiotic therapy^a^ | CURB65 >=2^a^ | Age^b^ | BMI^b^ | Sex^a^ | Active  smoking^a^ | Respiratory  disease^a^ | Malignancy^a^ | Influenza  vaccination^a^ | Pneumococcal  vaccination^a^ |
| --- | --- | --- | --- | --- | --- | --- | --- | --- | --- | --- | --- | --- |
| Positive sputum culture | <0.001 | 0.26 | <0.001 | <0.001 | <0.001 | 0.01 | 0.01 | 0.31 | <0.001 | 0.1 | 0.01 | <0.001 |
| High leucocyte count | / | <0.001 | 0.45 | 0.21 | <0.001 | 0.92 | 0.04 | 0.21 | 0.69 | 0.12 | 0.48 | 0.41 |
| High epithelial cell count | / | <0.001 | 0.64 | 0.01 | 0.26 | 0.01 | 0.51 | 0.11 | 0.50 | 0.95 | 0.10 | 0.72 |
| Pneumococcal antigen in urine | / | <0.001 | <0.01 | <0.001 | 0.73 | <0.001 | 0.45 | 0.00 | 0.00 | 0.19 | 0.15 | 0.38 |
| *S. pneumoniae* | <0.001 | 0.42 | <0.001 | 0.03 | <0.001 | <0.001 | 0.17 | <0.001 | 0.00 | 0.41 | 0.77 | 0.51 |
| *H. influenzae* | <0.001 | <0.01 | 0.02 | 0.12 | <0.01 | 0.09 | <0.001 | 0.72 | 0.30 | 0.09 | 0.89 | 0.27 |
| *H. parainfluenzae* | 0.94 | 0.02 | 0.74 | 0.00 | <0.001 | 0.88 | 0.19 | 0.94 | 0.08 | 0.15 | 0.05 | <0.01 |
| *C. albicans* | <0.01 | <0.01 | 0.02 | 0.03 | 0.01 | 0.08 | 0.75 | 0.57 | <0.001 | 0.80 | 0.99 | 0.35 |
| *S. aureus* | 0.03 | 0.13 | 0.32 | 0.05 | <0.001 | 0.01 | 0.94 | 0.16 | 0.23 | 0.52 | 0.08 | 0.53 |
| *E. coli* | 0.68 | 0.01 | 0.03 | <0.001 | <0.001 | 0.02 | <0.01 | 0.23 | 0.14 | <0.01 | <0.01 | 0.44 |

^a^ Fisher’s-Exact-Test

^b^ Wilcoxon-Ranked-Sum-Test

**Table S6b: Univariable associations of environmental variables**

|  | PM_2.5_^b^ | SO_2_ ^b^ | CO ^b^ | BC ^b^ | NO_2_ ^b^ | O_3_ ^b^ | Humidity ^b^ | Air pressure ^b^ | Temperature ^b^ |
| --- | --- | --- | --- | --- | --- | --- | --- | --- | --- |
| Positive sputum culture | <0.001 | <0.001 | 0.64 | 1 | 0.06 | 0.06 | 0.70 | <0.001 | 0.42 |
| High leucocyte count | 0.05 | 0.42 | 0.04 | 0.52 | 0.46 | 0.27 | 0.82 | <0.001 | 0.46 |
| High epithelial cell count | 0.04 | <0.001 | <0.01 | 0.41 | <0.001 | 0.98 | 0.17 | <0.001 | 0.27 |
| Pneumococcal antigen in urine | 0.20 | <0.001 | <0.001 | 0.10 | <0.001 | 0.03 | 0.33 | 0.01 | <0.001 |
| *S. pneumoniae* | 0.01 | <0.001 | <0.001 | 0.87 | <0.001 | <0.01 | 0.06 | <0.001 | 0.04 |
| *H. influenzae* | 0.16 | 0.52 | 0.17 | 0.23 | 0.25 | 0.45 | <0.001 | 0.05 | 0.33 |
| *H. parainfluenzae* | <0.001 | 0.12 | 0.31 | 0.41 | 0.83 | 0.66 | 0.73 | <0.001 | 0.74 |
| *C. albicans* | 0.67 | <0.001 | <0.001 | <0.01 | <0.001 | 0.58 | <0.001 | <0.001 | 0.09 |
| *S. aureus* | 0.63 | 0.09 | 0.18 | 0.03 | 0.13 | 0.82 | 0.20 | 0.90 | 0.02 |
| *E. coli* | <0.01 | 0.18 | 0.31 | 0.12 | 0.04 | 0.13 | 0.75 | 0.08 | 0.75 |

^b^ Wilcoxon-Ranked-Sum-Test

| Variable | Positive sputum culture | | High epithelial  cell count | | High leucocyte  count | | Pneumococcal antigen  in urine | | *S. pneumoniae* | | *H. influenzae* | | *H. parainfluenzae* | | *S. aureus* | | *C. albicans* | | *E. coli* | |
| --- | --- | --- | --- | --- | --- | --- | --- | --- | --- | --- | --- | --- | --- | --- | --- | --- | --- | --- | --- | --- |
|  | **AIC** | **I^a^** | **AIC** | **I^a^** | **AIC** | **I^a^** | **AIC** | **I^a^** | **AIC** | **I^a^** | **AIC** | **I^a^** | **AIC** | **I^a^** | **AIC** | **I^a^** | **AIC** | **I^a^** | **AIC** | **I^a^** |
| Recruiting centre | 5,184.3 |  | 4,279.7 | % | 5,077.6 | % | 1,696.2 | % | 1,251.8 | % | 1,407.0 | % | 1,140.3 | % | 1,155.1 | % | 941.7 | % | 842.9 | % |
| Seasonality | 5,187.2 | no | 4,284.0 | no | 5,077.8 | no | 1,693.1 | yes | 1,240.1 | yes | 1,408.5 | no | 1,142.6 | no | 1,155.2 | no | 945.3 | no | 845.7 | no |
| Treatment setting | 5,186.3 | no | 4,270.2 | yes | 5,058.2 | yes | 1,664.7 | yes | 1,241.8 | no | 1,381.3 | yes | 1,120.9 | yes | 1,155.0 | no | 922.5 | yes | 841.2 | no |
| Prior antibiotic therapy | 5,156.7 | yes | 4,270.0 | no | 5,059.9 | no | 1,659.7 | yes | 1,227.4 | yes | 1,380.9 | no | 1,122.7 | no | 1,156.4 | no | 921.8 | no | 841.4 | no |
| Sputum quality | 5,111.9 | yes | X | X | X | X | X | X | 1,213.2 | yes | 1,358.3 | yes | 1,119.1 | no | 1,152.7 | yes | 918.9 | yes | 844.0 | no |
| CURB-65 >=2 | 5,104.3 | yes | 4,272.2 | no | 5,058.9 | no | 1,653.0 | yes | 1,211.8 | no | 1,360.3 | no | 1,114.2 | yes | 1,150.8 | no | 920.8 | no | 825.8 | yes |
| Age | 5,098.9 | yes | 4,266.1 | yes | 5,059.5 | no | 1,648.0 | yes | 1,195.5 | yes | 1,358.7 | no | 1,109.4 | yes | 1,138.9 | yes | 920.7 | no | 798.9 | yes |
| BMI | 5,093.8 | yes | 4,263.5 | yes | 5,056.9 | no | 1,622.4 | yes | 1,182.5 | yes | 1,358.1 | no | 1,110.9 | no | 1,135.6 | yes | 920.9 | no | 797.5 | no |
| Sex | 5,090.7 | yes | 4,262.8 | no | 5,060.0 | no | 1,624.2 | no | 1,184.4 | no | 1,347.8 | yes | 1,111.2 | no | 1,137.1 | no | 919.9 | no | 789.2 | yes |
| Active smoking | 5,088.9 | no | 4,265.4 | no | 5,059.9 | no | 1,618.2 | yes | 1,176.0 | yes | 1,349.3 | no | 1,111.3 | no | 1,129.6 | yes | 920.9 | no | 790.9 | no |
| Respiratory disease | 5,048.7 | yes | 4,261.2 | yes | 5,059.5 | no | 1,610.3 | yes | 1,170.4 | yes | 1,347.4 | no | 1,110.1 | no | 1,131.4 | no | 895.4 | yes | 791.2 | no |
| Malignancy | 5,050.6 | no | 4,262.5 | no | 5,059.5 | no | 1,612.0 | no | 1,172.1 | no | 1,348.8 | no | 1,110.1 | no | 1,131.6 | no | 897.4 | no | 788.7 | no |
| Influenca vaccination | 5,049.7 | no | 4,263.1 | no | 5,058.8 | no | 1,608.8 | no | 1,171.9 | no | 1,349.7 | no | 1,110.0 | no | 1,131.0 | no | 896.8 | no | 790.1 | no |
| Pneumococcal vaccination | 5,044.2 | yes | 4,262.9 | no | 5,059.5 | no | 1,609.7 | no | 1,171.6 | no | 1,349.2 | no | 1,105.1 | yes | 1,131.6 | no | 896.2 | no | 790.8 | no |
| PM_2.5_ | 5,040.3 | yes | 4,262.7 | no | 5,056.5 | no | 1,611.1 | no | 1,167.0 | yes | 1,343.5 | yes | 1,107.1 | no | 1,131.2 | no | 875.3 | yes | 785.6 | yes |
| SO_2_ | 5,035.1 | yes | 4,263.2 | no | 5,054.3 | yes | 1,603.5 | yes | 1,153.1 | yes | 1,345.2 | no | 1,099.9 | yes | 1,129.0 | no | 835.6 | yes | 784.7 | no |
| CO | 5,036.9 | no | 4,262.6 | no | 5,053.1 | no | 1,579.2 | yes | 1,149.2 | yes | 1,345.2 | no | 1,101.9 | no | 1,129.8 | no | 836.1 | no | 786.8 | no |
| BC | 5,036.7 | no | 4,263.1 | no | 5,056.2 | no | 1,577.4 | no | 1,150.0 | no | 1,344.5 | no | 1,101.6 | no | 1,127.4 | yes | 823.2 | yes | 786.2 | no |
| NO_2_ | 5,035.7 | no | 4,262.5 | no | 5,053.9 | no | 1,577.7 | no | 1,149.2 | no | 1,344.1 | no | 1,101.7 | no | 1,129.4 | no | 817.9 | yes | 783.2 | yes |
| O_3_ | 5,034.7 | no | 4,263.2 | no | 5,055.7 | no | 1,581.2 | no | 1,151.0 | no | 1,345.3 | no | 1,101.7 | no | 1,128.8 | no | 819.6 | no | 783.2 | no |
| Humidity | 5,037.1 | no | 4,262.5 | no | 5,055.8 | no | 1,576.2 | yes | 1,151.2 | no | 1,342.9 | no | 1,101.8 | no | 1,128.7 | no | 815.8 | yes | 782.3 | no |
| Air pressure | 5,034.0 | no | 4,262.1 | no | 5,055.2 | no | 1,577.5 | no | 1,149.3 | no | 1,345.5 | no | 1,101.0 | no | 1,129.4 | no | 817.8 | no | 783.4 | no |
| Temperature | 5,036.9 | no | 4,262.5 | no | 5,055.1 | no | 1,578.2 | no | 1,151.2 | no | 1,345.4 | no | 1,101.8 | no | 1,128.4 | no | 813.5 | yes | 779.8 | yes |

^a^:Included; %: base model; X: not tested

**Table S7: Model building process**

| Table S8a: Model characteristics for positive sputum culture | | | |  | |
| --- | --- | --- | --- | --- | --- |
| *Predictors* | *Odds Ratios* | *Std. Error* | *95% CI^b^* | *p-value* | *Adjusted p-value^c^* |
| (Intercept) | 0.60 | 0.09 | 0.45 - 0.79 | **<0.001** |  |
| Prior antibiotic therapy | 0.71 | 0.06 | 0.61 - 0.83 | **<0.001** | **<0.001** |
| Sputum quality (high) | 1.67 | 0.13 | 1.43 - 1.96 | **<0.001** | **<0.001** |
| CURB-65 ≥ 2 | 1.11 | 0.10 | 0.93 - 1.33 | 0.26 | 0.27 |
| Age^a^ | 1.08 | 0.05 | 0.99 - 1.17 | 0.08 | 0.09 |
| BMI^a^ | 0.90 | 0.03 | 0.84 - 0.96 | **0.003** | **0.007** |
| Sex (female) | 0.86 | 0.06 | 0.75 - 0.98 | **0.028** | **0.039** |
| Any respiratory comorbidity | 2.24 | 0.33 | 1.67 - 3.00 | **<0.001** | **<0.001** |
| Pneumococcal vaccination | 1.29 | 0.13 | 1.06 - 1.57 | **0.01** | **0.02** |
| PM_2.5_^a^ | 0.87 | 0.08 | 0.74 - 1.04 | 0.12 | 0.14 |
| SO₂^a^ | 0.82 | 0.06 | 0.71 - 0.95 | **0.007** | **0.017** |
| Random effects | | | | |  |
| σ^2d^ | 3.29 | | | |  |
| τ00 _recruiting centre_^e^ | 0.20 | | | |  |
| ICC^f^ | 0.06 | | | |  |
| Recruiting centres (N) | 13 | | | |  |
| Observations (N) | 4014 | | | |  |
| Marginal R^2^ / Conditional R^2^ | 0.055 / 0.110 | | | |  |

^a^standardized; ^b^95% Confidence Interval; ^c^adjusted with Benjamin-Hochberg-Procedure;

^d^Residual variance; ^e^Variance of random intercepts across centres; ^f^Intra-class correlation

| Table S8b: Model characteristics for high epithelial cell count | | | | |  |
| --- | --- | --- | --- | --- | --- |
| *Predictors* | *Odds Ratios* | *Std. Error* | *95% CI^b^* | *p-value* | *Adjusted p-value^c^* |
| (Intercept) | 0.25 | 0.09 | 0.13 - 0.51 | **<0.001** |  |
| Treatment setting (inpatient) | 0.70 | 0.06 | 0.58 - 0.84 | **<0.001** | **<0.001** |
| Age^a^ | 1.11 | 0.05 | 1.02 - 1.21 | **0.015** | **0.025** |
| BMI^a^ | 1.08 | 0.04 | 1.01 - 1.17 | **0.028** | **0.039** |
| Any respiratory comorbidity | 0.71 | 0.12 | 0.51 - 0.99 | **0.044** | 0.05 |
| Random effects | | | | |  |
| σ^2d^ | 3.29 | | | |  |
| τ00 _recruiting centre_^e^ | 1.39 | | | |  |
| ICC^f^ | 0.30 | | | |  |
| Recruiting centres (N) | 13 | | | |  |
| Observations (N) | 4014 | | | |  |
| Marginal R^2^ / Conditional R^2^ | 0.010 / 0.303 | | | |  |

^a^standardized; ^b^95% Confidence Interval; ^c^adjusted with Benjamin-Hochberg-Procedure;

^d^Residual variance; ^e^Variance of random intercepts across centres; ^f^Intra-class correlation

| Table S8c: Model characteristics for high leucocyte count | | | | |  |
| --- | --- | --- | --- | --- | --- |
| *Predictors* | *Odds Ratios* | *Std. Error* | *95% CI^b^* | *p-value* | *Adjusted p-value^c^* |
| (Intercept) | 0.71 | 0.19 | 0.42 - 1.21 | 0.209 |  |
| Treatment setting (inpatient) | 1.46 | 0.12 | 1.25 - 1.71 | **<0.001** | **<0.001** |
| SO_2_^a^ | 1.18 | 0.08 | 1.03 -1.35 | **0.015** | **0.025** |
| Random effects | | | | |  |
| σ^2d^ | 3.29 | | | |  |
| τ00 _recruiting centre_^e^ | 0.88 | | | |  |
| ICC^f^ | 0.21 | | | |  |
| Recruiting centres (N) | 13 | | | |  |
| Observations (N) | 4014 | | | |  |
| Marginal R^2^ / Conditional R^2^ | 0.011 / 0.220 | | | |  |

^a^standardized; ^b^95% Confidence Interval; ^c^adjusted with Benjamin-Hochberg-Procedure;

^d^Residual variance; ^e^Variance of random intercepts across centres; ^f^Intra-class correlation

| Table S8d: Model characteristics for pneumococcal antigen in urine | | | | | |
| --- | --- | --- | --- | --- | --- |
| *Predictors* | *Odds Ratios* | *Std. Error* | *95% CI^b^* | *p-value* | *Adjusted p-value^c^* |
| (Intercept) | 0.02 | 0.00 | 0.01 - 0.03 | **<0.001** |  |
| Season: Spring (vs. winter) | 0.97 | 0.24 | 0.60 - 1.59 | 0.91 | 0.91 |
| Season: Summer (vs. Winter) | 2.21 | 0.71 | 1.17 - 4.15 | **0.014** | **0.025** |
| Season: Fall (vs. winter) | 2.94 | 0.80 | 1.72 - 5.02 | **<0.001** | **<0.001** |
| Treatment setting (inpatient) | 2.46 | 0.45 | 1.72 - 3.51 | **<0.001** | **<0.001** |
| Prior antibiotic therapy | 0.66 | 0.11 | 0.47 - 0.92 | **0.015** | **0.025** |
| CURB-65 ≥ 2 | 2.13 | 0.38 | 1.49 - 3.03 | **<0.001** | **<0.001** |
| Age^a^ | 0.88 | 0.08 | 0.74 - 1.04 | 0.13 | 0.15 |
| BMI^a^ | 0.69 | 0.06 | 0.58 - 0.81 | **<0.001** | **<0.001** |
| Active smoking | 1.39 | 0.21 | 1.03 - 1.87 | **0.031** | **0.042** |
| Any respiratory comorbidity | 0.37 | 0.18 | 0.15 - 0.95 | **0.039** | 0.05 |
| SO₂^a^ | 1.09 | 0.18 | 0.79 - 1.50 | 0.59 | 0.62 |
| CO^a^ | 2.42 | 0.41 | 1.74 - 3.37 | **<0.001** | **<0.001** |
| Humidity^a^ | 1.30 | 0.16 | 1.03 - 1.65 | **0.027** | **0.039** |
| Random effects | | | | |  |
| σ^2d^ | 3.29 | | | |  |
| τ00 _recruiting centre_^e^ | 0.10 | | | |  |
| ICC^f^ | 0.03 | | | |  |
| Recruiting centres (N) | 13 | | | |  |
| Observations (N) | 3612 | | | |  |
| Marginal R^2^ / Conditional R^2^ | 0.218 / 0.240 | | | |  |

^a^standardized; ^b^95% Confidence Interval; ^c^adjusted with Benjamin-Hochberg-Procedure;

^d^Residual variance; ^e^Variance of random intercepts across centres; ^f^Intra-class correlation

| Table S8e: Model characteristics for *S. pneumoniae* detection | | | | |  |
| --- | --- | --- | --- | --- | --- |
| *Predictors* | *Odds Ratios* | *Std. Error* | *95% CI^b^* | *p-value* | *Adjusted p-value^c^* |
| (Intercept) | 0.09 | 0.02 | 0.05 - 0.14 | **<0.001** |  |
| Spring (vs. winter) | 1.38 | 0.36 | 0.83 - 2.29 | 0.21 | 0.23 |
| Summer (vs. Winter) | 1.56 | 0.50 | 0.84 - 2.91 | 0.16 | 0.18 |
| Fall (vs. winter) | 1.16 | 0.37 | 0.63 - 2.15 | 0.64 | 0.66 |
| Prior antibiotic therapy | 0.47 | 0.10 | 0.31 - 0.71 | **<0.001** | **0.001** |
| Sputum quality (high) | 1.81 | 0.30 | 1.31 - 2.49 | **<0.001** | **0.001** |
| Age^a^ | 0.80 | 0.07 | 0.68 - 0.95 | **0.009** | **0.02** |
| BMI^a^ | 0.75 | 0.07 | 0.63 - 0.89 | **0.001** | **0.002** |
| Active smoking | 1.54 | 0.25 | 1.12 - 2.13 | **0.008** | **0.017** |
| Any respiratory comorbidity | 0.63 | 0.23 | 0.31 - 1.30 | 0.21 | 0.23 |
| PM_2.5_^a^ | 0.94 | 0.20 | 0.62 - 1.43 | 0.77 | 0.79 |
| SO_2_^a^ | 1.74 | 0.33 | 1.21 - 2.52 | **0.003** | **0.008** |
| CO^a^ | 1.57 | 0.29 | 1.09 - 2.26 | **0.015** | **0.025** |
| Random effects | | | | |  |
| σ^2d^ | 3.29 | | | |  |
| τ00 _recruiting centre_^e^ | 0.23 | | | |  |
| ICC^f^ | 0.06 | | | |  |
| Recruiting centres (N) | 13 | | | |  |
| Observations (N) | 1581 | | | |  |
| Marginal R^2^ / Conditional R^2^ | 0.206 / 0.258 | | | |  |

^a^standardized; ^b^95% Confidence Interval; ^c^adjusted with Benjamin-Hochberg-Procedure;

^d^Residual variance; ^e^Variance of random intercepts across centres; ^f^Intra-class correlation

| Table S8f: Model characteristics for *H. influenzae* detection | | | |  |  |
| --- | --- | --- | --- | --- | --- |
| *Predictors* | *Odds Ratios* | *Std. Error* | *95% CI^b^* | *p-value* | *Adjusted p-value^c^* |
| (Intercept) | 0.21 | 0.03 | 0.15 - 0.28 | **<0.001** |  |
| Treatment setting (inpatient) | 0.42 | 0.06 | 0.31 - 0.57 | **<0.001** | **<0.001** |
| Sputum quality (high) | 2.09 | 0.31 | 1.56 - 2.80 | **<0.001** | **<0.001** |
| Sex (female) | 1.65 | 0.23 | 1.25 - 2.17 | **<0.001** | **0.001** |
| PM_2.5_^a^ | 1.44 | 0.20 | 1.09 - 1.89 | **0.009** | **0.02** |
| Random effects | | | | |  |
| σ^2d^ | 3.29 | | | |  |
| τ00 _recruiting centre_^e^ | 0.03 | | | |  |
| ICC^f^ | 0.01 | | | |  |
| Recruiting centres (N) | 13 | | | |  |
| Observations (N) | 1581 | | | |  |
| Marginal R^2^ / Conditional R^2^ | 0.100 / 0.107 | | | |  |

^a^standardized; ^b^95% Confidence Interval; ^c^adjusted with Benjamin-Hochberg-Procedure;

^d^Residual variance; ^e^Variance of random intercepts across centres; ^f^Intra-class correlation

| Table S8g: Model characteristics for *H. parainfluenzae* detection | | | |  | |
| --- | --- | --- | --- | --- | --- |
| *Predictors* | *Odds Ratios* | *Std. Error* | *95% CI^b^* | *p-value* | *Adjusted p-value^c^* |
| (Intercept) | 0.12 | 0.07 | 0.04 - 0.35 | **<0.001** |  |
| Sputum quality (high) | 0.69 | 0.12 | 0.48 - 0.98 | **0.037** | **0.048** |
| Treatment setting (inpatient) | 0.51 | 0.10 | 0.35 - 0.75 | **0.001** | **0.002** |
| CURB-65 ≥ 2 | 0.70 | 0.16 | 0.44 - 1.09 | 0.11 | 0.13 |
| Age^a^ | 0.81 | 0.07 | 0.67 - 0.97 | **0.021** | **0.032** |
| Pneumococcal vaccination | 0.54 | 0.14 | 0.33 - 0.88 | **0.014** | **0.025** |
| SO_2_^a^ | 0.67 | 0.10 | 0.50 - 0.90 | **0.008** | **0.017** |
| Random effects | | | | |  |
| σ^2d^ | 3.29 | | | |  |
| τ00 _recruiting centre_^e^ | 3.01 | | | |  |
| ICC^f^ | 0.48 | | | |  |
| Recruiting centres (N) | 13 | | | |  |
| Observations (N) | 1581 | | | |  |
| Marginal R^2^ / Conditional R^2^ | 0.067 / 0.512 | | | |  |

^a^standardized; ^b^95% Confidence Interval; ^c^adjusted with Benjamin-Hochberg-Procedure;

^d^Residual variance; ^e^Variance of random intercepts across centres; ^f^Intra-class correlation

| Table S8h: Model characteristics for *C. albicans* detection | | | |  |  |
| --- | --- | --- | --- | --- | --- |
| *Predictors* | *Odds Ratios* | *Std. Error* | *95% CI^b^* | *p-value* | *Adjusted p-value^c^* |
| (Intercept) | 0.01 | 0.01 | 0.003 - 0.03 | **<0.001** |  |
| Treatment modality (inpatient) | 2.77 | 0.64 | 1.76 - 4.35 | **<0.001** | **<0.001** |
| Sputum quality (high) | 0.56 | 0.13 | 0.36 - 0.87 | **0.011** | **0.021** |
| Any respiratory comorbidity | 1.81 | 0.50 | 1.05 - 3.11 | **0.032** | **0.042** |
| SO_2_^a^ | 0.09 | 0.03 | 0.05 - 0.17 | **<0.001** | **<0.001** |
| NO_2_^a^ | 1.90 | 0.55 | 1.08 - 3.34 | **0.025** | **0.038** |
| Humidity^a^ | 1.68 | 0.26 | 1.24 - 2.27 | **0.001** | **0.002** |
| Random Effects | | | | |  |
| σ^2d^ | 3.29 | | | |  |
| τ00 _recruiting centre_^e^ | 2.36 | | | |  |
| ICC^f^ | 0.42 | | | |  |
| Recruiting centres (N) | 13 | | | |  |
| Observations (N) | 1581 | | | |  |
| Marginal R^2^ / Conditional R^2^ | 0.283 / 0.582 | | | |  |

^a^standardized; ^b^95% Confidence Interval; ^c^adjusted with Benjamin-Hochberg-Procedure;

^d^Residual variance; ^e^Variance of random intercepts across centres; ^f^Intra-class correlation

| Table S8i: Model characteristics for *S. aureus* detection | | | |  |  |
| --- | --- | --- | --- | --- | --- |
| *Predictors* | *Odds Ratios* | *Std. Error* | *95% CI^b^* | *p-value* | *Adjusted p-value^c^* |
| (Intercept) | 0.16 | 0.02 | 0.12 - 0.22 | **<0.001** |  |
| Sputum quality (high) | 0.65 | 0.12 | 0.45 - 0.94 | **0.021** | **0.032** |
| Age^a^ | 0.71 | 0.06 | 0.61 - 0.84 | **<0.001** | **<0.001** |
| BMI^a^ | 0.80 | 0.07 | 0.67 - 0.95 | **0.012** | **0.023** |
| Active smoking | 0.61 | 0.11 | 0.43 - 0.87 | **0.006** | **0.014** |
| BC^a^ | 0.77 | 0.10 | 0.60 - 0.99 | **0.041** | 0.05 |
| Random effects | | | | |  |
| σ^2d^ | 3.29 | | | |  |
| τ00 _recruiting centre_^e^ | 0.09 | | | |  |
| ICC^f^ | 0.03 | | | |  |
| Recruiting centres (N) | 13 | | | |  |
| Observations (N) | 1581 | | | |  |
| Marginal R^2^ / Conditional R^2^ | 0.064 / 0.089 | | | |  |

^a^standardized; ^b^95% Confidence Interval; ^c^adjusted with Benjamin-Hochberg-Procedure;

^d^Residual variance; ^e^Variance of random intercepts across centres; ^f^Intra-class correlation

| Table S8j: Model characteristics for *E. coli* detection | | | | | |
| --- | --- | --- | --- | --- | --- |
| *Predictors* | *Odds Ratios* | *Std. Error* | *95% CI^b^* | *p-value* | *Adjusted p-value^c^* |
| (Intercept) | 0.08 | 0.01 | 0.06 - 0.11 | **<0.001** |  |
| CURB-65 ≥ 2 | 1.24 | 0.28 | 0.79 - 1.93 | 0.35 | 0.37 |
| Age^a^ | 2.12 | 0.31 | 1.59 - 2.83 | **<0.001** | **<0.001** |
| Sex (female) | 0.47 | 0.11 | 0.30 - 0.73 | **0.001** | **0.002** |
| PM_2.5_^a^ | 0.37 | 0.10 | 0.21 - 0.65 | **<0.001** | **0.001** |
| NO_2_^a^ | 2.75 | 0.88 | 1.47 - 5.15 | **0.002** | **0.004** |
| Temperature^a^ | 1.51 | 0.27 | 1.06 - 2.14 | **0.021** | **0.032** |
| Random effects | | | | |  |
| σ^2d^ | 3.29 | | | |  |
| τ00 _recruiting centre_^e^ | 0.09 | | | |  |
| ICC^f^ | 0.03 | | | |  |
| Recruiting centres (N) | 13 | | | |  |
| Observations (N) | 1581 | | | |  |
| Marginal R^2^ / Conditional R^2^ | 0.223 / 0.242 | | | |  |

^a^standardized; ^b^95% Confidence Interval; ^c^adjusted with Benjamin-Hochberg-Procedure;

^d^Residual variance; ^e^Variance of random intercepts across centres; ^f^Intra-class correlation

**Table S9a: Results of ensemble feature selection with ranks and score**

|  | *S. pneumoniae* | | *H. influenzae* | | *H. parainfluenzae* | | *S. aureus* | | *C. albicans* | | *E. coli* | |
| --- | --- | --- | --- | --- | --- | --- | --- | --- | --- | --- | --- | --- |
| Rank | **Variable** | **Score** | **Variable** | **Score** | **Variable** | **Score** | **Variable** | **Score** | **Variable** | **Score** | **Variable** | **Score** |
| 1 | SO_2_ | 0.94 | treatment setting | 0.94 | air pressure | 1 | age | 0.9 | SO_2_ | 1 | age | 0.91 |
| 2 | CO | 0.89 | sputum quality | 0.58 | PM_2.5_ | 0.63 | temperature | 0.68 | air pressure | 0.74 | NO_2_ | 0.63 |
| 3 | age | 0.62 | sex | 0.52 | age | 0.48 | BMI | 0.52 | CO | 0.44 | PM_2.5_ | 0.55 |
| 4 | BMI | 0.61 | humidity | 0.49 | treatment setting | 0.42 | sputum quality | 0.45 | treatment setting | 0.35 | CURB65 | 0.54 |
| 5 | O_3_ | 0.58 | CO | 0.48 | pneumococcal vaccination | 0.37 | BC | 0.42 | humidity | 0.34 | air pressure | 0.47 |
| 6 | smoking | 0.53 | age | 0.44 | CO | 0.34 | CO | 0.38 | NO_2_ | 0.33 | BC | 0.43 |
| 7 | NO_2_ | 0.5 | temperature | 0.42 | CURB65 | 0.33 | SO2 | 0.36 | BC | 0.32 | sex | 0.41 |
| 8 | sputum quality | 0.44 | NO_2_ | 0.38 | BC | 0.3 | NO2 | 0.36 | temperature | 0.29 | CO | 0.38 |
| 9 | air pressure | 0.43 | air pressure | 0.37 | SO_2_ | 0.3 | smoking | 0.36 | respiratory disease | 0.27 | O_3_ | 0.36 |
| 10 | prior antibiotic therapy | 0.42 | season (summer) | 0.36 |  |  | CURB65 | 0.35 |  |  |  |  |
| 11 | season (spring) | 0.39 | BC | 0.35 |  |  | humidity | 0.35 |  |  |  |  |
| 12 |  |  |  |  |  |  | season (summer) | 0.34 |  |  |  |  |
| 13 |  |  |  |  |  |  | influenza vaccination | 0.33 |  |  |  |  |

**Table S9b: Results of ensemble feature selection with ranks and score**

|  | Positive sputum culture | | High epithelial cell count | | High leucocyte count | | Pneumococcal antigen in urine | |
| --- | --- | --- | --- | --- | --- | --- | --- | --- |
| Rank | **Feature** | **Score** | **Feature** | **Score** | **Feature** | **Score** | **Feature** | **Score** |
| 1 | sputum quality | 0.95 | NO_2_ | 0.98 | treatment modality | 1,00 | CO | 0.96 |
| 2 | PM_2.5_ | 0.68 | air pressure | 0.95 | air pressure | 0.53 | NO_2_ | 0.75 |
| 3 | air pressure | 0.61 | treatment setting | 0.76 | CO | 0.49 | SO_2_ | 0.7 |
| 4 | respiratory disease | 0.57 | SO_2_ | 0.66 | PM_2.5_ | 0.41 | BMI | 0.66 |
| 5 | SO_2_ | 0.52 | CO | 0.56 | age | 0.4 | treatment setting | 0.56 |
| 6 | age | 0.52 | PM_2.5_ | 0.55 | NO_2_ | 0.34 | temperature | 0.54 |
| 7 | prior antibiotic therapy | 0.48 | temperature | 0.43 | O_3_ | 0.33 | CURB65 | 0.54 |
| 8 | O_3_ | 0.47 | BC | 0.42 | season (spring) | 0.28 | air pressure | 0.48 |
| 9 | BMI | 0.42 | humidity | 0.4 |  |  | BC | 0.48 |
| 10 | NO_2_ | 0.4 |  |  |  |  | O_3_ | 0.44 |

**Figure S1a: Barplot of EFS for positive sputum culture**

Barplot of EFS displaying the feature importance score and composition of the score based on the seven different methods which are listed to the right.

*Low epithelial cell count*

**Figure S1b: Barplot of EFS for high epithelial cell count**

Barplot of EFS displaying the feature importance score and composition of the score based on the seven different methods which are listed to the right.

**Figure S1c: Barplot of EFS for high leucocyte count**

Barplot of EFS displaying the feature importance score and composition of the score based on the seven different methods which are listed to the right.

**Figure 1d: Barplot of EFS for pneumococcal antigen test in urine**

Barplot of EFS displaying the feature importance score and composition of the score based on the seven different methods which are listed to the right.

**Figure S1e: Barplot of EFS for sputum detection of *S. pneumoniae***

Barplot of EFS displaying the feature importance score and composition of the score based on the seven different methods which are listed to the right.

**Figure S1f: Barplot of EFS for sputum detection of *H. influenzae***

Barplot of EFS displaying the feature importance score and composition of the score based on the seven different methods which are listed to the right.

*High leucocyte count*

**Figure S1g: Barplot of EFS for sputum detection of *H. parainfluenzae***

Barplot of EFS displaying the feature importance score and composition of the score based on the seven different methods which are listed to the right.

*Detection of S. pneumoniae*

**Figure S1h: Barplot of EFS for sputum detection of *C. albicans***

Barplot of EFS displaying the feature importance score and composition of the score based on the seven different methods which are listed to the right.

**Figure S1i: Barplot of EFS for sputum detection of *S. aureus***

Barplot of EFS displaying the feature importance score and composition of the score based on the seven different methods which are listed to the right.

**Figure S1j: Barplot of EFS for sputum detection of *E. coli***

Barplot of EFS displaying the feature importance score and composition of the score based on the seven different methods which are listed to the right.

**Table S10a: Sensitivity analysis of exposure time windows for pathogen prevalence (base model)**

| Predictors | Estimate | Std. error | Z-value | P-value | OR | 95% CI low | 95% CI high |
| --- | --- | --- | --- | --- | --- | --- | --- |
| (Intercept) | -0.51 | 0.14 | -3.59 | <0.001 | 0.60 | 0.45 | 0.79 |
| Prior antibiotic therapy | -0.35 | 0.08 | -4.40 | <0.001 | 0.71 | 0.61 | 0.83 |
| Sputum quality (high) | 0.52 | 0.08 | 6.48 | <0.001 | 1.67 | 1.43 | 1.96 |
| CURB-65 >=2 | 0.11 | 0.09 | 1.13 | 0.26 | 1.11 | 0.93 | 1.33 |
| Age | 0.08 | 0.04 | 1.78 | 0.08 | 1.08 | 0.99 | 1.17 |
| BMI | -0.10 | 0.04 | -2.99 | 0.003 | 0.90 | 0.84 | 0.96 |
| Sex (female) | -0.15 | 0.07 | -2.19 | 0.03 | 0.86 | 0.75 | 0.98 |
| Any respiratory comorbidity | 0.81 | 0.15 | 5.43 | <0.001 | 2.24 | 1.67 | 3.00 |
| Pneumococcal vaccination | 0.26 | 0.10 | 2.58 | 0.001 | 1.29 | 1.06 | 1.57 |
| PM_2.5_ (31-day median) | -0.14 | 0.09 | -1.56 | 0.12 | 0.87 | 0.74 | 1.04 |
| SO_2_ (31-day median) | -0.19 | 0.07 | -2.68 | 0.007 | 0.82 | 0.71 | 0.95 |

**Table S10b: Sensitivity analysis of exposure time windows for pathogen prevalence (15-day median for air pollutants)**

| Predictors | Estimate | Std. error | Z-value | P-value | OR | 95% CI low | 95% CI high |
| --- | --- | --- | --- | --- | --- | --- | --- |
| (Intercept) | -0.51 | 0.14 | -3.59 | <0.001 | 0.60 | 0.45 | 0.79 |
| Prior antibiotic therapy | -0.34 | 0.08 | -4.37 | <0.001 | 0.71 | 0.61 | 0.83 |
| Sputum quality (high) | 0.51 | 0.08 | 6.46 | <0.001 | 1.67 | 1.43 | 1.95 |
| CURB-65 >=2 | 0.11 | 0.09 | 1.15 | 0.25 | 1.11 | 0.93 | 1.34 |
| Age | 0.07 | 0.04 | 1.71 | 0.09 | 1.08 | 0.99 | 1.17 |
| BMI | -0.10 | 0.04 | -2.98 | 0.003 | 0.90 | 0.84 | 0.97 |
| Sex (female) | -0.15 | 0.07 | -2.15 | 0.03 | 0.86 | 0.75 | 0.99 |
| Any respiratory comorbidity | 0.82 | 0.15 | 5.60 | <0.001 | 2.28 | 1.71 | 3.04 |
| Pneumococcal vaccination | 0.26 | 0.10 | 2.56 | 0.01 | 1.29 | 1.06 | 1.57 |
| PM_2.5_ (15-day median) | -0.09 | 0.07 | -1.24 | 0.21 | 0.91 | 0.79 | 1.05 |
| SO_2_ (15-day median) | -0.20 | 0.06 | -3.14 | 0.002 | 0.82 | 0.73 | 0.93 |

**Table S10c: Sensitivity analysis of exposure time windows for pathogen prevalence (7-day median for air pollutants)**

| Predictors | Estimate | Std. error | Z-value | P-value | OR | 95% CI low | 95% CI high |
| --- | --- | --- | --- | --- | --- | --- | --- |
| (Intercept) | -0.51 | 0.14 | -3.62 | <0.001 | 0.60 | 0.46 | 0.79 |
| Prior antibiotic therapy | -0.34 | 0.08 | -4.38 | <0.001 | 0.71 | 0.61 | 0.83 |
| Sputum quality (high) | 0.51 | 0.08 | 6.45 | <0.001 | 1.67 | 1.43 | 1.95 |
| CURB-65 >=2 | 0.11 | 0.09 | 1.19 | 0.23 | 1.12 | 0.93 | 1.34 |
| Age | 0.07 | 0.04 | 1.65 | 0.1 | 1.07 | 0.99 | 1.17 |
| BMI | -0.10 | 0.03 | -2.96 | 0.003 | 0.90 | 0.84 | 0.97 |
| Sex (female) | -0.15 | 0.07 | -2.16 | 0.03 | 0.86 | 0.75 | 0.99 |
| Any respiratory comorbidity | 0.87 | 0.15 | 5.95 | <0.001 | 2.39 | 1.80 | 3.19 |
| Pneumococcal vaccination | 0.25 | 0.10 | 2.54 | 0.01 | 1.29 | 1.06 | 1.57 |
| PM_2.5_ (7-day median) | 0.01 | 0.06 | 0.13 | 0.9 | 1.01 | 0.90 | 1.13 |
| SO_2_ (7-day median) | -0.14 | 0.05 | -2.72 | 0.007 | 0.87 | 0.78 | 0.96 |

**Table S11a: Sensitivity analysis of exposure time windows for high leucocyte count (base model)**

| Predictors | Estimate | Std. error | Z-value | P-value | OR | 95% CI low | 95% CI high |
| --- | --- | --- | --- | --- | --- | --- | --- |
| (Intercept) | -0.34 | 0.27 | -1.26 | 0.2 | 0.71 | 0.42 | 1.21 |
| Treatment setting (inpatient) | 0.38 | 0.08 | 4.68 | <0.001 | 1.46 | 1.25 | 1.71 |
| SO_2_ (31-day median) | 0.17 | 0.07 | 2.43 | 0.02 | 1.18 | 1.03 | 1.35 |

**Table S11b: Sensitivity analysis of exposure time windows for high leucocyte count (15-day median for air pollutants)**

| Predictors | Estimate | Std. error | Z-value | P-value | OR | 95% CI low | 95% CI high |
| --- | --- | --- | --- | --- | --- | --- | --- |
| (Intercept) | -0.35 | 0.27 | -1.28 | 0.2 | 0.70 | 0.41 | 1.20 |
| Treatment setting (inpatient) | 0.38 | 0.08 | 4.70 | <0.001 | 1.46 | 1.25 | 1.71 |
| SO_2_ (15-day median) | 0.13 | 0.06 | 2.13 | 0.03 | 1.13 | 1.01 | 1.27 |

**Table S11c: Sensitivity analysis of exposure time windows for high leucocyte count (7-day median for air pollutants)**

| Predictors | Estimate | Std. error | Z-value | P-value | OR | 95% CI low | 95% CI high |
| --- | --- | --- | --- | --- | --- | --- | --- |
| (Intercept) | -0.35 | 0.27 | -1.30 | 0.19 | 0.70 | 0.41 | 1.20 |
| Treatment setting (inpatient) | 0.38 | 0.08 | 4.67 | <0.001 | 1.46 | 1.25 | 1.71 |
| SO_2_ (7-day median) | 0.11 | 0.05 | 2.15 | 0.03 | 1.11 | 1.01 | 1.23 |

**Table S12a: Sensitivity analysis of exposure time windows for pneumococcal antigen in urine (base model)**

| Predictors | Estimate | Std. error | Z-value | P-value | OR | 95% CI low | 95% CI high |
| --- | --- | --- | --- | --- | --- | --- | --- |
| (Intercept) | -4.1 | 0.26 | -15.66 | <0.001 | 0.02 | 0.01 | 0.03 |
| Season (spring) | -0.03 | 0.25 | -0.11 | 0.91 | 0.97 | 0.60 | 1.59 |
| Season (summer) | 0.79 | 0.32 | 2.45 | 0.01 | 2.21 | 1.17 | 4.15 |
| Season (fall) | 1.08 | 0.27 | 3.93 | <0.001 | 2.94 | 1.72 | 5.02 |
| Treatment setting (inpatient) | 0.9 | 0.18 | 4.96 | <0.001 | 2.46 | 1.72 | 3.51 |
| Prior antibiotic therapy | -0.42 | 0.17 | -2.42 | 0.02 | 0.66 | 0.47 | 0.92 |
| CURB-65 >=2 | 0.75 | 0.18 | 4.18 | <0.001 | 2.13 | 1.49 | 3.03 |
| Age | -0.13 | 0.09 | -1.51 | 0.13 | 0.88 | 0.74 | 1.04 |
| BMI | -0.37 | 0.08 | -4.42 | <0.001 | 0.69 | 0.58 | 0.81 |
| Active smoking | 0.33 | 0.15 | 2.15 | 0.03 | 1.39 | 1.03 | 1.87 |
| Any respiratory comorbidity | -0.98 | 0.48 | -2.07 | 0.04 | 0.37 | 0.15 | 0.95 |
| SO_2_ (31-day median) | 0.09 | 0.16 | 0.54 | 0.59 | 1.09 | 0.79 | 1.50 |
| CO (31-day median) | 0.88 | 0.17 | 5.22 | <0.001 | 2.42 | 1.74 | 3.37 |
| Humidity (7-day median) | 0.27 | 0.12 | 2.22 | 0.03 | 1.30 | 1.03 | 1.65 |

**Table S12b: Sensitivity analysis of exposure time windows for pneumococcal antigen in urine (15-day median for air pollutants)**

| Predictors | Estimate | Std. error | Z-value | P-value | OR | 95% CI low | 95% CI high |
| --- | --- | --- | --- | --- | --- | --- | --- |
| (Intercept) | -4.13 | 0.27 | -15.57 | <0.001 | 0.02 | 0.01 | 0.03 |
| Season (spring) | 0.08 | 0.24 | 0.35 | 0.73 | 1.09 | 0.68 | 1.74 |
| Season (summer) | 0.84 | 0.33 | 2.55 | 0.01 | 2.33 | 1.22 | 4.45 |
| Season (fall) | 1 | 0.27 | 3.77 | <0.001 | 2.73 | 1.62 | 4.61 |
| Treatment setting (inpatient) | 0.9 | 0.18 | 4.93 | <0.001 | 2.45 | 1.72 | 3.50 |
| Prior antibiotic therapy | -0.42 | 0.17 | -2.43 | 0.02 | 0.66 | 0.47 | 0.92 |
| CURB-65 >=2 | 0.75 | 0.18 | 4.18 | <0.001 | 2.12 | 1.49 | 3.02 |
| Age | -0.12 | 0.09 | -1.38 | 0.17 | 0.89 | 0.75 | 1.05 |
| BMI | -0.37 | 0.08 | -4.42 | <0.001 | 0.69 | 0.58 | 0.81 |
| Active smoking | 0.34 | 0.15 | 2.2 | 0.03 | 1.40 | 1.04 | 1.88 |
| Any respiratory comorbidity | -1.03 | 0.47 | -2.18 | 0.03 | 0.36 | 0.14 | 0.90 |
| SO_2_ (15-day median) | 0.06 | 0.14 | 0.42 | 0.68 | 1.06 | 0.81 | 1.38 |
| CO (15-day median) | 0.82 | 0.15 | 5.37 | <0.001 | 2.27 | 1.68 | 3.07 |
| Humidity (7-day median) | 0.23 | 0.12 | 1.9 | 0.06 | 1.25 | 0.99 | 1.59 |

**Table S12c: Sensitivity analysis of exposure time windows for pneumococcal antigen in urine (7-day median for air pollutants)**

| Predictors | Estimate | Std. error | Z-value | P-value | OR | 95% CI low | 95% CI high |
| --- | --- | --- | --- | --- | --- | --- | --- |
| (Intercept) | -4.12 | 0.27 | -15.4 | <0.001 | 0.02 | 0.01 | 0.03 |
| Season (spring) | 0.15 | 0.23 | 0.65 | 0.51 | 1.17 | 0.74 | 1.85 |
| Season (summer) | 0.77 | 0.32 | 2.34 | 0.02 | 2.16 | 1.13 | 4.10 |
| Season (fall) | 0.90 | 0.26 | 3.48 | <0.001 | 2.47 | 1.48 | 4.11 |
| Treatment setting (inpatient) | 0.88 | 0.18 | 4.84 | <0.001 | 2.42 | 1.69 | 3.45 |
| Prior antibiotic therapy | -0.41 | 0.17 | -2.38 | 0.02 | 0.66 | 0.47 | 0.93 |
| CURB-65 >=2 | 0.75 | 0.18 | 4.2 | <0.001 | 2.12 | 1.49 | 3.02 |
| Age | -0.12 | 0.09 | -1.33 | 0.18 | 0.89 | 0.75 | 1.06 |
| BMI | -0.38 | 0.08 | -4.49 | <0.001 | 0.69 | 0.58 | 0.81 |
| Active smoking | 0.35 | 0.15 | 2.31 | 0.02 | 1.42 | 1.05 | 1.91 |
| Any respiratory comorbidity | -1.05 | 0.47 | -2.23 | 0.03 | 0.35 | 0.14 | 0.88 |
| SO_2_ (7-day median) | 0.02 | 0.11 | 0.21 | 0.84 | 1.02 | 0.82 | 1.28 |
| CO (7-day median) | 0.73 | 0.14 | 5.22 | <0.001 | 2.08 | 1.58 | 2.75 |
| Humidity (7-day median) | 0.20 | 0.12 | 1.69 | 0.09 | 1.23 | 0.97 | 1.55 |

**Table S12d: Sensitivity analysis of exposure time windows for pneumococcal antigen in urine (15-day median for weather variables)**

| Predictors | Estimate | Std. error | Z-value | P-value | OR | 95% CI low | 95% CI high |
| --- | --- | --- | --- | --- | --- | --- | --- |
| (Intercept) | -4.15 | 0.27 | -15.56 | <0.001 | 0.02 | 0.01 | 0.03 |
| Season (spring) | 0.04 | 0.26 | 0.14 | 0.89 | 1.04 | 0.62 | 1.72 |
| Season (summer) | 0.89 | 0.34 | 2.65 | 0.008 | 2.44 | 1.26 | 4.73 |
| Season (fall) | 1.09 | 0.27 | 3.98 | <0.001 | 2.98 | 1.74 | 5.09 |
| Treatment setting (inpatient) | 0.90 | 0.18 | 4.97 | <0.001 | 2.46 | 1.73 | 3.52 |
| Prior antibiotic therapy | -0.43 | 0.17 | -2.46 | 0.01 | 0.65 | 0.46 | 0.92 |
| CURB-65 >=2 | 0.76 | 0.18 | 4.19 | <0.001 | 2.13 | 1.49 | 3.03 |
| Age | -0.14 | 0.09 | -1.53 | 0.13 | 0.87 | 0.73 | 1.04 |
| BMI | -0.38 | 0.08 | -4.43 | <0.001 | 0.69 | 0.58 | 0.81 |
| Active smoking | 0.32 | 0.15 | 2.11 | 0.03 | 1.38 | 1.02 | 1.86 |
| Any respiratory comorbidity | -0.97 | 0.47 | -2.04 | 0.04 | 0.38 | 0.15 | 0.96 |
| SO_2_ (31-day median) | 0.1 | 0.16 | 0.6 | 0.55 | 1.10 | 0.80 | 1.52 |
| CO (31-day median) | 0.87 | 0.17 | 5.15 | <0.001 | 2.39 | 1.72 | 3.34 |
| Humidity (15-day median) | 0.34 | 0.14 | 2.44 | 0.01 | 1.41 | 1.07 | 1.86 |

**Table S12e: Sensitivity analysis of exposure time windows for pneumococcal antigen in urine (31-day median for weather variables)**

| Predictors | Estimate | Std. error | Z-value | P-value | OR | 95% CI low | 95% CI high |
| --- | --- | --- | --- | --- | --- | --- | --- |
| (Intercept) | -4.05 | 0.27 | -15.08 | <0.001 | 0.02 | 0.01 | 0.03 |
| Season (spring) | -0.11 | 0.26 | -0.43 | 0.67 | 0.89 | 0.53 | 1.50 |
| Season (summer) | 0.69 | 0.34 | 2 | 0.05 | 1.99 | 1.01 | 3.90 |
| Season (fall) | 1.06 | 0.28 | 3.81 | <0.001 | 2.88 | 1.67 | 4.98 |
| Treatment setting (inpatient) | 0.90 | 0.18 | 4.99 | <0.001 | 2.47 | 1.73 | 3.52 |
| Prior antibiotic therapy | -0.43 | 0.17 | -2.47 | 0.01 | 0.65 | 0.46 | 0.92 |
| CURB-65 >=2 | 0.75 | 0.18 | 4.17 | <0.001 | 2.12 | 1.49 | 3.02 |
| Age | -0.13 | 0.09 | -1.49 | 0.14 | 0.88 | 0.74 | 1.04 |
| BMI | -0.37 | 0.08 | -4.42 | <0.001 | 0.69 | 0.58 | 0.81 |
| Active smoking | 0.33 | 0.15 | 2.14 | 0.03 | 1.38 | 1.03 | 1.87 |
| Any respiratory comorbidity | -0.97 | 0.48 | -2.04 | 0.04 | 0.38 | 0.15 | 0.96 |
| SO_2_ (31-day median) | 0.09 | 0.16 | 0.55 | 0.58 | 1.09 | 0.79 | 1.51 |
| CO (31-day median) | 0.86 | 0.17 | 5.07 | <0.001 | 2.35 | 1.69 | 3.27 |
| Humidity (31-day median) | 0.19 | 0.15 | 1.28 | 0.2 | 1.21 | 0.90 | 1.62 |

**Table S13a: Sensitivity analysis of exposure time windows for *S. pneumoniae* detection (base model)**

| Predictors | Estimate | Std. error | Z-value | P-value | OR | 95% CI low | 95% CI high |
| --- | --- | --- | --- | --- | --- | --- | --- |
| (Intercept) | -2.45 | 0.25 | -9.73 | <0.001 | 0.09 | 0.05 | 0.14 |
| Season (spring) | 0.32 | 0.26 | 1.26 | 0.21 | 1.38 | 0.83 | 2.29 |
| Season (summer) | 0.44 | 0.32 | 1.40 | 0.16 | 1.56 | 0.84 | 2.91 |
| Season (fall) | 0.15 | 0.32 | 0.47 | 0.64 | 1.16 | 0.63 | 2.15 |
| Prior antibiotic therapy | -0.75 | 0.21 | -3.60 | <0.001 | 0.47 | 0.31 | 0.71 |
| Sputum quality (high) | 0.59 | 0.16 | 3.63 | <0.001 | 1.81 | 1.31 | 2.49 |
| Age | -0.22 | 0.08 | -2.60 | 0.009 | 0.80 | 0.68 | 0.95 |
| BMI | -0.29 | 0.09 | -3.35 | <0.001 | 0.75 | 0.63 | 0.89 |
| Active smoking | 0.43 | 0.16 | 2.65 | 0.008 | 1.54 | 1.12 | 2.13 |
| Any respiratory comorbidity | -0.46 | 0.37 | -1.24 | 0.21 | 0.63 | 0.31 | 1.30 |
| PM_2.5_ (31-day median) | -0.06 | 0.21 | -0.28 | 0.78 | 0.94 | 0.62 | 1.43 |
| SO_2_ (31-day median) | 0.56 | 0.19 | 2.95 | 0.003 | 1.74 | 1.21 | 2.52 |
| CO (31-day median) | 0.45 | 0.19 | 2.43 | 0.01 | 1.57 | 1.09 | 2.26 |

**Table S13b: Sensitivity analysis of exposure time windows for *S. pneumoniae* detection (15-day median for air pollutants)**

| Predictors | Estimate | Std. error | Z-value | P-value | OR | 95% CI low | 95% CI high |
| --- | --- | --- | --- | --- | --- | --- | --- |
| (Intercept) | -2.43 | 0.26 | -9.50 | <0.001 | 0.09 | 0.05 | 0.15 |
| Season (spring) | 0.32 | 0.23 | 1.41 | 0.16 | 1.38 | 0.88 | 2.16 |
| Season (summer) | 0.38 | 0.32 | 1.18 | 0.24 | 1.46 | 0.78 | 2.75 |
| Season (fall) | 0.06 | 0.31 | 0.19 | 0.85 | 1.06 | 0.58 | 1.93 |
| Prior antibiotic therapy | -0.76 | 0.21 | -3.67 | <0.001 | 0.47 | 0.31 | 0.70 |
| Sputum quality (high) | 0.60 | 0.16 | 3.67 | <0.001 | 1.82 | 1.32 | 2.50 |
| Age | -0.21 | 0.08 | -2.57 | 0.01 | 0.81 | 0.69 | 0.95 |
| BMI | -0.29 | 0.09 | -3.28 | 0.001 | 0.75 | 0.63 | 0.89 |
| Active smoking | 0.44 | 0.16 | 2.69 | 0.007 | 1.55 | 1.13 | 2.14 |
| Any respiratory comorbidity | -0.63 | 0.36 | -1.75 | 0.08 | 0.53 | 0.26 | 1.08 |
| PM_2.5_ (15-day median) | -0.06 | 0.18 | -0.34 | 0.74 | 0.94 | 0.67 | 1.33 |
| SO_2_ (15-day median) | 0.32 | 0.15 | 2.15 | 0.03 | 1.38 | 1.03 | 1.86 |
| CO (15-day median) | 0.44 | 0.17 | 2.65 | 0.008 | 1.56 | 1.12 | 2.16 |

**Table S13c: Sensitivity analysis of exposure time windows for *S. pneumoniae* detection (7-day median for air pollutants)**

| Predictors | Estimate | Std. error | Z-value | P-value | OR | 95% CI low | 95% CI high |
| --- | --- | --- | --- | --- | --- | --- | --- |
| (Intercept) | -2.34 | 0.25 | -9.20 | <0.001 | 0.10 | 0.06 | 0.16 |
| Season (spring) | 0.31 | 0.21 | 1.45 | 0.15 | 1.36 | 0.90 | 2.07 |
| Season (summer) | 0.17 | 0.32 | 0.54 | 0.59 | 1.19 | 0.64 | 2.21 |
| Season (fall) | -0.13 | 0.30 | -0.42 | 0.68 | 0.88 | 0.49 | 1.60 |
| Prior antibiotic therapy | -0.76 | 0.21 | -3.67 | <0.001 | 0.47 | 0.31 | 0.70 |
| Sputum quality (high) | 0.58 | 0.16 | 3.52 | <0.001 | 1.78 | 1.29 | 2.45 |
| Age | -0.22 | 0.08 | -2.64 | 0.008 | 0.80 | 0.68 | 0.95 |
| BMI | -0.30 | 0.09 | -3.40 | <0.001 | 0.74 | 0.63 | 0.88 |
| Active smoking | 0.44 | 0.16 | 2.68 | 0.007 | 1.55 | 1.12 | 2.13 |
| Any respiratory comorbidity | -0.65 | 0.36 | -1.81 | 0.07 | 0.52 | 0.26 | 1.06 |
| PM_2.5_ (7-day median) | 0.02 | 0.14 | 0.13 | 0.9 | 1.02 | 0.78 | 1.33 |
| SO_2_ (7-day median) | 0.24 | 0.12 | 2.00 | 0.05 | 1.27 | 1.00 | 1.60 |
| CO (7-day median) | 0.32 | 0.15 | 2.06 | 0.04 | 1.38 | 1.02 | 1.86 |

**Table S14a: Sensitivity analysis of exposure time windows for *H. influenzae* detection (base model)**

| Predictors | Estimate | Std. error | Z-value | P-value | OR | 95% CI low | 95% CI high |
| --- | --- | --- | --- | --- | --- | --- | --- |
| (Intercept) | -1.58 | 0.15 | -10.44 | <0.001 | 0.21 | 0.15 | 0.28 |
| Treatment setting (inpatient) | -0.86 | 0.15 | -5.64 | <0.001 | 0.42 | 0.31 | 0.57 |
| Sputum quality (high) | 0.74 | 0.15 | 4.92 | <0.001 | 2.09 | 1.56 | 2.80 |
| Sex (female) | 0.50 | 0.14 | 3.54 | <0.001 | 1.65 | 1.25 | 2.18 |
| PM_2.5_ (31-day median) | 0.36 | 0.14 | 2.60 | 0.009 | 1.44 | 1.09 | 1.89 |

**Table S14b: Sensitivity analysis of exposure time windows for *H. influenzae* detection (15-day median for air pollutants)**

| Predictors | Estimate | Std. error | Z-value | P-value | OR | 95% CI low | 95% CI high |
| --- | --- | --- | --- | --- | --- | --- | --- |
| (Intercept) | -1.60 | 0.15 | -10.60 | <0.001 | 0.20 | 0.15 | 0.27 |
| Treatment setting (inpatient) | -0.85 | 0.15 | -5.56 | <0.001 | 0.43 | 0.32 | 0.58 |
| Sputum quality (high) | 0.74 | 0.15 | 4.92 | <0.001 | 2.09 | 1.56 | 2.80 |
| Sex (female) | 0.50 | 0.14 | 3.52 | <0.001 | 1.64 | 1.25 | 2.17 |
| PM_2.5_ (15-day median) | 0.26 | 0.12 | 2.08 | 0.04 | 1.30 | 1.01 | 1.66 |

**Table S14c: Sensitivity analysis of exposure time windows for *H. influenzae* detection (7-day median for air pollutants)**

| Predictors | Estimate | Std. error | Z-value | P-value | OR | 95% CI low | 95% CI high |
| --- | --- | --- | --- | --- | --- | --- | --- |
| (Intercept) | -1.62 | 0.15 | -10.68 | <0.001 | 0.20 | 0.15 | 0.27 |
| Treatment setting (inpatient) | -0.86 | 0.15 | -5.63 | <0.001 | 0.42 | 0.31 | 0.57 |
| Sputum quality (high) | 0.74 | 0.15 | 4.93 | <0.001 | 2.09 | 1.56 | 2.80 |
| Sex (female) | 0.50 | 0.14 | 3.54 | <0.001 | 1.65 | 1.25 | 2.17 |
| PM_2.5_ (7-day median) | 0.29 | 0.10 | 2.89 | 0.004 | 1.33 | 1.10 | 1.62 |

**Table S15a: Sensitivity analysis of exposure time windows for *S. aureus* detection (base model)**

| Predictors | Estimate | Std. error | Z-value | P-value | OR | 95% CI low | 95% CI high |
| --- | --- | --- | --- | --- | --- | --- | --- |
| (Intercept) | -1.81 | 0.14 | -12.53 | <0.001 | 0.16 | 0.12 | 0.22 |
| Sputum quality (high) | -0.43 | 0.19 | -2.30 | 0.02 | 0.65 | 0.45 | 0.94 |
| Age | -0.34 | 0.08 | -4.10 | <0.001 | 0.71 | 0.61 | 0.84 |
| BMI | -0.22 | 0.09 | -2.51 | 0.01 | 0.80 | 0.67 | 0.95 |
| Active smoking | -0.49 | 0.18 | -2.76 | 0.006 | 0.61 | 0.43 | 0.87 |
| BC (31-day median) | -0.26 | 0.13 | -2.04 | 0.04 | 0.77 | 0.60 | 0.99 |

| Predictors | Estimate | Std. error | Z-value | P-value | OR | 95% CI low | 95% CI high |
| --- | --- | --- | --- | --- | --- | --- | --- |
| (Intercept) | -1.79 | 0.14 | -12.55 | <0.001 | 0.17 | 0.13 | 0.22 |
| Sputum quality (high) | -0.44 | 0.19 | -2.33 | 0.02 | 0.65 | 0.45 | 0.93 |
| Age | -0.34 | 0.08 | -4.12 | <0.001 | 0.71 | 0.60 | 0.84 |
| BMI | -0.22 | 0.09 | -2.50 | 0.01 | 0.80 | 0.67 | 0.95 |
| Active smoking | -0.49 | 0.18 | -2.74 | 0.006 | 0.61 | 0.43 | 0.87 |
| BC (15-day median) | -0.16 | 0.12 | -1.37 | 0.17 | 0.85 | 0.67 | 1.07 |

**Table S15b: Sensitivity analysis of exposure time windows for *S. aureus* detection (15-day median for air pollutants)**

**Table S15c: Sensitivity analysis of exposure time windows for *S. aureus* detection (7-day median for air pollutants)**

| Predictors | Estimate | Std. error | Z-value | P-value | OR | 95% CI low | 95% CI high |
| --- | --- | --- | --- | --- | --- | --- | --- |
| (Intercept) | -1.79 | 0.14 | -12.56 | <0.001 | 0.17 | 0.13 | 0.22 |
| Sputum quality (high) | -0.43 | 0.19 | -2.31 | 0.02 | 0.65 | 0.45 | 0.94 |
| Age | -0.34 | 0.08 | -4.12 | <0.001 | 0.71 | 0.60 | 0.84 |
| BMI | -0.22 | 0.09 | -2.51 | 0.01 | 0.80 | 0.67 | 0.95 |
| Active smoking | -0.49 | 0.18 | -2.73 | 0.006 | 0.61 | 0.43 | 0.87 |
| BC (7-day median) | -0.16 | 0.11 | -1.44 | 0.15 | 0.85 | 0.69 | 1.06 |

**Table S16a: Sensitivity analysis of exposure time windows for *C. albicans* detection (base model)**

| Predictors | Estimate | Std. error | Z-value | P-value | OR | 95% CI low | 95% CI high |
| --- | --- | --- | --- | --- | --- | --- | --- |
| (Intercept) | -4.69 | 0.57 | -8.22 | <0.001 | 0.01 | 0.00 | 0.03 |
| Treatment setting (inpatient) | 1.02 | 0.23 | 4.42 | <0.001 | 2.77 | 1.76 | 4.35 |
| Sputum quality (high) | -0.58 | 0.23 | -2.55 | 0.01 | 0.56 | 0.36 | 0.87 |
| Any respiratory comorbidity | 0.59 | 0.28 | 2.15 | 0.03 | 1.81 | 1.05 | 3.11 |
| SO_2_ (31-day median) | -2.42 | 0.32 | -7.51 | <0.001 | 0.09 | 0.05 | 0.17 |
| NO_2_ (31-day median) | 0.64 | 0.29 | 2.24 | 0.03 | 1.90 | 1.08 | 3.34 |
| Humidity (7-day median) | 0.52 | 0.15 | 3.39 | <0.001 | 1.68 | 1.24 | 2.27 |

**Table S16b: Sensitivity analysis of exposure time windows for *C. albicans* detection (15-day median for air pollutants)**

| Predictors | Estimate | Std. error | Z-value | P-value | OR | 95% CI low | 95% CI high |
| --- | --- | --- | --- | --- | --- | --- | --- |
| (Intercept) | -4.57 | 0.57 | -7.98 | <0.001 | 0.01 | 0.00 | 0.03 |
| Treatment setting (inpatient) | 1.02 | 0.23 | 4.48 | <0.001 | 2.77 | 1.77 | 4.32 |
| Sputum quality (high) | -0.56 | 0.23 | -2.49 | 0.01 | 0.57 | 0.37 | 0.89 |
| Any respiratory comorbidity | 0.73 | 0.28 | 2.66 | 0.008 | 2.08 | 1.21 | 3.57 |
| SO_2_ (15-day median) | -1.74 | 0.28 | -6.20 | <0.001 | 0.18 | 0.10 | 0.30 |
| NO_2_ (15-day median) | 0.03 | 0.24 | 0.12 | 0.91 | 1.03 | 0.64 | 1.64 |
| Humidity (7-day median) | 0.58 | 0.16 | 3.70 | <0.001 | 1.78 | 1.31 | 2.42 |

**Table 16c: Sensitivity analysis of exposure time windows for *C. albicans* detection (7-day median for air pollutants)**

| Predictors | Estimate | Std. error | Z-value | P-value | OR | 95% CI low | 95% CI high |
| --- | --- | --- | --- | --- | --- | --- | --- |
| (Intercept) | -4.34 | 0.59 | -7.41 | <0.001 | 0.01 | 0.00 | 0.04 |
| Treatment setting (inpatient) | 1.00 | 0.22 | 4.47 | <0.001 | 2.71 | 1.75 | 4.20 |
| Sputum quality (high) | -0.55 | 0.22 | -2.48 | 0.01 | 0.58 | 0.37 | 0.89 |
| Any respiratory comorbidity | 0.88 | 0.27 | 3.22 | 0.001 | 2.42 | 1.41 | 4.15 |
| SO_2_ (7-day median) | -1.02 | 0.23 | -4.47 | <0.001 | 0.36 | 0.23 | 0.56 |
| NO_2_ (7-day median) | -0.19 | 0.19 | -0.98 | 0.33 | 0.83 | 0.57 | 1.21 |
| Humidity (7-day median) | 0.58 | 0.15 | 3.81 | <0.001 | 1.79 | 1.33 | 2.42 |

**Table S16d: Sensitivity analysis of exposure time windows for *C. albicans* detection (15-day median for weather variables)**

| Predictors | Estimate | Std. error | Z-value | P-value | OR | 95% CI low | 95% CI high |
| --- | --- | --- | --- | --- | --- | --- | --- |
| (Intercept) | -4.69 | 0.57 | -8.18 | <0.001 | 0.01 | 0.00 | 0.03 |
| Treatment setting (inpatient) | 1.02 | 0.23 | 4.44 | <0.001 | 2.78 | 1.77 | 4.36 |
| Sputum quality (high) | -0.59 | 0.23 | -2.56 | 0.01 | 0.56 | 0.36 | 0.87 |
| Any respiratory comorbidity | 0.59 | 0.28 | 2.15 | 0.03 | 1.81 | 1.05 | 3.12 |
| SO_2_ (31-day median) | -2.36 | 0.32 | -7.32 | <0.001 | 0.09 | 0.05 | 0.18 |
| NO_2_ (31-day median) | 0.59 | 0.30 | 1.99 | 0.05 | 1.81 | 1.01 | 3.25 |
| Humidity (15-day median) | 0.54 | 0.17 | 3.18 | 0.001 | 1.72 | 1.23 | 2.41 |

**Table S16e: Sensitivity analysis of exposure time windows for *C. albicans* detection (31-day median for weather variables)**

| Predictors | Estimate | Std. error | Z-value | P-value | OR | 95% CI low | 95% CI high |
| --- | --- | --- | --- | --- | --- | --- | --- |
| (Intercept) | -4.75 | 0.57 | -8.38 | <0.001 | 0.01 | 0.00 | 0.03 |
| Treatment setting (inpatient) | 1.03 | 0.23 | 4.47 | <0.001 | 2.81 | 1.79 | 4.42 |
| Sputum quality (high) | -0.56 | 0.23 | -2.45 | 0.01 | 0.57 | 0.36 | 0.89 |
| Any respiratory comorbidity | 0.54 | 0.28 | 1.95 | 0.05 | 1.72 | 1.00 | 2.96 |
| SO_2_ (31-day median) | -2.26 | 0.32 | -6.97 | <0.001 | 0.10 | 0.05 | 0.20 |
| NO_2_ (31-day median) | 0.24 | 0.33 | 0.72 | 0.47 | 1.27 | 0.66 | 2.44 |
| Humidity (31-day median) | 0.85 | 0.21 | 4.02 | <0.001 | 2.34 | 1.55 | 3.54 |

**Table S17a: Sensitivity analysis of exposure time windows for *E. coli* detection (base model)**

| Predictors | Estimate | Std. error | Z-value | P-value | OR | 95% CI low | 95% CI high |
| --- | --- | --- | --- | --- | --- | --- | --- |
| (Intercept) | -2.52 | 0.18 | -13.77 | <0.001 | 0.08 | 0.06 | 0.11 |
| CURB-65 >=2 | 0.21 | 0.23 | 0.93 | 0.35 | 1.24 | 0.79 | 1.93 |
| Age | 0.75 | 0.15 | 5.12 | <0.001 | 2.12 | 1.59 | 2.83 |
| Sex (female) | -0.76 | 0.23 | -3.33 | <0.001 | 0.47 | 0.30 | 0.73 |
| PM_2.5_ (31-day median) | -0.99 | 0.28 | -3.52 | <0.001 | 0.37 | 0.21 | 0.65 |
| NO_2_ (31-day median) | 1.01 | 0.32 | 3.16 | 0.002 | 2.75 | 1.47 | 5.15 |
| Temperature (7-day median) | 0.41 | 0.18 | 2.31 | 0.02 | 1.51 | 1.06 | 2.14 |

**Table S17b: Sensitivity analysis of exposure time windows for *E. coli* detection (15-day median for air pollutants)**

| Predictors | Estimate | Std. error | Z-value | P-value | OR | 95% CI low | 95% CI high |
| --- | --- | --- | --- | --- | --- | --- | --- |
| (Intercept) | -2.51 | 0.18 | -13.70 | <0.001 | 0.08 | 0.06 | 0.12 |
| CURB-65 >=2 | 0.23 | 0.23 | 1.03 | 0.3 | 1.26 | 0.81 | 1.97 |
| Age | 0.75 | 0.15 | 5.13 | <0.001 | 2.11 | 1.59 | 2.81 |
| Sex (female) | -0.73 | 0.23 | -3.21 | 0.001 | 0.48 | 0.31 | 0.75 |
| PM_2.5_ (15-day median) | -0.66 | 0.23 | -2.84 | 0.005 | 0.51 | 0.33 | 0.81 |
| NO_2_ (15-day median) | 0.83 | 0.24 | 3.39 | <0.001 | 2.29 | 1.42 | 3.70 |
| Temperature (7-day median) | 0.32 | 0.16 | 2.05 | 0.04 | 1.38 | 1.01 | 1.87 |

**Table S17c: Sensitivity analysis of exposure time windows for *E. coli* detection (7-day median for air pollutants)**

| Predictors | Estimate | Std. error | Z-value | P-value | OR | 95% CI low | 95% CI high |
| --- | --- | --- | --- | --- | --- | --- | --- |
| (Intercept) | -2.50 | 0.19 | -13.09 | <0.001 | 0.08 | 0.06 | 0.12 |
| CURB-65 >=2 | 0.24 | 0.23 | 1.05 | 0.29 | 1.27 | 0.81 | 1.99 |
| Age | 0.75 | 0.15 | 5.12 | <0.001 | 2.11 | 1.59 | 2.81 |
| Sex (female) | -0.74 | 0.23 | -3.25 | 0.001 | 0.48 | 0.30 | 0.74 |
| PM_2.5_ (7-day median) | -0.57 | 0.19 | -3.09 | 0.002 | 0.56 | 0.39 | 0.81 |
| NO_2_ (7-day median) | 0.74 | 0.19 | 3.89 | <0.001 | 2.10 | 1.44 | 3.05 |
| Temperature (7-day median) | 0.31 | 0.15 | 2.10 | 0.04 | 1.36 | 1.02 | 1.81 |

**Table S17d: Sensitivity analysis of exposure time windows for *E. coli* detection (15-day median for weather variables)**

| Predictors | Estimate | Std. error | Z-value | P-value | OR | 95% CI low | 95% CI high |
| --- | --- | --- | --- | --- | --- | --- | --- |
| (Intercept) | -2.52 | 0.18 | -14.38 | <0.001 | 0.08 | 0.06 | 0.11 |
| CURB-65 >=2 | 0.23 | 0.23 | 1.01 | 0.31 | 1.26 | 0.80 | 1.97 |
| Age | 0.75 | 0.15 | 5.08 | <0.001 | 2.11 | 1.58 | 2.81 |
| Sex (female) | -0.75 | 0.23 | -3.29 | <0.001 | 0.47 | 0.30 | 0.74 |
| PM_2.5_ (31-day median) | -1.07 | 0.29 | -3.75 | <0.001 | 0.34 | 0.20 | 0.60 |
| NO_2_ (31-day median) | 1.22 | 0.35 | 3.46 | <0.001 | 3.39 | 1.70 | 6.77 |
| Temperature (15-day median) | 0.55 | 0.20 | 2.78 | 0.005 | 1.73 | 1.18 | 2.56 |

**Table S17e: Sensitivity analysis of exposure time windows for *E. coli* detection (31-day median for weather variables)**

| Predictors | Estimate | Std. error | Z-value | P-value | OR | 95% CI low | 95% CI high |
| --- | --- | --- | --- | --- | --- | --- | --- |
| (Intercept) | -2.50 | 0.18 | -13.92 | <0.001 | 0.08 | 0.06 | 0.12 |
| CURB-65 >=2 | 0.22 | 0.23 | 0.96 | 0.34 | 1.24 | 0.79 | 1.95 |
| Age | 0.75 | 0.15 | 5.11 | <0.001 | 2.11 | 1.59 | 2.82 |
| Sex (female) | -0.75 | 0.23 | -3.27 | 0.001 | 0.47 | 0.30 | 0.74 |
| PM_2.5_ (31-day median) | -0.96 | 0.27 | -3.54 | <0.001 | 0.38 | 0.23 | 0.65 |
| NO_2_ (31-day median) | 1.08 | 0.33 | 3.24 | 0.001 | 2.94 | 1.53 | 5.65 |
| Temperature (31-day median) | 0.47 | 0.19 | 2.44 | 0.01 | 1.61 | 1.10 | 2.35 |

**Table S18a: Pooled results after MICE imputation for pathogen prevalence model (n=4446)**

| Predictors | OR | Std. error | Z-value | p-value | 95% CI low | 95% CI high |
| --- | --- | --- | --- | --- | --- | --- |
| Intercept | 0.52 | 0.14 | -4.57 | <0.001 | 0.4 | 0.69 |
| Prior antibiotic therapy | 0.71 | 0.08 | -4.42 | <0.001 | 0.62 | 0.83 |
| Sputum quality (high) | 1.73 | 0.08 | 6.84 | <0.001 | 1.48 | 2.02 |
| CURB-65 >=2 | 1.06 | 0.09 | 0.63 | 0.53 | 0.89 | 1.27 |
| Age | 1.09 | 0.04 | 1.97 | 0.05 | 1 | 1.18 |
| BMI | 0.9 | 0.03 | -3.22 | 0.001 | 0.84 | 0.96 |
| Sex (female) | 0.85 | 0.07 | -2.39 | 0.02 | 0.74 | 0.97 |
| Any respiratory comorbidity | 2 | 0.14 | 5.04 | <0.001 | 1.53 | 2.62 |
| Pneumococcal vaccination | 1.31 | 0.1 | 2.77 | 0.006 | 1.08 | 1.58 |
| PM_2.5_ (31-day median) | 0.9 | 0.08 | -1.32 | 0.19 | 0.76 | 1.06 |
| SO_2_ (31-day median) | 0.82 | 0.07 | -2.95 | 0.003 | 0.71 | 0.93 |

**Table S18b: Variation of imputed variables for pathogen prevalence model (n=4446)**

| Imputation | CURB-65 >=2 (n) | CURB-65 < 2 (n) | Sputum quality (high) (n) | Sputum quality (low) (n) |
| --- | --- | --- | --- | --- |
| 1 | 1,210 | 3,236 | 1,046 | 3,400 |
| 2 | 1,191 | 3,255 | 1,033 | 3,413 |
| 3 | 1,218 | 3,228 | 1,036 | 3,410 |
| 4 | 1,203 | 3,243 | 1,035 | 3,411 |
| 5 | 1,240 | 3,206 | 1,032 | 3,414 |
| 6 | 1,218 | 3,228 | 1,046 | 3,400 |
| 7 | 1,198 | 3,248 | 1,034 | 3,412 |
| 8 | 1,198 | 3,248 | 1,033 | 3,413 |
| 9 | 1,221 | 3,225 | 1,030 | 3,416 |
| 10 | 1,207 | 3,239 | 1,031 | 3,415 |
| 11 | 1,213 | 3,233 | 1,031 | 3,415 |
| 12 | 1,197 | 3,249 | 1,031 | 3,415 |
| 13 | 1,209 | 3,237 | 1,047 | 3,399 |
| 14 | 1,237 | 3,209 | 1,024 | 3,422 |
| 15 | 1,200 | 3,246 | 1,025 | 3,421 |
| 16 | 1,223 | 3,223 | 1,038 | 3,408 |
| 17 | 1,220 | 3,226 | 1,043 | 3,403 |
| 18 | 1,206 | 3,240 | 1,033 | 3,413 |
| 19 | 1,218 | 3,228 | 1,027 | 3,419 |
| 20 | 1,221 | 3,225 | 1,052 | 3,394 |
| Original (n=4014) | 887 | 3,127 | 948 | 3,066 |

**Table S19a: Pooled results after MICE imputation for pneumococcal antigen in urine model (n=3612)**

| Predictors | OR | Std. error | Z-value | p-value | 95% CI low | 95% CI high |
| --- | --- | --- | --- | --- | --- | --- |
| Intercept | 0.02 | 0.27 | -15.50 | <0.001 | 0.01 | 0.03 |
| Season (spring) | 0.98 | 0.25 | -0.10 | 0.92 | 0.60 | 1.60 |
| Season (summer) | 2.17 | 0.32 | 2.40 | 0.02 | 1.15 | 4.10 |
| Season (fall) | 2.93 | 0.28 | 3.91 | <0.001 | 1.71 | 5.02 |
| Treatment setting (inpatient) | 2.41 | 0.18 | 4.82 | <0.001 | 1.69 | 3.45 |
| Prior antibiotic therapy | 0.66 | 0.17 | -2.41 | 0.02 | 0.47 | 0.92 |
| CURB-65 >=2 | 2.17 | 0.19 | 3.99 | <0.001 | 1.48 | 3.17 |
| Age | 0.85 | 0.09 | -1.78 | 0.07 | 0.71 | 1.02 |
| BMI | 0.69 | 0.09 | -4.35 | <0.001 | 0.59 | 0.82 |
| Active smoking | 1.39 | 0.15 | 2.17 | 0.03 | 1.03 | 1.88 |
| Any respiratory comorbidity | 0.38 | 0.48 | -2.01 | 0.04 | 0.15 | 0.98 |
| SO_2_ (31-day median) | 1.07 | 0.16 | 0.41 | 0.68 | 0.78 | 1.47 |
| CO (31-day median) | 2.40 | 0.17 | 5.16 | <0.001 | 1.72 | 3.35 |
| Humidity (7-day median) | 1.31 | 0.12 | 2.25 | 0.02 | 1.03 | 1.66 |

**Table S19b: Variation of imputed variables for pneumococcal antigen in urine model (n=3612)**

| Imputation | CURB-65 ≥ 2 (n) | CURB-65 < 2 (n) |
| --- | --- | --- |
| 1 | 906 | 2,706 |
| 2 | 910 | 2,702 |
| 3 | 921 | 2,691 |
| 4 | 913 | 2,699 |
| 5 | 929 | 2,683 |
| 6 | 923 | 2,689 |
| 7 | 924 | 2,688 |
| 8 | 905 | 2,707 |
| 9 | 917 | 2,695 |
| 10 | 911 | 2,701 |
| 11 | 920 | 2,692 |
| 12 | 922 | 2,690 |
| 13 | 917 | 2,695 |
| 14 | 923 | 2,689 |
| 15 | 903 | 2,709 |
| 16 | 923 | 2,689 |
| 17 | 912 | 2,700 |
| 18 | 924 | 2,688 |
| 19 | 905 | 2,707 |
| 20 | 911 | 2,701 |
| Original (n=3612) | 778 | 2,834 |

**Table S20a: Pooled results after MICE imputation** **for *S. pneumoniae* model (n=1581)**

| Predictors | OR | Std. error | Z-value | p-value | 95% CI low | 95% CI high |
| --- | --- | --- | --- | --- | --- | --- |
| (Intercept) | 0.08 | 0.25 | -9.90 | <0.001 | 0.05 | 0.14 |
| Season (spring) | 1.37 | 0.26 | 1.22 | 0.22 | 0.83 | 2.27 |
| Season (summer) | 1.55 | 0.32 | 1.39 | 0.17 | 0.83 | 2.89 |
| Season (fall) | 1.16 | 0.32 | 0.47 | 0.64 | 0.62 | 2.15 |
| Prior antibiotic therapy | 0.47 | 0.21 | -3.59 | <0.001 | 0.32 | 0.71 |
| Sputum quality (high) | 1.82 | 0.16 | 3.62 | <0.001 | 1.31 | 2.51 |
| Age | 0.80 | 0.08 | -2.61 | 0.009 | 0.68 | 0.95 |
| BMI | 0.75 | 0.09 | -3.33 | <0.001 | 0.63 | 0.89 |
| Active smoking | 1.55 | 0.16 | 2.69 | 0.007 | 1.13 | 2.14 |
| Any respiratory comorbidity | 0.64 | 0.37 | -1.23 | 0.22 | 0.31 | 1.31 |
| PM_2.5_ (31-day median) | 0.94 | 0.21 | -0.31 | 0.76 | 0.62 | 1.42 |
| SO_2_ (31-day median) | 1.74 | 0.19 | 2.96 | 0.003 | 1.21 | 2.52 |
| CO (31-day median) | 1.58 | 0.19 | 2.45 | 0.02 | 1.09 | 2.27 |

**Table S20b: Variation of imputed variables for *S. pneumoniae* model (n=1581)**

| Imputation | Sputum quality (high) (n) | | Sputum quality (low) (n) |
| --- | --- | --- | --- |
| 1 | 493 | 1,088 | |
| 2 | 482 | 1,099 | |
| 3 | 492 | 1,089 | |
| 4 | 486 | 1,095 | |
| 5 | 484 | 1,097 | |
| 6 | 482 | 1,099 | |
| 7 | 491 | 1,090 | |
| 8 | 489 | 1,092 | |
| 9 | 485 | 1,096 | |
| 10 | 489 | 1,092 | |
| 11 | 490 | 1,091 | |
| 12 | 483 | 1,098 | |
| 13 | 494 | 1,087 | |
| 14 | 487 | 1,094 | |
| 15 | 490 | 1,091 | |
| 16 | 482 | 1,099 | |
| 17 | 488 | 1,093 | |
| 18 | 486 | 1,095 | |
| 19 | 491 | 1,090 | |
| 20 | 484 | 1,097 | |
| Original (n=1581) | 467 | 1114 | |

**Table S21a: Pooled results after MICE imputation for *H. influenzae* model (n=1581)**

| Predictors | OR | Std. error | Z-value | p-value | 95% CI low | 95% CI high |
| --- | --- | --- | --- | --- | --- | --- |
| (Intercept) | 0.20 | 0.15 | -10.58 | <0.001 | 0.15 | 0.27 |
| Treatment setting (inpatient) | 0.42 | 0.15 | -5.73 | <0.001 | 0.31 | 0.56 |
| Sputum quality (high) | 2.17 | 0.15 | 5.13 | <0.001 | 1.61 | 2.92 |
| Sex (female) | 1.65 | 0.14 | 3.53 | <0.001 | 1.25 | 2.17 |
| PM_2.5_ (31-day median) | 1.43 | 0.14 | 2.55 | 0.011 | 1.09 | 1.88 |

**Table S21b: Variation of imputed variables for *H. influenzae* model (n=1581)**

| Imputation | Sputum quality (high) (n) | Sputum quality (low) (n) |
| --- | --- | --- |
| 1 | 494 | 1,087 |
| 2 | 486 | 1,095 |
| 3 | 494 | 1,087 |
| 4 | 490 | 1,091 |
| 5 | 485 | 1,096 |
| 6 | 486 | 1,095 |
| 7 | 492 | 1,089 |
| 8 | 492 | 1,089 |
| 9 | 486 | 1,095 |
| 10 | 488 | 1,093 |
| 11 | 490 | 1,091 |
| 12 | 485 | 1,096 |
| 13 | 495 | 1,086 |
| 14 | 488 | 1,093 |
| 15 | 489 | 1,092 |
| 16 | 485 | 1,096 |
| 17 | 490 | 1,091 |
| 18 | 489 | 1,092 |
| 19 | 492 | 1,089 |
| 20 | 488 | 1,093 |
| Original (n=1581) | 467 | 1,114 |

**Table S22a: Pooled results after MICE imputation for *H. parainfluenzae* model (n=1581)**

| Predictors | OR | Std. error | Z-value | p-value | 95% CI low | 95% CI high |
| --- | --- | --- | --- | --- | --- | --- |
| Intercept | 0.13 | 0.48 | -4.31 | <0.001 | 0.05 | 0.32 |
| Sputum quality (high) | 0.72 | 0.18 | -1.86 | 0.06 | 0.50 | 1.02 |
| Treatment setting (inpatient) | 0.52 | 0.20 | -3.32 | <0.001 | 0.35 | 0.77 |
| CURB-65 >=2 | 0.66 | 0.23 | -1.76 | 0.08 | 0.42 | 1.05 |
| Age | 0.82 | 0.10 | -2.08 | 0.04 | 0.68 | 0.99 |
| Pneumococcal vaccination | 0.54 | 0.25 | -2.46 | 0.01 | 0.33 | 0.88 |
| SO_2_ (31-day median) | 0.69 | 0.15 | -2.48 | 0.01 | 0.52 | 0.93 |

**Table S22b: Variation of imputed variables for *H. parainfluenzae* model (n=1581)**

| Imputation | CURB-65 ≥ 2 (n) | CURB-65 < 2 (n) | Sputum quality (high) (n) | Sputum quality (low) (n) |
| --- | --- | --- | --- | --- |
| 1 | 487 | 1,094 | 489 | 1,092 |
| 2 | 480 | 1,101 | 490 | 1,091 |
| 3 | 488 | 1,093 | 484 | 1,097 |
| 4 | 480 | 1,101 | 488 | 1,093 |
| 5 | 476 | 1,105 | 490 | 1,091 |
| 6 | 484 | 1,097 | 486 | 1,095 |
| 7 | 483 | 1,098 | 486 | 1,095 |
| 8 | 468 | 1,113 | 487 | 1,094 |
| 9 | 487 | 1,094 | 485 | 1,096 |
| 10 | 472 | 1,109 | 491 | 1,090 |
| 11 | 489 | 1,092 | 495 | 1,086 |
| 12 | 484 | 1,097 | 489 | 1,092 |
| 13 | 488 | 1,093 | 484 | 1,097 |
| 14 | 488 | 1,093 | 479 | 1,102 |
| 15 | 482 | 1,099 | 483 | 1,098 |
| 16 | 491 | 1,090 | 480 | 1,101 |
| 17 | 471 | 1,110 | 485 | 1,096 |
| 18 | 473 | 1,108 | 488 | 1,093 |
| 19 | 478 | 1,103 | 490 | 1,091 |
| 20 | 483 | 1,098 | 491 | 1,090 |
| Original (n=1581) | 405 | 1,176 | 467 | 1,114 |

**Table S23a: Pooled results after MICE imputation for *C. albicans* model (n=1581)**

| Predictors | OR | Std. error | Z-value | p-value | 95% CI low | 95% CI high |
| --- | --- | --- | --- | --- | --- | --- |
| (Intercept) | 0.01 | 0.57 | -8.19 | <0.001 | 0 | 0.03 |
| Treatment setting (inpatient) | 2.97 | 0.23 | 4.69 | <0.001 | 1.89 | 4.69 |
| Sputum quality (high) | 0.53 | 0.23 | -2.75 | 0.006 | 0.33 | 0.83 |
| Any respiratory comorbidity | 1.97 | 0.28 | 2.41 | 0.02 | 1.13 | 3.42 |
| SO_2_ (31-day median) | 0.08 | 0.34 | -7.68 | <0.001 | 0.04 | 0.15 |
| NO_2_ (31-day median) | 1.87 | 0.29 | 2.12 | 0.03 | 1.05 | 3.33 |
| Humidity (7-day median) | 1.68 | 0.16 | 3.30 | <0.001 | 1.23 | 2.29 |

**Table S23b: Variation of imputed variables for *C. albicans* model (n=1581)**

| Imputation | Sputum quality (high) (n) | Sputum quality (low) (n) |
| --- | --- | --- |
| 1 | 491 | 1,090 |
| 2 | 482 | 1,099 |
| 3 | 493 | 1,088 |
| 4 | 490 | 1,091 |
| 5 | 485 | 1,096 |
| 6 | 486 | 1,095 |
| 7 | 494 | 1,087 |
| 8 | 491 | 1,090 |
| 9 | 484 | 1,097 |
| 10 | 491 | 1,090 |
| 11 | 489 | 1,092 |
| 12 | 484 | 1,097 |
| 13 | 493 | 1,088 |
| 14 | 487 | 1,094 |
| 15 | 489 | 1,092 |
| 16 | 482 | 1,099 |
| 17 | 491 | 1,090 |
| 18 | 486 | 1,095 |
| 19 | 490 | 1,091 |
| 20 | 487 | 1,094 |
| Original (n=1581) | 467 | 1,114 |

**Table S24a: Pooled results after MICE imputation for *E.coli* model (n=1581)**

| Predictors | OR | Std. error | Z-value | p-value | 95% CI low | 95% CI high |
| --- | --- | --- | --- | --- | --- | --- |
| (Intercept) | 0.08 | 0.18 | -13.49 | <0.001 | 0.06 | 0.12 |
| CURB-65 >=2 | 1.03 | 0.24 | 0.12 | 0.9 | 0.64 | 1.66 |
| Age | 2.23 | 0.15 | 5.27 | <0.001 | 1.65 | 3.01 |
| Sex (female) | 0.46 | 0.23 | -3.39 | <0.001 | 0.29 | 0.72 |
| PM_2.5_ (31-day median) | 0.37 | 0.28 | -3.52 | <0.001 | 0.21 | 0.64 |
| NO_2_ (31-day median) | 2.81 | 0.33 | 3.17 | 0.002 | 1.48 | 5.32 |
| Temperature (7-day median) | 1.52 | 0.18 | 2.34 | 0.02 | 1.07 | 2.17 |

**Table S24b: Variation of imputed variables for *E. coli* model (n=1581)**

| Imputation | CURB-65 ≥ 2 (n) | CURB-65 < 2 (n) |
| --- | --- | --- |
| 1 | 466 | 1,115 |
| 2 | 473 | 1,108 |
| 3 | 474 | 1,107 |
| 4 | 483 | 1,098 |
| 5 | 483 | 1,098 |
| 6 | 476 | 1,105 |
| 7 | 474 | 1,107 |
| 8 | 488 | 1,093 |
| 9 | 475 | 1,106 |
| 10 | 473 | 1,108 |
| 11 | 477 | 1,104 |
| 12 | 465 | 1,116 |
| 13 | 469 | 1,112 |
| 14 | 488 | 1,093 |
| 15 | 483 | 1,098 |
| 16 | 483 | 1,098 |
| 17 | 480 | 1,101 |
| 18 | 471 | 1,110 |
| 19 | 488 | 1,093 |
| 20 | 477 | 1,104 |
| Original (n=1581) | 467 | 1,114 |

**Tab S25.: Correlation of columnar values of the air pollutants used in our study compared to ground-level concentration for sites in our study as provided by the Federal Environment Agency (UBA)**

| Location | Parameter | Pearson r | p-value | R² |
| --- | --- | --- | --- | --- |
| Hannover | CO | 0.22 | 0.013 | 0.05 |
| Cottbus | CO | 0.49 | 5.39E-23 | 0.24 |
| Berlin-Charité | CO | 0.44 | 7.93E-10 | 0.19 |
| Berlin-Neukölln | CO | 0.05 | 0.625 | 0.00 |
| Berlin-Heckeshorn | CO | 0.29 | 2.53E-07 | 0.09 |
| Hannover | NO_2_ | 0.51 | 1.12E-09 | 0.26 |
| Cottbus | NO_2_ | 0.64 | 7.03E-64 | 0.41 |
| Berlin-Charité | NO_2_ | 0.42 | 4.74E-09 | 0.17 |
| Berlin-Neukölln | NO_2_ | 0.48 | 3.04E-08 | 0.23 |
| Berlin-Heckeshorn | NO_2_ | 0.41 | 2.33E-13 | 0.17 |
| Hannover | SO_2_ | * | * | * |
| Cottbus | SO_2_ | 0.43 | 3.07E-25 | 0.18 |
| Berlin-Charité | SO_2_ | 0.05 | 0.471 | 0.00 |
| Berlin-Neukölln | SO_2_ | 0.29 | 0.001 | 0.08 |
| Berlin-Heckeshorn | SO_2_ | 0.2 | 0.001 | 0.04 |
| Hannover | O_3_ | 0.07 | 0.449 | 0.00 |
| Cottbus | O_3_ | 0.13 | 0.002 | 0.02 |
| Berlin-Charité | O_3_ | 0.11 | 0.151 | 0.01 |
| Berlin-Neukölln | O_~~3~~_ | 0.43 | 1.09E-06 | 0.18 |
| Berlin-Heckeshorn | O_3_ | 0.19 | 0.001 | 0.03 |

*Data unavailable
